# NSs, the Silencing Suppressor of Tomato Spotted Wilt Orthotospovirus, Interferes With JA-Regulated Host Terpenoids Expression to Attract *Frankliniella occidentalis*

**DOI:** 10.3389/fmicb.2020.590451

**Published:** 2020-12-10

**Authors:** Jiao Du, Xiao-yu Song, Xiao-bin Shi, Xin Tang, Jian-bin Chen, Zhan-hong Zhang, Gong Chen, Zhuo Zhang, Xu-guo Zhou, Yong Liu, De-yong Zhang

**Affiliations:** ^1^College of Plant Protection, Hunan Agricultural University, Changsha, China; ^2^Hunan Academy of Agricultural Sciences, Institute of Plant Protection, Changsha, China; ^3^High & New Technology Research Center of Henan Academy of Sciences, Zhengzhou, China; ^4^Hunan Academy of Agricultural Sciences, Institute of Vegetable, Changsha, China; ^5^Department of Entomology, University of Kentucky, Lexington, KY, United States

**Keywords:** tomato spotted wilt orthotospovirus, NSs, *Frankliniella occidentalis*, monoterpene, insect behavior

## Abstract

Tomato spotted wilt orthotospovirus (TSWV) causes serious crop losses worldwide and is transmitted by *Frankliniella occidentalis* (Pergande) (Thysanoptera: Thripidae). NSs protein is the silencing suppressor of TSWV and plays an important role in virus infection, cycling, and transmission process. In this research, we investigated the influences of NSs protein on the interaction of TSWV, plants, and *F. occidentalis* with the transgenic *Arabidopsis thaliana*. Compared with the wild-type Col-0 plant, *F. occidentalis* showed an increased number and induced feeding behavior on transgenic *Arabidopsis thaliana* expressing exogenous NSs. Further analysis showed that NSs reduced the expression of terpenoids synthesis-related genes and the content of monoterpene volatiles in *Arabidopsis*. These monoterpene volatiles played a repellent role in respect to *F. occidentalis*. In addition, the expression level of plant immune-related genes and the content of the plant resistance hormone jasmonic acid (JA) in transgenic *Arabidopsis* were reduced. The silencing suppressor of TSWV NSs alters the emission of plant volatiles and reduces the JA-regulated plant defenses, resulting in enhanced attractiveness of plants to *F. occidentalis* and may increase the transmission probability of TSWV.

## Introduction

In plant-virus-insect interactions, plants have evolved a complicated defense system against herbivores and viruses, such as the defense of secondary metabolites. For instance, plants emit repellent terpenoids as soon as herbivores damage plants (Dahlin et al., 2015; Magalhaes et al., 2018). In addition, when plants are attacked by herbivores and viruses, the plant hormone jasmonic acid (JA) is rapidly synthesized, to activate the expression of defense compounds such as alkaloids and terpenoids, which directly and indirectly increase plant resistance (De Geyter et al., 2012; Okada et al., 2015; Rondoni et al., 2018). Correspondingly, insect-vector transmitted viruses overcome this system by increasing the capability of their insect vectors to promote virus spread (Luan et al., 2014; Shi et al., 2014, 2018; Yan and Xie, 2015). Some plant viruses can induce the synthesis of terpenoids to change the preference of insect vectors (Eigenbrode et al., 2002; Mann et al., 2012; Chen et al., 2017; Shi et al., 2017). Plant viruses have also evolved effective mechanisms to interfere with the JA-mediated defense response by targeting key proteins to inhibit the JA signaling pathway (De Geyter et al., 2012; Attaran et al., 2014; Cole et al., 2014; Gimenez-Ibanez et al., 2014).

Tomato spotted wilt orthotospovirus (TSWV) is a notorious virus in agriculture worldwide, which has a wide range of host plants, such as pepper, tomato, eggplant, broad bean, and lettuce (Turina et al., 2016). *Arabidopsis* is also the host plant for TSWV (Abe et al., 2011). TSWV is transmitted by thrips in a persistent propagative manner (Wan et al., 2020), of which the *Frankliniella occidentalis* (Pergande) (Thysanoptera: Thripidae) is the main species (Rotenberg et al., 2015; Cao et al., 2018). To resist virus infection, plants often exploit RNA silencing in their defense system, while plant viruses encode many RNA silencing suppressors as a corresponding countermeasure. The NSs protein of TSWV is an RNA silencing suppressor which plays many roles in the TSWV infection, replication, and transmission process (Rotenberg et al., 2015). For example, NSs protein suppressed post-transcriptional gene silencing in plants and interfered with RNA silencing in arthropod cell lines (Takeda et al., 2002; Garcia et al., 2006). In addition, NSs protein has been proven to be necessary in virus accumulation in viruliferous thrips (Margaria et al., 2014).

Recent evidence has shown that the silencing suppressor of plant viruses is involved in JA expression and plant volatile manipulation to promote insect vector survival and viral transmission (Yan and Xie, 2015). For example, 2b protein, the silencing suppressor of cucumber mosaic virus (CMV), was identified to prevent JA-induced degradation of JAZ1, and therefore enhanced odor-dependent attraction of the aphid vector (Wu et al., 2017). Similarly, βC1 protein, the RNA silencing suppressor of tomato yellow leaf curl China virus (TYLCCNV), which interacts with the JA-related transcription factor MYC2 and suppresses the JA-regulated synthesis of terpenoids, results in the promotion of virus transmission by the insect vector whitefly, *Bemisia tabaci* (Salvaudon et al., 2013). Up to now, whether there is a role in TSWV silencing suppressors in reducing plant defenses and promoting the preference and feeding behavior of *F. occidentalis* remains unknown.

To investigate the interaction between NSs protein, the silencing suppressor of TSWV, plants, and *F. occidentalis*, we (1) produced transgenic *Arabidopsis* expressing exogenous NSs genes; (2) compared the host preference and feeding behavior of *F. occidentalis* with wild-type *Arabidopsis* and transgenic *Arabidopsis* expressing NSs; (3) profiled plant volatiles using GC-MS and functionally characterized specific volatile compounds using a Y-tube olfactometer; (4) investigated endogenous hormones of plants; and (5) analyzed differentially expressed genes involved in terpenoid biosynthesis and plant-pathogen interaction using RNA-seq.

## Results

### Preference and Feeding Behavior of *F. occidentalis*

**FIGURE 1:**
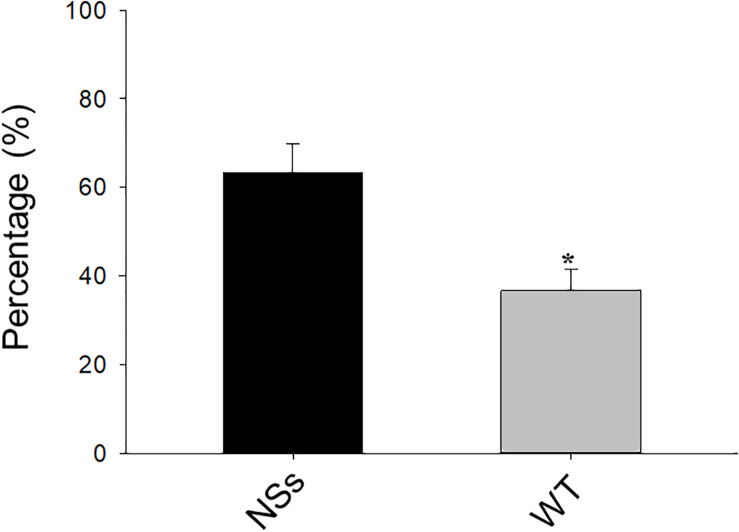
Preference of *F. occidentalis* in Y-tube olfactometer bioassay. WT, wild-type control plants; NSs, Transgenic plants expressing NSs gene. Data are shown as percentages, and * indicate a significant difference between two bars (*n* = 9, Student’s *t-*test, *P* < 0.05).

A two-choice test was conducted with a Y-tube to investigate the preference of *F. occidentalis* on NSs transgenic plants and wild-type plants. The results showed that approximately 64% of *F. occidentalis* preferred the NSs transgenic plants, whereas 36% of *F. occidentalis* preferred wild-type *Arabidopsis* (Student’s *t*-test, *T* = −2.45, df = 16, *P* < 0.01) (Figure 1). *F. occidentalis* preferred the transgenic plants expressing NSs over wild-type *Arabidopsis*.

**FIGURE 2:**
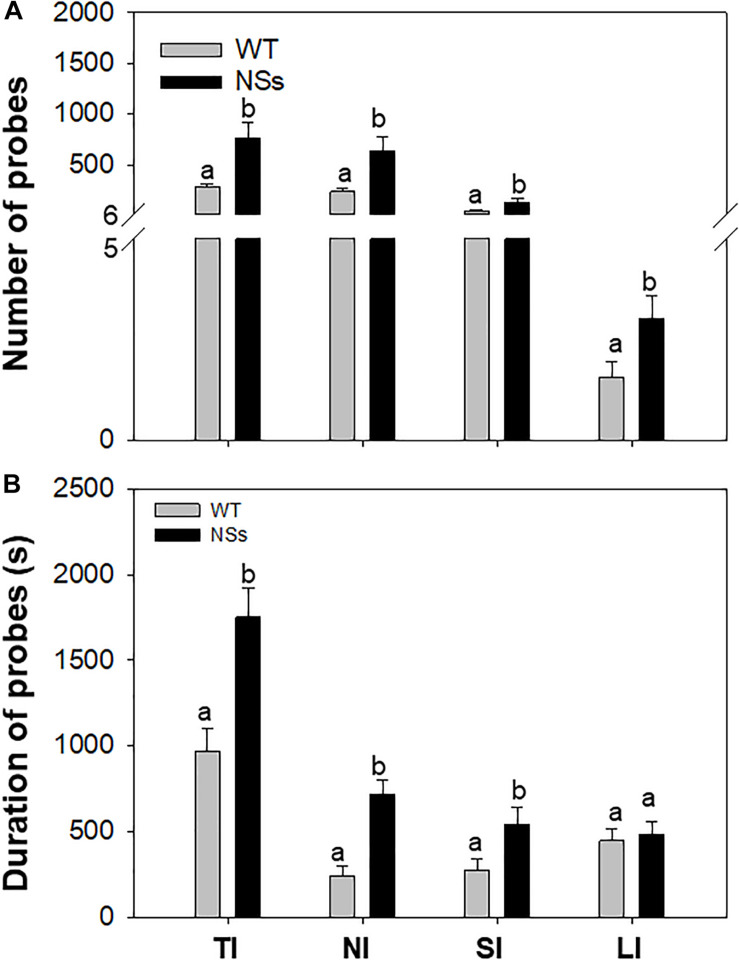
Feeding behavior of *F. occidentalis*. Number and duration were recorded from each of two 1 h period for each individual thrip. **(A)** Number of *F. occidentalis* probes on NSs plants and WT plants; **(B)** Duration of *F. occidentalis* probes on NSs plants and WT plants. WT, wild-type control plants; NSs, transgenic plants expressing NSs gene. TI, total ingestion probes; NI, non-ingestion probes; SI, short-ingestion probes; LI, long-ingestion probes. Different lowercase letters (a, b) indicate significant differences between two bars (*n* = 40, Student’s *t*-test, *P* < 0.05).

An electrical penetration graph (EPG) was used to explore the feeding behavior of *F. occidentalis*. As the results showed, the number of total ingestion probes (TI), non-ingestion probes (NI), short-ingestion probes (SI), and long-ingestion probes (LI) was 2.76, 2.74, 2.90, and 1.93 times greater, respectively, for *F. occidentalis* on NSs plants than on WT plants (TI: Student’s *t*-test, *T* = −2.86, df = 78, *P* < 0.01; NI: Student’s *t*-test, T = −2.68, df = 78, *P* = 0.01; SI: Student’s *t*-test, *T* = −2.73, df = 78, *P* = 0.01; LI: Student’s *t*-test, *T* = −2.14, df = 78, *P* = 0.04; Figure 2A). The duration of the total ingestion probes (TI), non-ingestion probes (NI), short-ingestion probes (SI), and long-ingestion probes (LI) was 1.80, 2.94, 1.95, and 1.09 times greater, respectively, for *F. occidentalis* on NSs plants than on WT plants (TI: Student’s *t*-test, *T* = −3.53, df = 78, *P* < 0.01; NI: Student’s *t*-test, *T* = −4.72, df = 78, *P* < 0.01; SI: Student’s *t*-test, T = −2.28, df = 78, *P* = 0.03; LI: Student’s *t*-test, *T* = −0.38, df = 78, *P* = 0.71; Figure 2B). *F. occidentalis* preferred to feed on NSs plants than on wild-type plants, which means that the silencing suppressor NSs attracts *F. occidentals* to the plants.

### Extraction and Functional Analysis of Plant Volatiles

**FIGURE 3:**
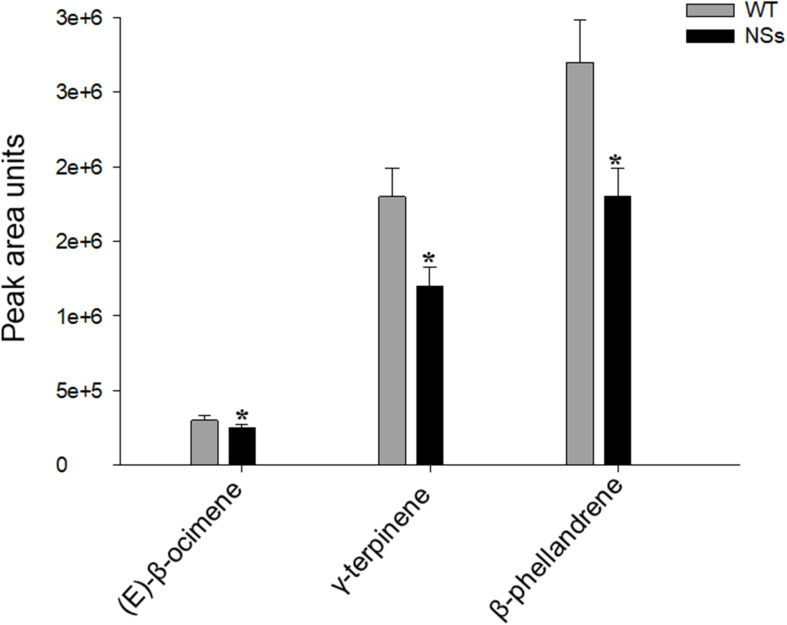
Plant volatiles from wild-type *Arabidopsis* and transgenic plants expressing NSs at the flowering stages. WT, wild-type control plants; NSs, transgenic plants expressing NSs gene. * indicates a significant difference between two bars (*n* = 3, Student’s *t*-test, *P* < 0.05).

The volatiles were measured in the headspace of NSs transgenic plants and wild-type plants. Three terpene volatiles including (E)-β-ocimene, γ-terpinene, and β-phellandrene were detected, and they were all found to be monoterpene. Compared with wild-type *Arabidopsis*, the levels of the three monoterpene volatiles in the transgenic plants expressing NSs were significantly reduced (Student’s *t*-test, *T* = 3.56, df = 4, *P* < 0.05 for (E)-β-ocimene; *T* = 3.37, df = 4, *P* < 0.05 for γ-terpinene; *T* = 4.56, df = 4, *P* < 0.05 for β-phellandrene) (Figure 3). The results indicate that the silencing suppressor NSs attracts *F. occidentals* by suppressing host terpenoids.

### *F. occidentalis* Preference Tests With Volatiles From Arabidopsis

**FIGURE 4:**
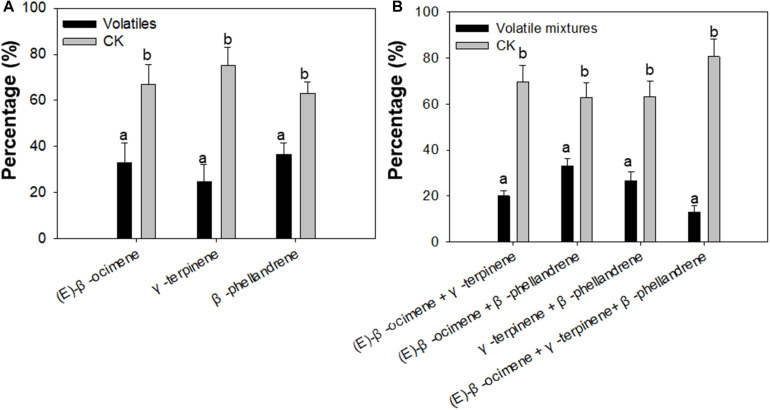
Preference of *F. occidentalis* for selected plant volatiles, including (E)-β-ocimene, β-phellandrene, γ-terpinene, and a mixture of the three volatiles using a Y-tube olfactometer. **(A)** Preference of *F. occidentalis* for respective plant volatiles; **(B)** Preference of *F. occidentalis* for plant volatile mixtures. Standards of volatiles were used in the assay. Volatiles, (E)-β-ocimene or β-phellandrene or γ-terpinene; CK, purified air control; Volatile mixtures, mixture of (E)-β-ocimene, β-phellandrene, γ-terpinene. Different lowercase letters (a, b) indicate a significant difference between two bars (*n* = 9, Student’s *t*-test, *P* < 0.05).

To confirm whether (E)-β-ocimene, γ-terpinene, and β-phellandrene had a repellent effect on *F. occidentalis*, Y-tube olfactory-choice tests of *F. occidentalis* between volatiles and purified air were performed. The number of *F. occidentalis* was significantly higher on the arm of purified air compared with the arm with (E)-β-ocimene, γ-terpinene, and β-phellandrene. Among them, γ-terpinene showed the most obvious repelling effect (Student’s *t*-test, *T* = −3.76, df = 16, *P* < 0.01 for (E)-β-ocimene; *T* = −4.75, df = 16, *P* < 0.01 for γ-terpinene; T = −3.01, df = 16, *P* < 0.05 for β-phellandrene) (Figure 4A).

In each pair of the volatile mixture and control, the number of *F. occidentalis* was higher in the Y-tube arm of purified air control than in the arm of the volatile mixture (Student’s *t*-test, *T* = −6.30, df = 16, *P* < 0.01 for (E)-β-ocimene + γ-terpinene; *T* = −5.06, df = 16, *P* < 0.01 for (E)-β-ocimene + β-phellandrene; T = −8.74, df = 16, *P* < 0.01 for γ-terpinene + β-phellandrene; *T* = −3.50, df = 16, *P* < 0.05 for (E)-β-ocimene + γ-terpinene + β-phellandrene) (Figure 4B).

### Quantification of Plant Endogenous Hormone

**FIGURE 5:**
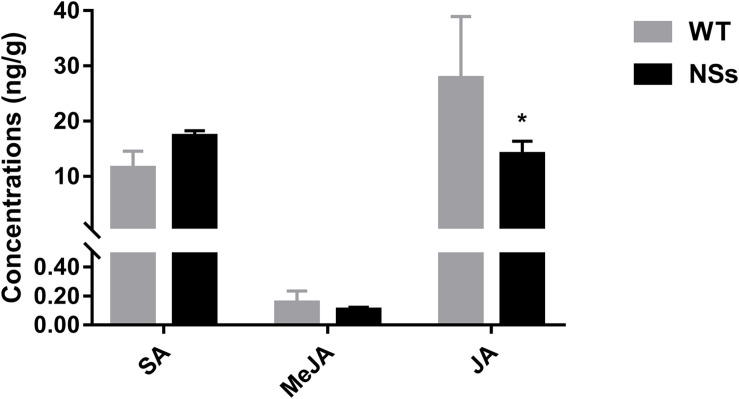
The JA, MeJA, and SA content in wild-type *Arabidopsis* and transgenic *Arabidopsis* expressing NSs at the flowering stages. * indicates a significant difference between two bars (*n* = 3, Student’s *t*-test, *P* < 0.05).

The endogenous hormones were measured in NSs transgenic plants and wild-type plants. Transgenic plants expressing NSs had one third JA compared with wild-type *Arabidopsis* (Student’s *t*-test, *T* = −3.05, df = 4, *P* < 0.05). However, there was no significant difference in the level of MeJA and SA between the wild-type Arabidopsis and transgenic plants expressing NSs (MeJA: Student’s *t*-test, *T* = 0.61, df = 4, *P* > 0.05; SA: Student’s *t*-test, *T* = 1.05, df = 4, *P* > 0.05) (Figure 5). It suggests that NSs interferes with JA-regulated host terpenoid expression to attract *F. occidentals*.

### RNA-Seq of Wild-Type and Transgenic Plants

**FIGURE 6:**
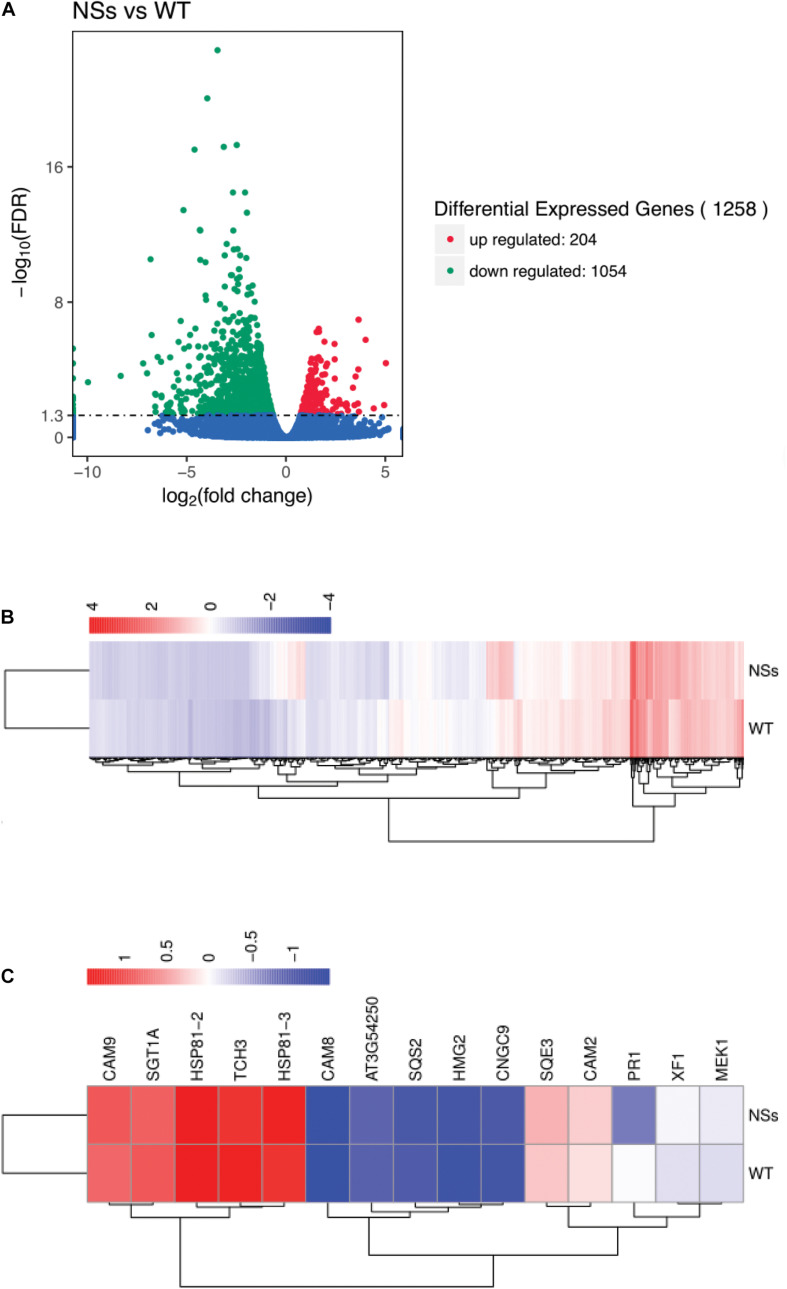
Gene expression profiling by RNA-seq. **(A)** Volcano plot for differentially expressed genes between NSs and WT; **(B)** Cluster analysis of differentially expressed genes; **(C)** Cluster analysis of terpenoid biosynthesis-related genes and plant-pathogen interaction-related genes using RNA-seq data. Total RNA was extracted from transgenic *Arabidopsis* expressing NSs and wild-type *Arabidopsis* and sequenced using the Illumina HiSeq platform. DEGs were defined according to FDR < 0.05. NSs, transgenic plants expressing NSs gene; WT, wild-type plants.

The RNA-seq was performed to confirm the effect of NSs, which suppressed host terpenoids to attract *F. occidentals*. For RNA-seq, an average of 52 million clean reads were produced that mapped onto the *Arabidopsis* genome at an average rate of 92%, representing an average of 16,289 genes that were expressed for each sample (Supplementary Table S2). Compared with wild-type *Arabidopsis*, 204 differentially expressed genes (DEGs) were upregulated and 1054 DEGs were downregulated in transgenic plants expressing NSs (Figures 6A,B and Supplementary Table S3). KEGG analysis indicated that a total of 89 KEGG pathways were enriched for the DEGs (Supplementary Table S4). Most pathways were involved in the plant-pathogen interaction (ath04626), plant hormone signal transduction (ath04075), terpenoid backbone biosynthesis (ath00900), and sesquiterpenoid and triterpenoid biosynthesis (ath00909).

A total of five genes involved in terpenoid biosynthesis were lower in transgenic plants expressing NSs than in wild-type *Arabidopsis* among 1258 DEGs. The five DEGs were identified as terpenoid biosynthesis encoded squalene synthase 2 (SQS2), FAD/NAD (P)-binding oxidoreductase family protein (XF1), squalene epoxidase 3 (SQE3), 3-hydroxy-3-methylglutaryl-CoA reductase 2 (HMG2), and GHMP kinase family protein (AT3G54250) (Figure 6C). In addition, 10 DEGs involved in the plant-pathogen interaction were also identified, including cyclic nucleotide gated channel 9 (CNGC9), calcium-binding EF hand family protein (TCH3), calmodulin 9 (CAM9), calmodulin 2 (CAM2), calmodulin 8 (CAM8), MAP kinase/ERK kinase 1 (MEK1), pathogenesis-related protein 1 (PR1), phosphatase-like protein (SGT1A), heat shock protein 81-2 (HSP81-2), and heat shock protein 81-3 (HSP81-3) (Figure 6C).

### Validation of RNA-Seq Data by qRT-PCR

**FIGURE 7:**
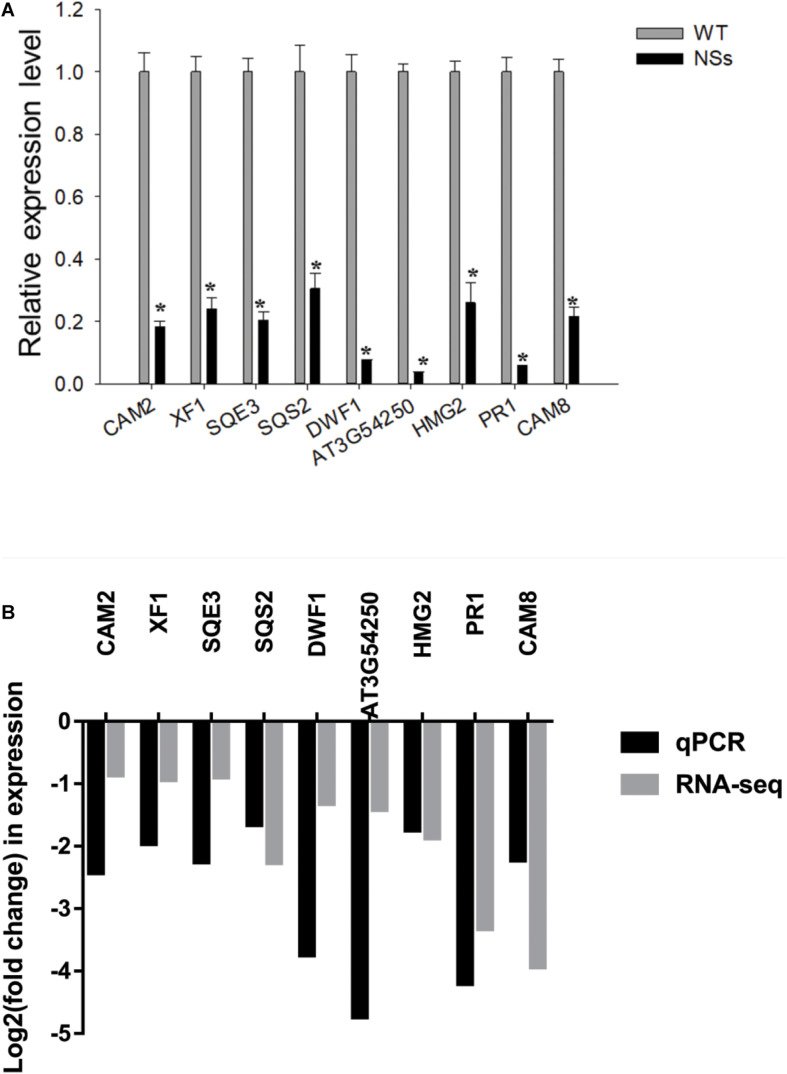
Validation analysis of RNA-seq data by qRT-PCR. **(A)** Quantitative RT-PCR analysis of five selected terpenoid biosynthesis-related and four selected plant-pathogen interaction-related genes. * indicates a significant difference between two bars (*n* = 3, Student’s *t*-test, *P < 0.05); **(B)** The correlation analysis of five selected terpenoid biosynthesis-related and four selected plant-pathogen interaction-related genes between RNA-seq data and qRT-PCR data (Pearson’s correlation test, 0 < r < 1).

The qRT-PCR was conducted to validate RNA-seq data. As expected, compared with wild-type *Arabidopsis*, all nine genes were downregulated in transgenic plants expressing NSs (Figure 7A). To further validate the correlation of RNA-seq data and qRT-PCR data, the r-squared value of Pearson’s correlation test was used. Compared with RNA-seq results, all nine genes (genes in NSs transgenic plants compared to wild-type plants) showed downregulated expressions in the qRT-PCR results (0 < r < 1), which confirmed the reliability of RNA-seq data (Figure 7B). The results of RNA-seq and qRT-PCR confirmed that NSs interferes with JA-regulated host terpenoid expression to attract *F. occidentals*.

## Discussion

During the co-evolution of plant viruses with insect vectors, plant viruses influenced the performance of their vectors by manipulating plant volatile release. Although the specific molecular mechanism by which viruses interfere with the expression of host volatiles is not clear, some effectors have been found to be associated with them (Ziebell et al., 2011; Salvaudon et al., 2013; Casteel et al., 2014). Most effectors belong to silencing suppressors but the function of manipulation is independent of its silencing inhibition activity (Wu et al., 2017). In this study, we reported that NSs, the silencing suppressor of TSWV, reduced the expression of JA and led to a decrease in monoterpene volatile formation, thereby indirectly attracting *F. occidentalis* to plants. In addition, we found that feeding male thrips on NSs plants resulted in an almost threefold increase in the number of total ingestion, non-ingestion, and short-ingestion than on WT plants. Previous research showed that infected males made three times more total probes than uninfected males (Stafford et al., 2011). We found that even without a TSWV infection in the body of the thrip, only an infection of the NSs transgenic plants could induce the feeding behavior of thrips, and NSs may be the key factor of TSWV to induce thrip feeding. The NSs protein of TSWV is an RNA silencing suppressor which plays a key role in TSWV infection (Wu et al., 2019), and here we found a new role for NSs protein in helping the TSWV manipulate the feeding behavior of thrips.

Terpenoids emitted from plants, which are airborne signals for plant defense can repel herbivores (Kendra et al., 2016). Terpenoids are induced when plants are attacked by herbivores in response to herbivore damage and work to repel herbivores (Keeling and Bohlmann, 2006; Heiling et al., 2010). The content of (E)-β-ocimene increased in the volatiles of *Arabidopsis* infested with *Pieris rapae* (Faldt et al., 2003). Headspace volatiles from lima beans infested with spider mites often contain many terpenoids, such as (E)-β-ocimene, 4,8-dimethyl-1,3(E), and 7-non-atriene (Bouwmeester et al., 1999). In addition, γ-terpinene has the acaricidal activity against adult *Hyalomma marginatum* (Cetin et al., 2010). In our research, three monoterpene volatiles were detected: (E)-β-ocimene, β-phellandene, and γ-terpinene, and emissions of these three monoterpene volatiles in the transgenic plants expressing NSs were lower than those of wild-type *Arabidopsis* (Figure 3). Moreover, the Y-tube olfactometer assay showed that *F. occidentalis* was repelled by these three volatiles individually, and the volatile mixtures indicated that plant volatiles act as a repellent to reduce *F. occidentalis* numbers on plants and then reduce damage to *Arabidopsis* (Figure 4). These results indicated that the preference of *F. occidentalis* for transgenic plants expressing NSs was due to the inhibition of monoterpene volatile emissions by NSs. In this research, the terpenoid volatiles detected were different from Wu et al. (2019), as different host plants (Arabidopsis and pepper) were used in the two studies. The volatiles detected were all monoterpenes. It is possible that in different host plants, NSs may reduce the monoterpene synthesis through different volatile pathways to induce the feeding behavior and preference of *F. occidentalis*. Our results indicated that a viral silencing suppressor, such as NSs, plays an important role in inhibiting the synthesis of many kinds of monoterpenes in different host plants.

In addition, RNA-seq and qRT-PCR showed that the reduction of terpenoids was due to the inhibition of the gene expression of terpenoid synthesis pathways by NSs (Figures 6C, 7). The expression of five genes (HMG2, AT3G54250, SQS2, SQE3, and XF1) in the terpenoid synthesis pathway was inhibited. Among them, the enzyme 3-hydroxy-3-methylglutaryl coenzyme A reductase (HMGR), which contains two functionally active HMGR isoforms (HMG1, HMG2), was reported to catalyze the main rate-limiting step in terpenoid biosynthetic pathways (Caelles et al., 1989; Enjuto et al., 1994). For example, the levels of triterpenes were reduced by 65 and 25% in HMG1 and HMG2 mutants, compared to those in wild-type plants, respectively (Ohyama et al., 2007). Moreover, the expression level of sterol in transgenic *Arabidopsis* overexpressing HMGR was increased (Manzano et al., 2004).

Jasmonic acid is the master switch in plant defense systems against herbivores and viruses, activating gene expression associated with terpenoid volatiles synthesis (Okada et al., 2015). For example, caterpillars (*Spodoptera littoralis)* feeding on lima beans increases the expression of JA and induces the synthesis and emission of terpenoids such as (E)-β-ocimene (Arimura et al., 2008). Conversely, after tomato mutant plants (def-1), which are deficient in JA biosynthesis, were attacked by phytophagous mites, the production of terpenoids did not increase as in wild tomatoes (Ament et al., 2004). Moreover, JA and SA have an obvious antagonistic relationship in terpenoid biosynthesis, when *Arabidopsis* is infested with aphids (Girling et al., 2008). SA interferes with JA’s positive regulation of terpenoids synthesis. However, in our study, NSs reduced the expression of JA in plants but had no effect on SA (Figure 5). These results suggest that the synthesis of terpenoids may be regulated by JA signaling pathways.

In conclusion, our study shows that the silencing suppressor NSs of TSWV reduces the emission of plant monoterpene volatiles, to increase the attraction of plants to *F. occidentalis*, by interfering with JA-regulated plant defense systems and reducing the resistant volatiles. Insect vectors are extremely important for the epidemic of their transmitted viruses, since the spread of viruses between plants requires the transport of insect vectors. These viruses commonly change the physiology of plants to increase the attractiveness and adaptability of plants to insect vectors (Beanland et al., 2000; Lacroix et al., 2005; Jiu et al., 2007; Wang et al., 2012; Luan et al., 2014). The phenomenon that viruses alter plant volatiles to increase the attraction of plants to insect vectors is also found in other plant-virus-insect interactions (Eigenbrode et al., 2002; Mann et al., 2012; Chen et al., 2017). Combining these results, we speculate that “odor manipulation” is a common strategy for plant viruses to indirectly promote their own transmission.

## Materials and Methods

### Thrip Strain and TSWV Inoculation

TSWV was obtained in Kunming, Yunnan Province of China from tomato plants in 2018. Then TSWV was purified, identified, designated as TSWV-YN, and mechanically inoculated on *Nicotiana tabacum* cv. Samsun NN. The mechanical inoculation of TSWV involved an inoculum consisting of infected leaf sap in 0.1 M phosphate buffer, 0.2% sodium sulfite and 0.01 M mercaptoethanol, and 1% each of celite 545 and carborundum 320 grit. Cotton swabs were used to draw inoculum and gently rub the fresh leaves of the plant (Mandal et al., 2008; Shalileh et al., 2016). The symptoms were observed after 7–14 days, and the reverse transcription polymerase chain reaction (RT-PCR) method was used to detect whether the plant was successfully infected with the TSWV. The specific primers were NSs-F: ATGTCTTCAAGTGTTTATGAGT and NSs-R: TTATTTTGATCCTGAAGCATATG. To maintain the TSWV strain, some of the TSWV isolates were flash frozen with liquid nitrogen and then placed in a refrigerator at −80°C. The other TSWV isolates were maintained on *N. tabacum* plants by thrip transmission to avoid the viral mutants and the reduced transmissibility.

An isolate of *F. occidentalis* was obtained from Dr. Qing-jun Wu of the Chinese Academy of Agricultural Sciences (Beijing, China). Virus-free stock colonies of *F. occidentalis* were reared on bean pods (*Phaseolus vulgarisin*) in glass jars, closed on top with a 64 μm thrip-proof nylon net in a greenhouse at 25 ± 1°C, 60 ± 10% RH, and a 14 L: 10 D photoperiod. The bean pods were replaced daily, and the harvested pods with eggs were transferred to new glass jars to synchronize larvae growth. *F. occidentalis* used in all the experiments were from synchronized rearing (Mandal et al., 2008).

Wild-type Col-0 *Arabidopsis* was also obtained from the Chinese Academy of Agricultural Sciences (Beijing, China). Wild-type and transgenic *Arabidopsis* were grown in a greenhouse at a temperature of 22 ± 1°C, relative humidity of 55 ± 10%, and a photoperiod of 14 h.

### Construction of Plasmids and Generation of Transgenic Plants

To obtain the NSs ORF, total RNA was extracted from 100 mg of TSWV-infected *N. benthamiana* leaves using Trizol (Invitrogen, United States). The NSs cDNA was obtained by RT-PCR with specific primers using a HiScript II 1st Strand cDNA Synthesis Kit (Vazyme, China). The full length coding sequence of NSs was amplified using pCB-NSs-F (*Xba*I) and pCB-NSs-R (*Pst*I) and inserted between the *Xba*I and *Pst*I sites of the pCambia1301 vector under the control of the cauliflower mosaic virus (CaMV) 35S promoter to generate pCambia1301-NSs. The obtained expression vector was verified by PCR and sequencing. pCambia1301-NSs was transferred into *Agrobacterium tumefaciens* GV3101 by electroporation, and then transferred into Col-0 plants by the floral dip method (Clough and Bent, 2010). Successfully transformed T1 plants were obtained on MS medium containing 50 μg/mL hygromycin B and confirmed by RT-PCR (Supplementary Figure S1A). Therefore, the T3 transgene-homozygote lineages generated from T1 plants through continuous self-pollination (selfing), and RT-PCR identification were used for the experiments. Western blot analysis was used to check for protein expression in the transgenic plants. Total protein of *Arabidopsis thaliana* was extracted using a Plant Protein Extraction Kit (Solarbio, China). Then, the protein was resolved by SDS-PAGE and transferred to a PVDF Membrane (BIO-RAD, United States). The membrane was blocked with TBS-T containing 5% skimmed milk, and incubated with the polyclonal antibody of TSWV NSs protein. Detection was performed incubating with the horseradish peroxidase-conjugated goat anti-rabbit IgG antibody (Thermo Fisher Scientific, United States) and the Pierce^TM^ ECL Plus Western Blotting Substrate (Thermo Fisher Scientific, United States) (Supplementary Figure S1B). Wild-type *Arabidopsis* was used as a negative control. Tubulin was used as a loading control.

### Preference and Feeding Behavior of *F. occidentalis*

According to previous references, the volatile components are mainly released from flowers (Aharoni et al., 2003). To investigate the preference of *F. occidentalis* on wild-type *Arabidopsis* and transgenic plants expressing NSs, plants at the flowering stages were used for pair-wise comparison using the Y-tube. In each preference test, five wild-type plants and five transgenic plants expressing NSs were placed into two odor source bottles, which were connected to the two arms of the Y-tube (stem 10 cm; arms 20 cm, 60° angle; inner diameter 2 cm). Under the action of the gas generator pump, two streams of purified airflow were metered into the arms of the Y-tube at 100 mL/min^–1^.

*F. occidentalis* adults were used in the preference test for approximately 5 days. Thrips were starved for 8 h and were individually introduced at the end of the Y-tube stem. In each test there were 50 thrips, and each thrip was observed for a maximum of 5 min. A “choice” was recorded when a thrip entered one arm for more than 3 cm and a “no choice” was recorded when they remained inactive for 5 min. In each test, the preference of 50 thrips was counted and the number at each arm was recorded. In total, there were nine replicate tests, and there was a total of 450 thrips used in this research. The position of two odor-source bottles was changed to eliminate the effect of potential asymmetry (Cao et al., 2019). The Y-tube was replaced every 10 thrip tests, and in each preference test, the Y-tube was replaced 5 times. The used Y-tube was then washed with 75% ethanol and placed in a 65°C oven to dry.

The feeding behavior of *F. occidentalis* on wild-type *Arabidopsis* and transgenic plants expressing NSs was compared by electrical penetration graphs using a DC-system (a Giga-8 DC-amplifier with a 10^9^ –Ω input resistance, Wageningen University, Netherlands) (Liu et al., 2013). There were two treatments, with each of 20 replicate plants in five to six-leaf stages. The 5 d-old male thrips were cooled on a glass dish on an ice-pack before recording. After that, a 15 μm diameter, 2 cm long gold wire was attached to the thorax of a thrip with a drop of water-soluble silver glue. Each wired thrip was connected to the Giga-8 probe input and then placed on the surface of the back of upper leaf of a plant. The thrip feeding behavior was analyzed with a DI710-UL analog-to-digital converter (Dataq Instruments, Akron, OH) and the output was acquired and stored with the PROBE3.4 software (Wageningen University, Netherlands). EPGs were observed and recorded continuously for 8 h with each thrip. Waveforms of EPG were identified according to a previous publication (Stafford et al., 2011). Each 8 h observation period was divided into two 4 h halves, and a 1 h period from each half was randomly selected for analysis. For each selected 1 h period, the single and total number and duration of non-ingestion, short-ingestion, and long-ingestion probes were measured.

### Extraction and Analysis of Plant Volatiles

Volatiles emitted by *Arabidopsis* at the flowering stages were collected using a dynamic headspace collection system. The soil with the roots of each plant was carefully wrapped in aluminum foil, and five plants were placed in 4 L glass jars with gas inlets and outlets. One stream of purified airflow was metered into a glass jar at 300 mL/min^–1^ from the inlet, and a glass tube filled with 300 mg of PoraPak Q 80/100 mesh (Waters, United States) was used to trap plant volatiles at the outlet. Volatiles were eluted with 800 μL n-hexane (Sigma Aldrich, United States) that contained 160 ng of n-dodecane as an internal standard after 8 h collection under continuous light. Then a 1 μL sample of the solution was subjected to GC/MS analysis. The whole experiment was repeated three times.

GC analysis was performed using a DB-1 (Agilent Technologies, United States) column (30 m × 0.25 mm × 0.25 μm). The temperature profile was as follows: 60°C for 2 min; then increased to 130°C at a programmed rate of 5°C/min^–1^ and kept for 2 min, followed by a rate of 5°C/min^–1^ to 180°C, then followed by rate of 20°C/min^–1^ to 250°C and kept for 5 min. MS conditions were as follows: the temperature of the ion source was 200°C; the scan mass range was 40–500 U. Then the compounds were identified by comparison of GC retention times with those of authentic standards and by comparison of mass spectra with spectra of the National Institute of Standards and Technology (NIST) database. The peak area of the volatile expressed as a proportion of the peak area of the internal standard was used for quantification.

### *F. occidentalis* Preference Tests With Volatiles From Arabidopsis

Based on the GC/MS analysis results, the preference of male *F. occidentalis* to plant volatiles was tested in a Y-tube olfactometer using the standard chemical of detected volatiles (Sigma Aldrich, United States). In the Y-tube olfactometer bioassay, two glass containers, one of the standard chemical and one of purified air as control, were connected into the olfactometer arms (Shi et al., 2018). The preference of *F. occidentalis* was observed as described in “Preference and feeding behavior of *F. occidentalis.*”

### Quantification of Plant Endogenous Hormone

Transgenic plants and wild plants of *Arabidopsis* at the flowering stages were used for quantification of the plant endogenous hormone with 1 g/plant. Leaves of *Arabidopsis* were ground with 10 mL isopropanol/hydrochloric acid and shaken at 4°C for 30 min (You et al., 2016). There were 9 transgenic plants and 9 wild plants. Subsequently, 20 mL dichloromethane was added. The mixture was shaken at 4°C for 30 min and centrifuged at 13,000 rpm at 4°C for 5 min. The organic fraction was separated and then dried under nitrogen in darkness. The solid residue was re-suspended in 400 μL methanol/0.1% methanoic acid. The sample was filtered with a 0.22 μm filter membrane before HPLC-MS/MS analysis.

HPLC analysis was performed using a poroshell 120 SB-C18 (Agilent, United States) column (150 × 2.1 mm × 2.7 μm). The mobile phase A solvents consisted of methanol + 0.1% methanoic acid and the mobile phase B solvents consisted of ultrapure water + 0.1% methanoic acid. The injection volume was 2 μL. MS conditions were as follows: the spray voltage was 4,500 V; the pressure of the air curtain, nebulizer, and aux gas were 15, 65, and 70 psi, respectively, and the atomizing temperature was 400°C.

### RNA-Seq of Wild-Type and Transgenic Plants

Total RNA was extracted from wild-type *Arabidopsis* and transgenic plants (above-ground parts) expressing NSs using Trizol (Invitrogen, United States), respectively. There were three biological replicates of NSs plants and three biological replicates of control wild-type plants. Sequencing libraries were constructed using the NEBNext^®^ UltraTM RNA Library Prep Kit for Illumina^®^ (NEB, United States) and sequenced using the Illumina HiSeq platform. To obtain clean reads, sequencing data of the raw reads were firstly processed through in-house perl scripts. Clean reads were obtained by removing reads containing adapter, reads containing ploy-N, and low quality reads from raw data. At the same time, Q20, Q30, and GC content clean data were calculated. All the downstream analyses were based on the clean data with high quality.

The paired-end clean reads were mapped to the reference genome downloaded from the *Arabidopsis* information resource^1^. To quantify the gene expression level, the mapped clean reads were calculated and then normalized into transcripts per million (TPM) (Patro et al., 2017). Genes with a false discovery rate (FDR < 0.05) were assigned as differentially expressed using the DESeq R package (1.18.0). Gene ontology (GO) enrichment analysis of differentially expressed genes was implemented by the GOseq R package, GO terms with an FDR less than 0.05 were considered significantly enriched by differential expressed genes. With a cut-off of 0 < FDR < 1, the statistical enrichment of differential expression genes was tested by the KOBAS software in Kyoto Encyclopedia of Genes and Genomics (KEGG) pathways^2^.

### Validation of RNA-Seq Data by qRT-PCR

To validate the results from RNA-seq data, we selected nine genes from the terpenoid biosynthesis-related genes and plant-pathogen interaction genes for qRT-PCR analysis. First-strand cDNA was synthesized from RNA using the HiScript^®^ II 1st Strand cDNA Synthesis Kit (Vazyme, China). The specific primers for qRT-PCR were designed using qPrimerDB (Lu et al., 2018)^3^ and two reference genes, β-TUBULIN-2 and ACTIN1 were used as controls for constant transcript level expression (Wang et al., 2009; Ramadoss et al., 2018). The stable expression of β-TUBULIN-2 and ACTIN1 were determined under our experimental conditions. A total of 0.5 μl 10 μM primers were used in the 20 μl qRT-PCR reaction system, the primer sequences are listed in Supplementary Table S1. In addition, qRT-PCR was performed using the AceQ qRT-PCR SYBR-Green Master Mix (Vazyme, China) and was analyzed using the 2^–ΔΔCt^ analysis method (Livak and Schmittgen, 2001). In total, there were three biological replicates and three technical replicates per treatment in this test.

The r-squared value of Pearson’s correlation test was used to validate the correlation of RNA-seq data and qRT-PCR data. When the r-squared value is 0 < R < 1, it means there is a positive correlation between RNA-seq data and qRT-PCR data.

### Data Analysis

All proportional data were arcsine-square root transformed before analyses. Student’s *t*-test for independent samples was used to compare the preference of *F. occidentalis*, the feeding behavior of *F. occidentalis*, the volatile, plant hormone, and qRT-PCR performed on wild-type *Arabidopsis* and transgenic plants expressing NSs. Student’s *t*-test for independent samples was also used to compare *F. occidentalis* preference to volatiles and volatile mixtures from *Arabidopsis*. Pearson’s correlation test was used to validate the correlation of RNA-seq data and qRT-PCR data. SPSS 22.0 (SPSS Software, United States) was used for all statistical analyses.

## Data Availability Statement

The original contributions presented in the study are publicly available. This data can be found here: https://www.ncbi.nlm.nih.gov/bioproject?term=PRJNA674939. BioSample accessions: SAMN16680812, SAMN16680813, SAMN16680814, SAMN16680815, SAMN16680816, and SAMN16680817.

## Author Contributions

XBS and DYZ designed the experiment. JD and XYS performed the experiment. XT, JBC, ZHZ, GC, ZZ, XGZ, and YL contributed reagents and materials. XBS, JD, and XYS wrote the manuscript. All authors contributed to the article and approved the submitted version.

## Conflict of Interest

The authors declare that the research was conducted in the absence of any commercial or financial relationships that could be construed as a potential conflict of interest.

## Footnotes

1 https://www.arabidopsis.org

2 http://www.genome.jp/kegg/

3 https://biodb.swu.edu.cn/qprimerdb
