# Supplementary material for: NSs, the Silencing Suppressor of Tomato Spotted Wilt Orthotospovirus, Interferes With JA-Regulated Host Terpenoids Expression to Attract Frankliniella occidentalis
Source: Front Microbiol. 2020 Dec 10;11:590451. doi: 10.3389/fmicb.2020.590451 (PMC7758462; doi:10.3389/fmicb.2020.590451)

Fig. S1. Molecular and protein identification of transgenic *Arabidopsis* based on NSs transgenes. a. RT-PCR. M: 1 Kb DNA markers, 1-13: T1 plants, 14: Wild type *Arabidopsis* as negative control, 15: TSWV-infected *N. benthamiana* as positive control. b. Western blot. 1-6: T1 plants, 7: Wild type *Arabidopsis* as negative control, 8: loading control.

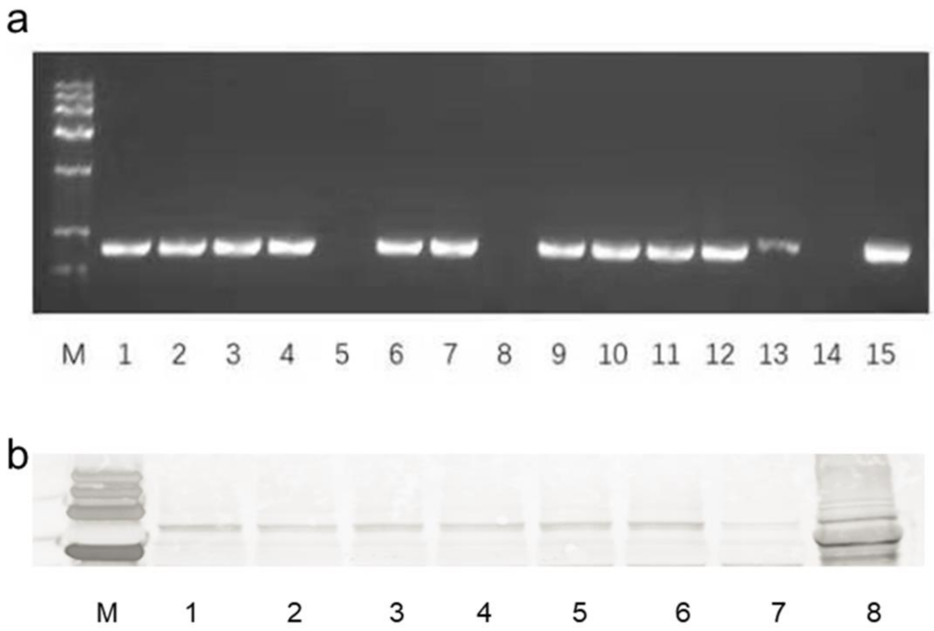

Supplement: Supplementary file 1 [file Data_Sheet_1.pdf]
