# Supplementary material for: NSs, the Silencing Suppressor of Tomato Spotted Wilt Orthotospovirus, Interferes With JA-Regulated Host Terpenoids Expression to Attract Frankliniella occidentalis
Source: Front Microbiol. 2020 Dec 10;11:590451. doi: 10.3389/fmicb.2020.590451 (PMC7758462; doi:10.3389/fmicb.2020.590451)
Supplement: Supplementary file 2 [file Data_Sheet_2.PDF]

Additional file1: Table S1 primers used in this study

Gene ID	Gene symbol	Primer name	Primer Sequence(5′-3′)[a]
836390	β-TUBULIN-2	F	GAGCCTTACAACGCTACTCTGTCTGTC
		R	ACACCAGACATAGTAGCAGAAATCAAG
818339	ACTIN1	F	TGTACGACCACTGGCATAAAG
		R	CCAAGGCCAACAGAGAGAAA
816244	HMG2	F	CAGCTTGTTTGAATCTACTCGG
		R	ACTATTCTTGCCAATTGCTGTG
824592	AT3G54250	F	ATTTGGTGGATTTGTCAAGTGG
		R	AACTTGTCTCAACACTTTCACG
829932	SQE3	F	ATCCTCCGTGATCTATTGAACC
		R	TGCGAGTGTATTGATAGTCGAA
821519	DWF1	F	AAAGGTCTCTCTTCTTAAGGCC
		R	TGTCTCCTTGTCTGTTTTCGTA
842213	XF1	F	AGCTTCTCCTTATCCTACTCCA
		R	CTAAGGAGATTACGCAGGACAA
829617	SQS2	F	AATATTTCAGTCTTGCGCCATC
		R	AGAAAAATCATAGAACGCACCG
818710	CAM2	F	ATGGCTAGGAAAATGAAGGACA
		R	ACCATCAACATCAGCTTCCTTA
827114	CAM8	F	GATCACTGAGTTCAAAGAAGCC
		R	TTCTTGTTCTGTCGGATTCTGA
815949	PR1	F	TGGTCACTACACTCAAGTTGTT
		R	GCTTCTCGTTCACATAATTCCC
