# Supplementary material for: NSs, the Silencing Suppressor of Tomato Spotted Wilt Orthotospovirus, Interferes With JA-Regulated Host Terpenoids Expression to Attract Frankliniella occidentalis
Source: Front Microbiol. 2020 Dec 10;11:590451. doi: 10.3389/fmicb.2020.590451 (PMC7758462; doi:10.3389/fmicb.2020.590451)
Supplement: Supplementary file 3 [file Data_Sheet_3.PDF]

Additional file2: Table S2 Summary of the sequence assembly after RNA-seq

| Sample | Raw Reads Number | Clean Reads Number | Total Mapped Reads (%) | Unique Match(%) | Number of expressed |
|---|---|---|---|---|---|
| WT | 56821971 | 55536561 | 0.935 | 0.9158 | 16439 |
| NSs | 49431901 | 48357159 | 0.9058 | 0.8874 | 16140 |
