# Supplementary material for: NSs, the Silencing Suppressor of Tomato Spotted Wilt Orthotospovirus, Interferes With JA-Regulated Host Terpenoids Expression to Attract Frankliniella occidentalis
Source: Front Microbiol. 2020 Dec 10;11:590451. doi: 10.3389/fmicb.2020.590451 (PMC7758462; doi:10.3389/fmicb.2020.590451)
Supplement: Supplementary file 4 [file Data_Sheet_4.pdf]

Table S3 List of DEGs in NSs and WT.

Gene ID	WT1 expression level	WT2 expression level	WT3 expression level	NSs1 expression level	NSs2 expression level	NSs3 expression level
10723040	6.86410893	9.66376374	8.03299218	4.12668296	3.36218398	4.838615129
2745823	9.83611848	6.83491973	9.91754756	2.11745817	1.93103742	3.378276615
2745854	7.3939947	6.48810513	9.0172933	20.5770796	14.2352117	18.63381571
2745871	12.7904297	8.98653861	14.8602834	28.4594341	21.2336391	31.85390518
2745895	1.44787383	1.56654622	0.84982729	0.36966625	0.07031663	0.300706459
2746139	8.2879163	7.00646656	7.35871791	25.0669681	18.7151166	19.00758294
28716296	5.40543819	2.54336518	6.63337909	1.61906709	1.87527439	0.590395656
28717232	1.91921654	3.12606865	1.65932934	0.11526173	0.5462901	0.618674627
28718257	5.15584735	3.93800881	5.01065545	21.2587089	8.70901159	18.35672891
28718327	1.51238189	1.5131664	0.79103403	0	0	0.370194033
28718845	12.7685113	13.6527723	11.7711905	4.30187717	6.41799639	6.454674121
28719339	12.6244824	20.240455	12.0546113	2.31295298	2.09715173	2.668281667
28719390	0.24889801	0.20910236	0.70550926	2.82773329	1.78331224	1.039945675
28719419	35.1313939	25.3284008	42.3757291	6.95721198	13.5545861	7.423052831
28719424	1.97255003	2.56625623	1.52042905	0.0672859	0	0.711541778
28719430	31.603994	32.4257931	28.4157856	8.7879004	15.2311075	15.46008392
28720126	2.30596689	2.83829645	6.39511866	0.22141088	0.22812839	0.159640784
28720145	2.41661789	1.55923736	3.24377338	0.02486091	0.69673301	0.215101401
28720216	0.02771851	0.0304518	0.26189664	1.4012453	2.07308909	0.621735534
28720553	1.06635942	3.029772	2.60571497	0.40513914	0.50091706	0.538402127
28721262	4.24518959	4.19742236	5.30691606	0.33862841	0.87225562	0.569698482
28721286	1.23157149	1.0455118	2.24794617	5.07942616	4.96438056	3.487949892
3768391	137.711528	183.812745	96.3728764	8.06316854	18.4303569	19.87369171
3768908	16.4404894	19.3444225	21.4536384	4.81074915	4.99012117	5.391311593
3770664	3.9149222	3.6766339	2.98305653	0.49109144	1.18064552	1.613052846
5007719	44.6182761	55.654173	48.5527461	5.16435547	17.9885473	7.393780854
5007997	7.34909773	10.8748919	8.32541415	3.45076963	4.32664972	3.037068564
5008204	69.7567224	53.8405279	73.2458181	15.0700427	41.6629286	18.77050232
5008297	11.1058963	11.8646659	10.4407394	1.51533856	2.61705842	1.075240709
6240283	22.4147352	24.3994526	33.1094757	7.85136642	17.5868241	4.434952178
6240858	7.9997123	7.63134252	9.30688141	1.95579614	1.61210731	4.25708756
6241030	0.30207144	0.42477867	0.22832827	0.01566208	0.01290981	0
6241268	0.71741192	1.46371652	2.34823915	4.54999366	3.23029804	5.459714796
814644	10.0234598	5.573905	7.89217015	2.86051894	4.25293477	4.060707979
814646	38.8224596	28.6800139	53.1433251	10.2328045	21.3032751	14.43100827
814707	5.18336155	5.84674559	4.68794988	2.4791263	2.9171325	2.797809904
814722	19.2130293	20.3794787	16.6462708	7.1937558	8.1473761	11.6351602
814807	1.8434544	1.89963924	1.25569936	0.21301991	0.19848883	0.329441045
814812	2.62751606	3.11538805	3.00920284	1.27024571	1.96972053	1.030350874
814838	1.33791106	1.07175896	0.61889062	0.03612981	0.22335583	0.104200679
814931	1.83754169	2.20652918	0.64602416	0.03692812	0.1674134	0.426012184
814990	3.34315471	1.86684731	5.07844697	0.50277869	1.38142073	0.25318261
815021	3.52337523	6.94842306	2.30576906	0.01871754	0.20056837	0.107965154
815055	2045.73089	2538.72217	1807.93637	262.56367	1102.20143	369.1146318
815058	1863.68463	2164.5866	1531.71353	281.717673	1112.44125	332.0659647
815093	11.5765232	5.17179836	7.13348252	0	0.22143663	0
815130	21.1478844	29.8806034	20.9777273	1.78831868	7.18077988	2.861254043
815145	35.43284	31.4679703	30.8451496	14.7417503	28.0987837	13.43135042
815247	51.5896306	61.1876464	36.5779015	1.44822213	2.6709674	5.360181947
815261	30.2618669	32.5287475	22.7229695	11.3010541	8.86513185	18.92071314
815415	3.55167753	4.43593962	3.77062324	0.64025878	0.56125491	1.133330568
815558	265.788548	402.149209	142.867583	1.23822992	7.89651367	6.03020469

815846	1.67119322	2.20699024	1.11267492	0.71833826	0.3423112	0.755319478
815865	1.39592873	2.96492053	1.40686363	0	0.34800867	0.463868912
815949	26.7427395	7.74049644	26.3981652	3.1304357	3.24316631	0.193268
815978	10.0001921	13.9687758	9.88337224	1.57060691	2.91696203	2.06418106
815979	3.38208477	1.93456239	1.98070782	0	0.17918448	0.174153582
816142	2.23916227	2.87378897	2.00361443	0.37976024	0.56642727	0.469394052
816176	1.19859274	2.71761914	1.20476911	0.02203749	0.20889613	0.148300924
816186	0.97290287	1.85562439	0.90647403	0.03503053	0.23099735	0.117868652
816217	15.3815943	12.1437998	14.5462271	4.69864435	7.083245	7.866217035
816244	1.89035855	1.62277414	1.06334876	0.33050908	0.48849385	0.536865002
816254	8.48536139	8.52305614	8.37621285	4.1481638	6.00952731	3.77637923
816262	11.3182161	10.1650667	10.8423152	5.76190209	5.36885592	9.61086726
816287	2.65971301	3.44303722	1.97702106	0.48217912	0.76508467	0.254949823
816353	1.71627307	2.11596424	1.72971736	0.09887433	0.24449791	0.118816614
816434	2.28021756	3.34251682	1.61583713	0.30931431	0.99858948	0.553421383
816506	5.4783175	3.76157987	4.19711212	10.324655	8.63547221	7.040028998
816512	3.1509087	1.89630359	3.14910148	7.20850975	6.31732229	6.171239384
816598	0.54951647	0.82612075	0.27326697	0	0	0
816604	4.96598122	7.49600277	0.86466455	0	0.014379	0
816632	0.44742641	1.03770947	0.4932061	0.14499108	0.06639558	0.015487539
816640	2.45299985	3.40652409	2.03676823	0.15141116	1.37284321	0.635170131
816648	12.2786355	12.90204	15.8517533	34.6516203	21.8255737	31.7962027
816649	0.37537653	0.70931416	0.29792465	0	0	0.018710715
816659	0.72497145	0.50442468	0.2511611	0	0	0
816693	2.63528767	4.27575566	2.47221335	0.69290905	0.38076352	0.888175096
816719	4.08828256	4.08406155	3.29684513	1.14613312	1.86913341	1.552054734
816832	7.13477664	8.61092346	7.2486556	1.82313376	4.93177681	2.071346826
816845	3.85033004	5.55943705	1.2646244	0.16636286	0.35261534	0.114238359
816861	9.75039919	13.4868968	12.9842077	4.97065259	4.7404094	8.480720601
816870	2.89348115	3.1029374	2.70778865	1.54857557	1.70438869	1.299411989
816886	0.10119011	0.06948021	0.01195111	0.95094573	1.29738638	0.252192023
816899	20.7303031	23.7102025	22.1592064	11.9668033	14.9664726	10.51879683
816900	2.6335435	3.28310247	1.58826851	0.02421035	0.25942676	0
816990	19.2035083	16.8041198	14.482299	4.09353913	10.5315573	6.022267003
816991	185.681095	166.835003	297.067555	107.135714	89.8739782	138.9597718
816996	34.9285752	43.9015518	55.0062655	6.5349302	24.6505127	9.316427899
817048	8.62068207	8.20442239	7.07144334	4.04043387	4.57932075	4.895823606
817060	1.38933478	1.59156341	0.62830296	0.076961	0.07612413	0.429123532
817065	2.32091185	2.30748794	1.16094666	0.47644644	0.9537517	0.549640785
817089	849.032966	1224.56189	670.949075	234.823556	253.50146	249.7871346
817091	1.59071829	1.22620051	0.82207214	0.30758003	0.53522894	0.273790262
817155	76.3807187	61.8398576	99.4081701	12.1861144	23.7913346	2.225146921
817159	54.5557341	54.2225357	96.8447004	21.6423619	35.8622798	25.50228952
817165	115.629668	107.393303	109.36921	48.5016496	54.2863429	63.98827368
817171	1.24027479	0.89901974	1.24435254	4.42524286	3.10114497	2.756484623
817173	7.26181861	6.44622958	5.07962502	1.62516144	3.45610222	2.387280695
817247	40.7228093	46.0856999	37.6855856	21.8159526	20.7293117	24.967197
817248	3.21071237	2.98742142	2.79629763	1.35895467	1.93692225	1.7215034
817336	14.7359311	12.4336201	9.06033192	5.22748118	8.20736102	5.96674047
817352	5.77583914	8.28785331	6.8038641	1.80571557	3.74389885	1.856113003
817355	8.08650015	7.83071816	6.72404847	3.53782605	5.00080096	4.075704879
817360	1.29794639	0.63984277	1.28924673	0.08627835	0.08889599	0.373248393
817361	8.61019865	8.48417613	6.6412523	3.13399816	5.00303383	4.182441844
817395	13.2297136	12.7440735	9.56820275	2.03938722	5.16976995	2.520738445
817421	52.1088622	36.0153225	48.6876845	126.445743	85.923226	105.7112243

817444	42.5844888	54.7789002	29.3335768	9.17267693	17.350843	10.22785367
817453	3.65665873	3.98633562	2.60451694	0.80212754	1.02180971	1.156693531
817479	23.3389041	22.3242055	33.9255687	11.0985211	12.035991	19.37248
817483	63.0710873	53.0302133	69.9093131	26.8625862	33.1485273	34.209475
817504	13.2940176	17.8209098	11.5209717	2.75312729	4.23293741	3.442122533
817513	321.578174	272.501774	279.629417	670.932708	449.534133	649.0741404
817517	50.6214169	76.8311088	79.4344433	162.129838	121.577548	129.7633081
817547	14.2719358	11.8383582	12.0712332	26.9308778	22.731166	18.67628304
817548	61.3692345	107.936491	47.165486	4.7852202	13.4240631	12.5377493
817552	25.6741229	35.5829485	15.8111542	2.31418656	1.77940147	9.629532061
817558	8.3326479	10.4052212	9.02571595	4.47564531	6.41453491	6.053238322
817567	8.39165385	9.70137691	6.66019401	4.01629045	4.11055532	4.697646622
817593	2.74942206	4.48209052	3.1285026	0.34489003	1.5161764	1.252566148
817599	45.8322451	37.2604745	53.4573039	25.5941628	35.4786854	20.13359212
817601	129.423897	150.401931	133.406178	67.8545794	96.8059997	67.2521745
817610	20.8443103	23.6942717	32.576292	54.3904443	37.0977472	48.29102242
817652	1.36429283	1.49882411	1.06259005	0.46998749	0.42679371	0.589669462
817667	9.70971593	11.8063927	12.1728406	25.1922766	19.5398425	21.04698066
817730	6.00643471	4.72708449	4.581388	1.30233156	2.19362279	1.034262912
817732	30.6437378	27.6494707	37.5114159	19.7734138	25.9106973	13.87615265
817744	13.6535549	13.0365758	15.3126831	5.67873096	8.3796443	9.836874196
817746	91.9753263	68.3957363	145.61643	28.9008137	53.9660702	26.83987484
817826	0.15329668	0.10636616	0.32017578	2.175315	2.16372919	0.683677607
817838	0.18555204	0.51561833	0.36094821	0.05659219	0.02332367	0.01360129
817841	23.4941509	19.8953843	25.451563	11.4901535	9.59624426	15.02636431
817844	0.48821675	0.75789889	1.09305338	2.77898291	2.76690785	1.520953615
817896	7.42173982	9.9709548	4.6890045	1.82552531	1.70774771	1.824619157
817918	25.026832	29.7577902	21.4527802	6.56478227	14.2635849	6.637274671
817922	3.23775206	4.75548074	2.41600223	0.57692522	1.01635682	0.554629726
817943	49.1681912	38.1018448	48.6719104	99.6749807	103.184832	66.21376739
817967	2.2451789	2.56393802	1.6328762	0.30634233	0.69440057	0.404942475
817978	10.1692997	11.233411	7.45464161	1.15776864	2.62436864	2.97966401
817991	4.56439921	4.16588327	3.0520253	0.48242055	1.11790928	0.691290589
817999	1.41785802	0.89009788	1.49275777	6.33624093	3.04421926	4.778868428
818020	23.9186949	26.9878775	20.4417594	7.04946515	14.9356894	10.37939245
818027	29.2638179	48.1604068	24.9835692	4.10952576	7.87429486	5.398616397
818030	0.86684424	0.82674169	1.38605489	0.18521263	0.30533104	0.273017698
818066	20.0440283	15.1735492	18.1582745	10.6907804	9.02216936	12.71535307
818086	4.27493921	5.03287323	2.37007972	0.58007825	0.27681908	0.176103445
818134	6.73244355	8.52909169	5.47284497	2.20134159	2.30053118	2.431191052
818140	1.43140573	1.88466506	2.2506501	0.19828924	1.30755362	0.204242446
818159	7.84063962	12.8012321	5.32091176	0.08902087	0.80715118	0.385112818
818178	3.9001959	6.50315574	4.58674359	0.68124068	0.11821629	1.361531499
818192	0.49359965	0.60532791	0.43383683	0.07439711	0.01839703	0.042913203
818211	10.1877892	7.79736836	13.0590966	23.6381044	20.5001562	20.07974525
818212	75.4023457	59.6747974	68.1963146	153.978424	95.8704155	121.7102245
818216	5.51957455	8.07303404	6.984743	1.59943121	2.26005566	4.489303004
818224	7.75471139	7.80527515	4.17399875	1.52951024	2.68513782	2.615767319
818230	1.6602724	2.83354704	1.89703031	0.33031971	0.42078582	0.182834344
818235	14.338445	16.5121739	14.444912	4.36211463	10.2683229	5.492764396
818259	2.93156043	3.63620428	1.66787678	0.02042987	0.85882784	0.15712279
818260	74.5679847	72.5762943	48.6548	31.3360624	43.8217215	26.09122752

818263	30.4541244	33.8726828	23.8571583	13.6290886	16.6813836	19.02202717
818277	1.03904555	1.72440054	1.04439977	0.08596816	0.04724075	0.041322946
818280	19.9918649	21.6785855	27.2256883	10.3496557	17.1638479	4.656216752
818284	8.67686788	8.04275574	6.05529151	4.55117404	4.95875206	3.87238508
818289	54.7160548	40.6617862	75.2528949	171.454197	122.236233	191.5661189
818314	0.63002712	0.98189194	0.34609142	0.09495995	0.09392735	0.073032147
818335	4.99287889	5.87171318	3.88649651	1.94682678	2.05287752	2.343703961
818341	15.4625682	17.6655442	8.32902814	1.93543652	2.73046087	3.73917249
818383	14.7573503	13.4606083	16.4816718	30.4225794	27.9079968	28.99807409
818388	0.12916305	0.10319975	0.44377786	1.79600391	0.90329193	1.00962009
818390	30.3146668	32.3163918	30.9479895	13.9200756	22.7077353	17.11272264
818436	366.528633	377.285247	372.57114	153.157519	162.763905	83.18282576
818440	5.75413757	5.83140639	5.18816197	2.4911576	3.05564075	2.508928078
818463	3.79604233	5.85691036	3.21745145	1.84519378	1.72969751	1.147808315
818487	217.060113	187.902307	176.655385	64.2882057	80.5896664	108.6569812
818530	17.7199172	18.1322852	12.8188666	8.8542134	11.8738809	8.394428827
818541	0.4261853	0.61870729	0.47458544	0	0.01626262	0.018967222
818577	7.26273616	9.28120999	6.51510013	3.11639981	2.40821251	5.201328786
818579	12.7487717	8.04846818	9.14447537	45.4327972	19.6261746	32.53401714
818604	6.0133123	7.55601751	3.65162554	12.5487999	9.44712763	13.51340953
818606	2.47082882	5.20399212	2.1668727	0.07077889	0.61258083	0.187119935
818627	0.56661679	0.40202491	0.73612602	2.15748101	1.21079178	2.250623875
818647	20.51605	17.8721908	20.4335049	7.37105712	13.5956587	5.558275628
818650	2.2186423	2.67452305	2.41438158	0.92877801	1.06072312	0.860612086
818709	157.776682	184.453647	277.387323	57.6961519	88.1249147	84.83012037
818710	26.4842485	22.3637524	30.7475174	11.3428719	22.4688185	13.33417722
818716	4.26948388	3.60625808	2.36161044	0.77868176	0.56161463	0.561441983
818731	2.30435545	2.63898575	1.95293269	0.65169996	1.01466918	0.504692456
818839	9.64520472	13.8364997	6.93981331	0.16236798	0.26767065	1.475790354
818854	107.625098	87.0626188	84.9928012	38.2688584	70.5983309	27.14100645
818861	22.5917916	17.7396078	13.3752797	5.6315377	6.88551002	11.84291682
818887	1.44530261	2.55511611	1.72660205	0.21533727	0.10649784	0.186313932
818917	12.1524945	12.8684645	14.4397483	4.20429611	6.82503241	10.48402095
819011	115.285514	112.194204	100.645906	45.8743864	64.0260509	55.85440997
819022	44.6119063	50.2699142	37.6645675	22.1414111	28.9476973	28.49797427
819029	54.0942954	68.9209268	50.3166699	32.370868	29.9399884	31.09303316
819038	3.87350618	3.22757715	5.1980598	0.82469335	2.27923334	1.189234151
819066	1.03082122	1.47221004	0.52350623	0.01670217	0.02753426	0.337190978
819082	5.07746688	7.47509633	4.21590863	0.70912091	0.31052002	2.535133879
819127	19.0802908	22.7951023	19.8895744	8.39178434	16.7557321	12.07616679
819146	3.8438916	5.4845563	4.85254972	0.53753544	0.40899252	2.166426084
819191	1.86714753	1.64925805	2.41131803	5.37560678	3.89964603	3.881744347
819241	41.6709478	50.7369821	50.4209543	17.3925315	25.5426139	24.99227415
819265	6.57934673	7.72151578	5.34657579	1.22248661	2.49516071	2.070666522
819281	31.9849961	22.8133941	38.3555816	89.5112804	73.9722609	69.90342076
819283	20.4203196	25.8390212	19.7549138	6.43978918	9.06071493	9.183511419
819291	1.48752334	1.30985995	1.33037542	0.14718792	0.59448185	0.282999571
819307	9.5304412	9.79759379	7.69499262	2.87501393	3.77685675	3.498070995
819315	5.43762746	5.15367123	3.9557498	0.81075949	1.8755125	0.389713695
819320	50.5360103	44.491886	44.6806477	13.8795074	28.525492	18.57761721
819326	10.9901401	9.88188319	15.1432839	8.62795816	5.81873016	4.870742203

819329	7.70615676	6.35521299	4.58729746	1.29436101	2.81810101	2.780456155
819357	13.8309779	17.1138706	11.1468305	4.80712702	7.30264447	7.568304075
819363	2.19268249	2.51676148	1.42754398	0.41006939	0.95574839	0.591333164
819386	227.100716	242.092333	358.945445	119.840672	170.822541	99.33025301
819401	13.3003929	11.1419111	17.6021937	37.1306052	24.7889032	36.28249669
819597	2.2840053	1.82815206	1.8034951	7.84553651	4.14854742	6.993439189
819621	6.27222994	8.00540341	5.47599687	1.19561877	2.36523518	1.551708416
819641	8.26967437	9.29749285	7.14654039	3.79098635	4.31007251	6.534933739
819708	1.09807947	1.30976203	0.93672658	0.26840421	0.63689723	0.516063268
819730	3.82668218	2.35774791	2.83777659	7.13125458	6.73948305	4.825142682
819738	14.176244	13.1177363	12.719175	23.2906629	30.4776375	24.67416788
819743	0.86560648	0.7524102	0.68305037	0.16028841	0.05081584	0.082973696
819751	0.01786772	0.01962964	0	0.19686466	0.35317579	0.311717006
819759	29.40072	37.6252828	30.433926	16.1243404	22.5457124	21.94748627
819760	43.9510866	50.4341246	37.6033656	13.5382553	18.431625	18.1184522
819787	1.57574404	2.68817168	1.28305851	0.39853959	0.57488355	0.351209724
819811	1.30094351	1.30647221	1.24427052	0.37243716	0.81863837	0.268533139
819828	47.2029882	34.8243913	48.8119733	69.5609877	86.8062577	81.24755903
819834	21.9375104	22.1149831	19.059975	11.7589582	14.557083	11.54889375
819862	6.14087769	7.30047624	3.39160686	1.33306662	2.58854048	1.675872767
819873	2.27411205	4.09196329	1.29097691	0.08491478	0.28997036	0.122449826
819874	3.23039351	2.41010018	3.71277189	0.49997729	1.85452698	0.300409677
819886	10.8833295	14.3903758	8.32158306	3.00217711	3.11188349	3.648720047
819895	54.4701644	56.9640103	64.9688466	21.280046	36.4194306	20.33941834
819907	7.97227598	8.47973576	7.54967525	0.46972028	4.66548417	1.625643953
819920	2.20924863	2.32643685	1.77957634	0.14516343	0.42422816	0.596274185
819922	6.28391039	6.77196646	6.18107467	1.38542578	2.21502257	2.0896824
819923	59.6985336	43.99441	66.7884503	13.4612466	21.9172803	31.90114799
819935	32.3917838	35.1603997	26.5065963	18.8598306	17.2008808	22.81254421
819943	3.1197156	3.04982066	2.788484	1.27516545	2.31094216	0.730493835
819971	9.13075852	9.0338001	10.0940035	5.13326339	4.35771787	7.672846822
819987	0.21296087	0.35825229	0.05030369	0	0.0071105	0
819990	5.44141828	3.94887549	4.13811187	1.96402604	1.98520938	3.211191588
819994	55.3736849	43.3401563	56.461054	9.6782895	31.5264958	14.08755732
820010	3.73725669	4.16402113	2.93008156	1.44298817	1.68500351	2.113864168
820015	10.7572006	12.3049588	9.09583011	5.84180953	5.97563139	5.33064478
820091	28.0260014	23.8012247	38.9908903	12.0879669	19.4260491	5.925850097
820102	274.256552	323.503102	297.052521	119.516375	212.707807	68.94398822
820109	1.33963844	0.9533904	0.80402931	0.1965815	0.30606905	0.220481959
820145	1.30014242	0.8161988	3.65019661	0	0.15875701	0
820209	18.296839	14.7721301	27.5321921	48.6509915	43.9250703	32.19292678
820241	31.5441154	35.428593	35.4620807	3.37631059	10.6863127	5.371216343
820272	12.9109411	12.463861	12.3243118	7.7017532	10.276837	5.104484738
820342	36.3356115	49.3599983	28.2806532	5.62946346	15.6508681	11.90161731
820385	2.50371946	3.99083782	1.34122426	0.24630103	0.26273042	0.097498734
820394	0.57205581	0.72983095	0.41845425	0	0	0.045990697
820402	1.87915303	2.30158728	1.12768634	0.11520681	0.42054469	0.110754597
820441	10.6555059	11.1550422	5.45150461	0.48001033	0.67644909	2.307300744
820453	1.97899344	2.18945044	1.86983974	0	0	0.382069156
820486	7.8348249	10.2191532	5.9870329	2.33661537	3.84367392	4.123108155

820513	10.6428679	9.8879092	7.13148909	1.72290274	2.85042363	3.856860659
820531	21.2548145	19.8936057	34.5835962	56.6524395	49.4103412	67.78647546
820554	41.6478498	62.1537402	25.1769971	2.84635992	7.38122089	2.428904958
820576	0.25954606	0.43521304	0.65825027	1.75297489	1.24059432	1.77034318
820616	9.10483206	8.57934681	9.99674886	22.4997846	14.6368053	18.8050327
820639	725.78231	675.919809	517.309955	56.3486921	212.143522	99.19086662
820640	17.4117444	18.329746	13.7028758	10.5143055	8.74135639	11.67645535
820644	14.9099053	16.6972835	11.5059807	2.10582054	4.54742571	4.057246114
820655	17.3335941	14.7904762	14.2054336	5.15054932	6.84294632	6.077262374
820698	1.33657954	1.67814632	0.96217823	8.11252299	4.94152254	4.943780187
820702	0.92330319	1.34155836	0.84423612	0.01930333	0	0.074229396
820719	0.83757992	0.72757839	0.82819069	3.15607619	1.87305417	1.626282134
820787	29.3646948	32.9043473	27.5268502	14.2996005	19.9862373	17.04537859
820792	38.8688504	36.872458	51.1619599	19.3193127	21.2458529	19.83584218
820793	5.34253715	7.0867067	6.11352331	1.2311322	1.96615053	3.13148832
820797	11.5585518	12.6983267	10.1880716	6.74964794	6.1126455	8.035485144
820819	56.195576	36.1943762	80.5601589	18.7747661	32.6211857	18.69030037
820820	30.5009295	29.6041568	34.522539	16.3760452	19.5842529	20.53591039
820850	24.0544653	24.0717727	29.5145559	9.61115767	16.4323857	5.847017219
820863	4.26677988	4.98994267	4.47359866	1.07045758	1.6176377	1.715148914
820870	151.442614	320.731188	176.043112	21.2643832	22.4303357	50.45722877
820884	78.4865375	116.155212	63.209282	14.9791241	23.8445383	36.48932195
820907	112.631438	92.4295103	93.7963197	44.8221297	48.3686894	75.7749986
820933	27.8861112	20.3814015	29.5683338	8.0325809	19.1182204	12.16240016
820938	71.0886037	68.1525999	61.8828661	37.9014749	37.0244022	52.55886665
820947	1.59024088	2.57018317	1.51697668	0.31712408	0.31040821	0.323922998
821003	150.682775	140.247676	100.733952	83.1343723	79.6232349	69.26735517
821018	2.03056621	2.52432394	1.44734074	0.36941281	0.09515516	0.399528806
821028	10.5415629	10.8270789	12.0740647	5.76459051	8.60858786	3.882691913
821053	7.28818119	8.4396632	4.92424841	0.90428388	2.6395064	1.196617487
821072	0.94884493	0.78180704	0.46226311	0.01921741	0.22176517	0
821114	13.0225141	21.6582828	8.44241565	0.25069426	2.97561845	0.337408521
821136	22.8698082	23.8092293	21.1065629	8.73401901	12.5179363	16.13112301
821140	3.78504666	3.7147351	2.69412943	0.73593959	0.68749602	1.729441821
821175	12.0303427	5.74448744	13.6327248	46.0685633	37.5669571	27.50050649
821191	0.56007915	0.43950563	0.17639578	0.09218887	0	0.011078275
821223	6.14924504	6.97086712	4.63524466	2.31504615	2.72143753	3.267940745
821270	9.61752784	3.76430407	19.3690585	36.6750266	23.5525192	40.26048934
821277	70.8999052	81.0164444	71.5448866	41.0918836	37.6572006	62.96586831
821279	21.3852726	27.2953042	15.4659217	7.08752008	9.29781055	9.510227614
821330	56.7309506	69.0578568	54.8808927	27.5480676	28.2293715	37.21792877
821379	234.074289	209.659966	233.752147	122.394129	197.336833	124.982878
821402	4.86150238	6.77108557	7.22637475	0.43504703	1.53218691	0.392886936
821404	0.23692175	0.46953249	0.40820445	0.04817308	0.04467117	0.052100339
821429	0.89485282	1.57890723	1.35791809	0.14059741	0.26075354	0.236536955
821445	15.1166891	12.6728576	12.0622993	6.88038749	10.7959846	7.341688065
821464	0.59448082	0.67231074	0.56995171	0.02266411	0.2802207	0.130729461
821471	5.31144197	6.67933349	5.29337202	1.73087277	4.28012805	1.524542667
821490	10.2389142	10.7472884	9.13959485	6.30475074	5.66483425	6.709286628
821494	3.93455601	5.81674845	4.58300026	2.04182349	2.56530378	2.023446931
821499	0.56477725	0.95729542	0.83093238	0.13595719	0.12930653	0.261406144
821519	54.4306104	79.7865347	52.5349316	19.3160547	35.9688439	26.39502748
821611	1.29722361	1.68230961	1.38233879	3.76755094	3.97043607	2.455149034
821614	15.4703522	22.9477243	14.2189366	3.86235281	4.79152081	7.313663606
821630	57.2711449	53.7542046	53.6955818	34.6272103	29.2557835	46.0079504

821660	1.39240077	1.22376299	0.66553947	5.81802468	8.34860163	4.878727027
821672	4.66497309	6.80569	3.31847752	0.20947499	1.47340197	0.698116461
821732	13.8015543	9.55129373	16.9664294	7.93503176	6.83131602	9.231109761
821742	13.7204182	18.0298856	14.1099562	6.23322958	8.6495409	5.689978125
821753	1.43980938	2.48759704	0.63950193	0.0159514	0	0
821775	169.618016	198.477662	109.974408	8.2509946	10.9347653	11.51543577
821788	204.581723	441.131589	192.125242	14.2762759	13.5630445	37.59757299
821791	396.113008	734.707103	399.705951	76.7155278	52.1281033	119.0041685
821828	4.04869474	0.56782106	4.02886215	11.5567892	9.01972467	6.97740321
821850	0.64930774	0.68968872	0.33894719	0.03719989	0.09965415	0.058113715
821879	163.04967	170.457056	153.794821	47.083817	43.3467128	126.4944902
821882	54.485694	67.0292466	61.3689305	34.2574752	36.0440319	40.58166547
821884	2.16054556	3.52916002	1.92050707	0.73698644	0.34929929	0.921056925
821886	5.45843572	5.12125695	5.2477174	1.68362319	3.49920575	1.171717521
821887	4.13368766	2.32558796	2.79540262	0.89663098	1.94116523	0.508879962
821908	4.16070932	4.4810707	3.36409364	1.25546772	1.63243384	0.985958914
821931	0.22546589	0.48163658	0.23078257	0.01623634	0.0267663	0
821932	10.6337077	19.5691136	8.36528413	0.32637508	2.47750242	0.897505817
821956	0.680342	0.97581101	0.52675278	0.02449652	0.01009589	0.11774923
821961	7.43184715	5.89312964	10.8109989	29.4245771	16.3841207	25.75671204
821963	8.51498691	7.98390827	3.82618658	0.56747149	1.16937665	1.472961621
821965	64.1843718	75.8525044	51.9765979	22.3666736	30.8256143	28.794308
821970	1.53200287	0.95525698	1.13452974	3.84640247	3.19997335	2.235852962
821972	0.32057232	0.45026	0.22237551	0.02629957	0.10838994	0.020226568
822038	21.6841318	29.8269225	18.5684096	7.62366904	10.397121	7.728815864
822101	0.34442131	0.66217249	0.26847509	0.01116117	0	0
822104	0.94577121	1.00192426	0.68084274	0.05837763	0.25262488	0.098212809
822136	8.98229008	9.01753925	6.02795412	2.03539862	2.1998095	3.256671984
822143	6.32339358	4.78376101	5.29438156	3.05823783	3.8924407	1.869958969
822155	1.56944715	1.5257384	1.44020958	0.01463563	0.47048581	0.393960984
822160	19.4941856	19.2594279	29.1523125	41.4935649	47.1635041	47.14276359
822186	0.73474435	0.58118156	0.72198742	0.20952633	0.0314012	0.036623474
822281	1.77644857	1.7369436	2.09808309	0.16118712	0.60736903	0.287779119
822282	2.40401148	3.11945032	1.8342751	0.6937809	0.88202742	1.051324779
822323	38.7882949	48.0847105	34.1364987	14.4160229	25.5918141	18.88177918
822391	435.379846	652.658757	313.45648	16.2938588	157.278568	23.09001657
822420	3.71281825	2.94589735	2.56967058	1.09918316	1.28965794	1.808774015
822421	0.49962616	0.74262088	0.23603435	0	0.12560481	0
822422	0.29429118	0.51435821	0.93529005	2.61825039	2.42970481	2.400381901
822437	18.2391408	16.9633318	17.0810482	7.5275585	14.1822927	8.887659382
822444	25.9218032	32.5018839	24.6867234	0.6231461	3.02477866	1.98737232
822462	1.20668768	1.56556223	3.36610226	0.22344802	0.50343043	0.114566819
822463	0.3266923	0.54096133	0.27735853	1.41769058	1.1432686	0.731601828
822472	0.51526823	0.99063687	0.38252344	0	0.09830947	0
822519	28.9328862	23.1515835	24.4216291	7.06067198	13.5567969	9.724787466
822543	36.538883	51.2505927	15.601093	2.63226044	5.88062881	5.078523482
822579	149.12453	139.723939	170.770851	96.5323777	97.6446554	111.5489975
823286	2.14374886	2.50857076	1.73520709	0.87798467	0.63416824	0.687425932
823481	35.4906858	45.9051783	25.4591531	7.41421066	7.6330187	11.67909953
823490	3.01823786	2.15531073	3.44248585	7.51722195	5.64340918	5.507348713
823509	0.2079369	0.0761471	0.15280836	0.97331201	1.22192424	0.719767694
823560	2.91497176	3.02307845	5.662621	0.60455247	0.74747319	0.34871359
823603	9.50315792	6.59019285	8.02442207	1.67789575	2.22636013	2.321222388
823649	1.68730778	1.20664802	1.4889622	0.51583181	0.78234138	0.178523027

823681	0.18543696	0.35188461	0.11149661	0	0	0.031510707
823703	0.52635513	1.22879905	0.73044374	0.0426421	0.26361503	0
823728	11.1367886	14.0411177	8.15895718	5.64969898	4.82028623	5.717225546
823740	23.5567541	21.84234	23.5839278	4.38887607	8.42642181	8.464259889
823847	2.49507311	3.54731787	1.87209618	0.41219823	0.62725535	0.487715295
823854	7.24046542	9.2760229	7.1520686	5.28104563	3.46786248	3.577911666
823908	5.86091727	7.37473539	5.2478907	1.89926369	4.15041979	3.354497896
823963	2.32745105	3.64792703	3.69445066	1.34754652	0.82891373	1.546829952
823984	0.48419827	0.2607571	0.21529024	0	0	0.082816026
823986	10.4095925	10.645211	9.72747616	30.3188595	21.7801181	21.64323761
823993	9.53207548	9.51356572	10.1359293	2.70149917	4.41901479	7.891958928
824008	2.617456	3.9242939	1.51294744	0.2394922	0.23030774	0.72908364
824021	11.0088826	9.58491103	8.84237437	3.80880233	8.17126957	3.561291678
824023	1.42411257	1.14733124	2.42201516	0.18459672	0.05071928	0.295771523
824033	32.7410005	50.5869897	24.8478639	7.91815451	5.75547644	14.86182544
824058	2.3892737	2.62487711	1.20258841	0.1736653	0.62030532	0.779118371
824134	89.2426421	88.1380511	73.3124802	40.6349537	56.1875935	47.9764223
824143	4.82776597	4.87726655	4.50611028	2.36450267	3.1935299	1.524546075
824156	17.2105986	10.2468416	12.1160747	6.65169774	6.62756753	5.49517451
824168	32.5802733	24.6874513	72.8742327	5.54248082	8.14386531	3.112995278
824186	6.53390793	8.3650059	4.21445811	1.48308615	3.03444574	1.43300916
824193	0	0.01517329	0.06524792	1.21737733	1.03296284	0.516322645
824226	19.3468378	18.0894528	13.5762822	5.24719552	8.8645365	7.793849493
824259	82.1977032	76.89954	94.2532626	55.4519011	59.9073561	52.61226581
824272	4.05765754	4.69573796	5.52565959	0.78613662	1.06455521	1.763430851
824285	0.55114636	0.65814592	0.33961777	0	0	0.029860866
824337	1.00110622	1.11340217	0.81159615	0.23229658	0.50840042	0.315727412
824355	99.8473934	81.8156248	88.9162303	36.9531962	66.8029239	45.22481943
824401	12.7752512	16.0730896	11.0946103	5.79311768	8.3339056	5.122650458
824412	36.6302737	31.3850806	42.3971131	20.3888987	21.1609684	14.47696636
824429	1.13248596	1.28075195	0.75530991	0	0.17794015	0.041506604
824453	32.6205396	40.8927317	31.7075849	7.20936333	23.1332016	13.28394672
824485	2.47471467	5.23168533	2.97168059	0.85626044	1.17983008	0.614305926
824565	0.43826856	0.73827807	0.6349462	0	0	0.036409302
824592	3.97363495	3.70743986	2.74683247	1.17406034	1.34235549	1.711237171
824606	2.93691255	2.26241897	2.11159534	0.50050786	0.93762372	0.131226967
824621	2.50975001	5.56552676	3.64480947	0.33134396	0.42209058	0.637078104
824634	13.950293	16.767829	11.4549371	4.61275173	5.59481408	9.914373588
824640	1.84144059	2.64777988	1.52665396	0.72543566	0.75226729	0.731146136
824657	23.0902207	27.409976	20.2217684	12.0983947	15.2961015	11.68708977
824666	8.16423819	11.0298197	7.34163619	2.65588292	3.92366443	4.144113588
824690	56.7158047	31.3351949	53.315231	25.1400197	33.0851349	22.76591602
824708	9.13180545	11.7613645	7.590276	2.74139005	5.27251689	4.903159093
824755	2.32600633	0.65417498	6.25908588	15.3404345	11.3523825	11.96503213
824757	1.63028508	1.26608391	4.14305016	7.6877222	7.56808724	7.986080714
824761	2.45497691	2.10707752	3.75117927	0.77897534	1.91260042	0.916172635
824772	24.8929158	24.8042609	22.6449802	7.60565971	10.3256254	14.21435081
824804	6.08932708	7.52679261	3.45959875	0.62643847	2.19299208	0.800611019
824815	16.7434793	17.6437363	14.868855	3.53142174	6.76896158	3.947347063
824866	5.72401895	4.51542737	6.15648835	12.1216193	11.0551191	7.753120316
824870	10.1718344	10.5301629	9.27195286	4.59219898	7.08277255	5.633522723
824937	2.85500663	3.54048313	4.5674179	10.0648499	8.67747078	7.748797771
824946	9.80973314	6.96157856	11.8592868	21.6599585	14.5010369	21.75300359

824947	2.30096344	2.34027905	2.64266284	0.64288425	0.64284317	0.597776148
824963	1.77837949	1.61008817	2.1619378	4.42263427	4.41910486	2.540926001
824981	6.46446128	11.9408453	5.99403015	0	0.01170659	0.587099987
825070	3.84847177	4.49287502	3.75467766	0.91267874	2.12291526	1.754815871
825183	28.7713846	28.9825596	22.4585506	6.44040861	12.6154243	17.29211995
825225	3.34944912	2.33049836	2.42627486	0.57888432	1.16307247	0.382602772
825255	0.19340367	0.67789636	0.57431399	0.02387564	0.01968002	0.126241283
825281	0.03182332	0.94395731	0.81183788	0	0	0
825331	58.5750711	64.7158775	66.6092362	42.0991068	35.7875883	37.70506431
825377	0.25787157	0.37773329	0.08121612	1.11419671	2.1812018	1.151473522
825446	16.0842218	15.2868361	27.7283559	5.04248488	9.04354627	6.645495803
825471	0.46748647	1.02716949	0.66255256	5.30220801	4.55776516	2.949153422
825495	1.67813367	4.15411367	0.82367493	0.05649961	0.05821379	0.013579042
825780	0.6926008	0.49623739	1.29931515	0.20817739	0.17159472	0.087557878
825789	53.9526456	56.2142117	44.096382	25.2516664	38.0738055	27.51322486
825811	7.7907808	8.84712198	6.71065396	3.47006177	5.05509569	3.036404361
825821	36.1119757	64.4473252	56.0649926	5.45106757	13.3558164	31.22610708
826317	2.06666046	1.48545488	4.38312409	0	0.10570702	0
826435	15.6149829	19.1161377	12.4877906	4.11027165	7.06066501	5.777719237
826473	9.184679	7.03607357	14.9219093	0.50942242	2.31723949	0.108830134
826695	0.61323116	0.76007309	0.80225679	0.02038165	0.15120012	0.078375963
826701	5.66740121	4.60201591	5.17100557	0.68923297	1.01152218	1.001976417
826749	1.28228522	1.66419897	1.17389423	0.40476677	0.60338746	0.604384722
826771	23.4055131	26.9241194	34.725265	9.07306055	14.1096254	5.855232407
826816	3.50813385	2.25027766	3.99895113	7.05568534	6.52917412	7.882964261
826838	61.6500096	35.7503911	74.134036	233.164358	122.322131	169.8250705
826839	13.6248275	10.4972882	16.7186159	27.5743132	24.325197	23.43397921
826851	19.7357527	28.7661377	18.4744722	6.86134376	5.59346157	8.946787262
826853	0.87218412	1.10687366	0.65357893	0.09746077	0.19280196	0.168649857
826875	0.23934187	0.61868962	0.25939668	0.00912472	0.06769121	0.00877209
826882	3.18891039	3.32421566	3.40676956	1.10384266	2.29402437	1.580487177
826885	6.0256963	16.0464068	3.39156151	0.06986266	0.20730891	0.013432558
826900	54.3071913	68.492387	56.8534908	20.9774779	26.9811418	24.76861461
826909	3.69266847	7.33344265	4.75423499	0.65748917	2.29786666	1.137744467
826964	39.8212404	52.3866417	40.3520897	8.38894864	17.3699086	13.46814019
827018	60.693886	60.2074445	47.565054	15.6236824	15.0175945	30.8634707
827060	0.45251961	0.52159163	0.27335826	0.05769504	0.07133458	0.120175022
827072	0.423412	0.80866998	0.40005822	0.13509817	0.05567879	0.032469312
827080	39.0736962	35.4397837	51.4796478	3.72413361	19.1917504	4.281934851
827082	0.60205997	0.73492043	0.61625704	0.02167789	0.12507927	0.083360543
827089	17.0875861	18.9847841	13.0424333	4.96915163	5.38186257	2.94403717
827106	2.43454201	3.03388514	3.03268374	11.7907334	7.23407577	7.380628806
827114	1.53076951	0.62285809	0.42854463	0.03674478	0	0.141299076
827130	2.61128655	2.00177259	1.00883438	0.09026158	0.26040021	0.34709355
827131	6.99566404	5.57869314	5.09986882	3.39349479	4.0787143	3.020696227
827165	15.0228086	15.3080672	39.2689937	0.8684781	6.576981	0.861006552
827216	18.0603651	23.2736719	15.0214999	8.47553853	11.4369973	9.922159093
827221	27.1664112	16.2500366	32.0113673	52.6437572	39.2179736	52.24853704
827222	1.24542805	1.54726003	0.9347895	0.10561183	0.41039195	0.681704266
827227	30.0533525	25.7974089	29.9096179	82.2759726	62.1498856	49.87537831
827239	10.4481094	5.33878365	6.33251219	1.8897318	2.70425755	3.078301309
827240	36.7360785	43.1881472	29.6964145	14.2829329	17.5664168	22.27360188
827260	33.3745316	29.5104136	40.1707423	16.3915477	29.4594704	18.49773208
827264	0.62010339	0.89535851	0.43524064	0	0.13250862	0
827284	10.0508453	6.12966888	9.47811078	25.1238893	33.4001271	26.55130603

827300	3.35656185	4.78957432	1.07536884	0	0.41221656	0.168270015
827311	413.878752	268.568769	635.910001	1098.15038	934.215647	1021.178608
827348	13.8104212	9.83866055	21.0204174	4.54153409	7.31904754	5.540740024
827379	3.2784086	2.76493261	3.09758415	0.32193546	1.41526546	1.341141296
827384	16.2668982	12.9790917	20.1718176	5.86896637	8.02532754	10.81724991
827385	6.80039113	4.5595772	6.3114938	12.7892992	14.7305191	8.556046201
827405	0.37985067	0.51153796	0.47467361	0.03970735	0.01963778	0.045807411
827407	144.699046	179.517995	139.018218	87.6897733	108.305229	80.39701719
827411	10.8781631	13.7002293	7.30290695	0.71894116	2.06455174	2.831515849
827417	1.83719036	2.62550048	1.98988598	0.63891437	0.89369042	0.837577205
827436	3.43558449	3.85107805	2.23003801	0.36205553	1.3280229	1.68810925
827438	16.4801312	14.0560918	12.4018851	8.48749138	6.63117651	10.41210883
827488	1.44781707	2.22965873	1.41681679	0.05026858	0.31766805	0.177195074
827516	15.9092614	13.2637551	11.1322455	5.99346171	9.00221615	5.505760287
827528	31.8963702	27.7933173	27.180558	14.3683236	15.6992097	18.74234676
827561	3.13539332	3.43100938	4.58364416	1.55206431	2.7828564	0.969085621
827598	0.31341247	0.8644572	0.30872694	0.00864367	0.03562365	0.016619261
827601	15.1941517	13.0166689	14.6110431	5.09085601	5.35582223	7.307553273
827608	23.0322713	26.6077577	17.1066478	11.003244	10.4227119	11.93417596
827610	3.49906536	5.18707621	2.87161878	1.36648535	1.31807516	0.936344259
827632	14.8015364	19.7745402	8.79547386	0.69347311	2.59129969	1.762981264
827663	2.11137053	0.78031625	1.63638224	0.26485226	0.77967932	0.157620014
827678	1.31845792	1.9439985	1.50799722	4.47492153	4.0036533	4.950527214
827707	2.39055579	2.87140561	4.42705571	8.841604	8.03710518	6.255779985
827763	20.1951138	21.8088866	19.3518844	7.48808858	16.904739	7.650858917
827835	16.9408526	22.295854	15.9089588	6.35365397	11.6319566	9.065728074
827842	0.78010759	1.03367346	0.31508757	0	0.0318129	0.09646946
827876	0.19124446	0.35267264	0.3613923	0	0.08757136	0.034045061
827890	5.27267773	5.6170765	5.33209669	2.75039475	2.82018345	2.134397786
827914	17.9388916	19.6308404	14.9499455	7.62982526	10.0025821	12.62662321
827936	8.41428972	9.28297587	8.72929159	3.58579543	3.2967083	4.684684998
827942	9.18680852	11.9907949	9.48061488	5.66337016	4.84565187	4.782057585
827948	18.5464943	17.3334851	14.5274391	2.60654763	2.79305431	10.25457268
827959	40.8261731	31.9444779	25.7720222	16.6450619	22.3095416	17.09311834
827972	5.81783412	2.26294489	6.7448997	14.1244814	13.8768114	14.75427503
828019	80.6529705	93.4036167	69.2736835	15.4143958	16.4267274	20.42911935
828024	7.71941486	10.7935126	5.20356639	1.44356516	2.16788148	1.25470344
828035	13.1228091	13.0769791	7.6283843	2.09727811	6.83672394	2.52281798
828038	0.4420441	0.48563354	0.16486683	0	0.01242888	0
828045	6.62155151	6.84936193	5.72007508	2.70866301	2.82070353	4.736467859
828064	2.49560488	0.64379847	2.24340027	0.10477279	0.57214324	0.037771429
828085	8.74494301	6.44835873	8.68920739	18.5124246	21.3528176	15.66491798
828090	19.9946454	23.558665	21.3496582	7.12949426	8.68676104	11.75821079
828092	0.14978086	0.62294145	0.51553541	0.06933906	0.02286169	0.053327542
828135	0.48043358	0.53869121	0.15911113	0.09630139	0.0264595	0.037031914
828140	14.6537002	22.8678234	12.238818	2.45047992	4.08247063	6.955160622
828153	2.56496718	3.01370093	1.69864192	0.23102625	0.12419242	0.347631888
828160	2.93216897	3.20011381	1.13004862	0.0250049	0.28855159	0.024038574
828252	16.0526575	23.8777414	16.1426646	3.08491016	9.47236584	7.865959678
828262	5.92558466	5.96403829	6.08560013	0.85873171	3.91271703	2.178523399
828263	14.4847887	16.4572092	13.1848924	7.89236201	7.32096603	10.8473818

828278	2.421852	2.72351833	1.39638641	0.64268312	0.82518045	0.974296232
828281	3.24401819	3.06964246	8.63594866	15.9604718	13.6364558	13.48472836
828302	3.79740224	3.59243457	2.98996007	0.56795532	1.24839859	0.829120951
828310	61.3965558	44.3822815	75.4205659	24.7436374	46.7431075	28.20507223
828324	10.6118077	12.1194805	8.72732043	4.34018824	6.50453477	5.938234691
828369	2.14930247	2.39932716	2.02256666	1.03350683	1.00004523	0.777573842
828401	2.63976937	2.31185132	1.44708909	0.72631047	1.29048181	0.449978134
828412	5.07292497	6.54533471	6.28321158	4.80396583	2.78026603	3.100712526
828417	78.6186244	71.3718479	87.6763173	19.1268668	40.8669767	31.24250637
828419	0.36900631	1.23460772	0.51505607	0.02174156	0.00896048	0.010450677
828421	10.8366058	9.98392374	14.7948287	2.35858028	6.60997691	2.015495173
828439	257.834187	325.037651	258.187451	87.8197594	56.533514	193.8683445
828447	2.95692326	2.093479	1.95124684	0.74223085	1.765193	0.61996712
828457	90.4943639	97.302341	122.871988	40.1296628	57.4640063	33.24117503
828467	1383.88869	1014.56707	1183.68383	376.351495	704.490596	487.3267756
828479	7.5743438	9.06090657	5.66164365	2.44360041	2.94935043	3.216120894
828480	1.71794771	2.55267816	1.55312645	0.06408181	0.59423426	0.184816046
828482	56.3472193	59.4424345	46.2989381	5.0893153	4.47212404	17.44011986
828501	0.90050001	1.18050594	0.48618965	0.03923525	0.21021327	0.122586713
828530	1.08807475	0.95141574	0.56648244	0.02878341	0.02372535	0.166026415
828553	9.72843069	13.3866637	7.9616257	1.41705687	2.30983153	3.321548742
828556	18.0124964	22.5450362	18.9481426	6.07816008	10.2789321	6.102009793
828667	30.0882234	31.3849856	46.9841314	16.800257	22.7304187	22.889583
828702	24.1679631	25.9142043	21.1644321	10.9744101	11.4468713	13.61926105
828713	12.7117128	16.9306132	14.4227508	6.92870227	7.16022625	9.709728453
828746	25.6553348	28.4641061	33.5731373	15.7812516	20.9394539	18.44068346
828776	18.948681	19.0631895	15.7201853	8.07831651	12.4944658	8.411849891
828783	5.43902522	5.17535395	4.77377611	2.3889069	3.12175696	2.96875843
828803	0.82696744	2.45298699	0.67717441	0	0.02945216	0.034350293
828851	19.1845077	22.9705311	15.8661629	11.0644566	12.0909887	11.84104011
828861	127.780879	87.5375425	134.583052	46.1548139	90.084368	46.20588875
828886	17.9495324	17.6865746	13.1590299	4.89823025	9.00106589	5.267212284
828914	1.0337093	0.94636844	0.92243308	5.73186969	3.43608559	4.150660371
828920	7.38721187	8.4598467	5.73337558	3.44118549	1.92034416	3.616416957
828930	2.35333371	4.11251651	4.91942241	1.2486277	1.36793523	1.443487744
828934	1.57498684	0.68578741	1.06164416	0.19917404	0.10260845	0.119673053
828957	65.8585811	55.9107976	67.5480411	36.6696025	39.1178989	45.37605403
829023	3.62449204	3.89894261	0.59454496	0.03262604	0.02689272	0.031365192
829029	14.3725212	20.0585263	12.4744564	3.97150951	8.33280686	5.536142126
829032	9.29194039	7.49412054	7.73426203	35.8704014	15.9161223	26.4662034
829125	17.211584	15.4044359	11.528882	2.83771973	5.04402915	4.050241169
829129	7.45034475	8.21819677	6.45913642	2.54483089	4.62554739	3.312164246
829164	6.54915372	6.37903482	4.67284216	1.18148139	1.69524058	3.13402903
829167	109.965213	105.121108	149.945958	52.61669	75.8334893	33.98503764
829179	2.07152387	1.74390371	1.44732738	0.52468576	0.74624621	0.840681807
829188	29.6313531	29.5104136	20.6649905	13.3394106	12.4494325	18.98294851
829210	0.61876569	1.74119455	1.05644923	0.0140712	0.05799244	0.01352741
829226	17.3043213	17.917301	15.2275332	8.53098803	10.3591179	10.44166127
829227	5.86441521	5.20033485	3.70657844	0.86912364	1.24032391	1.446599709
829251	4.12247594	3.67570067	7.96895238	14.662762	11.8094806	8.79766279
829313	2.2776254	2.18330899	2.08870619	0.23155373	0.35786845	0.222605249
829319	1.84114207	2.38574267	1.03334722	0.0203977	0.05043973	0.196094191
829376	104.461047	106.444544	87.4350406	60.8809117	57.7552993	63.28945814
829413	17.933466	16.2145103	13.2410688	5.17307645	10.3819624	3.828443632

829435	8.1704542	5.88747361	5.77232514	5.38620338	3.2064462	2.198617127
829441	3.59920267	2.93090156	5.0711961	11.8271188	9.39456508	6.786624778
829448	31.5766853	36.3700989	31.1616932	19.5147508	21.7362015	21.07407889
829487	3.87886009	3.60010588	4.65486589	9.18886039	7.88375083	7.083670238
829495	3.60514562	4.39114955	1.90061645	0.81270819	0.94901411	0.944071771
829521	9.6283798	3.42396709	3.54178014	0.01388268	0.09154477	0.013346175
829563	178.100795	151.397728	203.290578	37.3417912	148.422283	58.98766106
829575	17.0442287	22.2582064	12.8762131	3.46320173	9.46637123	4.428757215
829599	46.4858491	45.3878881	46.6152807	25.1000762	32.6438133	29.5092288
829610	0.22261824	0.34462186	0.10516973	0	0	0
829615	53.9013866	46.8580107	36.5056805	27.8717929	32.8479694	25.3241211
829617	3.75353663	2.72257641	1.73902029	0.31935188	1.03744654	0.487604772
829686	3.38623967	4.79283475	1.90397769	0.18849846	0.35514042	0.465978503
829688	0.7368452	1.66857075	1.12245077	0.25339801	0.44987125	0.168649857
829691	0.1479278	0.23158357	0.10482651	0.88683171	0.45044804	0.663614093
829728	225.496511	155.763309	322.560015	703.532133	460.154746	604.8028767
829747	11.197088	14.4657196	10.1555823	4.75368843	7.41981753	6.867124772
829779	46.8524933	26.3755544	31.5849979	15.397393	17.4188396	19.56561289
829781	5.02047611	5.44459981	3.08102994	0.73237283	2.00074866	1.126512023
829784	2.0234513	2.0707224	1.80708683	0.58684632	0.86872305	0.644762776
829788	9.33000189	16.0260932	7.30500464	1.41816095	3.24708297	1.24974263
829838	16.8390133	23.0322876	16.6777302	6.85230974	5.38642043	11.68076164
829841	0.25866204	0	0.29584687	3.10578537	1.64364332	2.442895477
829857	0.73012489	0.38240682	0.95456542	2.23395493	1.37877157	1.893716754
829862	11.7907042	12.5894639	11.3713785	7.44234153	7.41534312	5.837787515
829865	4.68227382	5.67654118	3.50798568	1.79936011	1.58910164	2.992868669
829878	6.78149656	10.2144246	5.20747243	0.24848991	0.51205798	0.836104247
829880	101.405041	104.700425	175.335683	65.2372803	50.2687705	63.70471559
829900	2.2276836	4.66581648	1.5748242	0.24928699	0.32534365	0.179739904
829910	67.5360653	68.622621	71.3062312	39.1885948	55.2917934	34.36492232
829931	1.2618228	1.46779372	1.11332822	0.03607949	0.5551336	0.127179009
829932	36.1387155	37.2827793	28.5992802	16.6920213	25.7770125	17.18101219
829933	1.45549357	0.88560999	2.56001399	0.10158852	0.21532253	0.209276994
829936	60.4361457	56.3553587	35.3611706	0.56315377	1.44230988	5.839283069
829945	1.23406804	1.21453322	1.46559921	0.22217044	0.43950911	0.266980727
829991	0.65589516	0.77266174	1.02290884	13.8488174	5.30232085	7.936968587
829995	3.50782826	2.34283894	4.53153051	10.7749761	7.43070612	7.267271208
830022	4.66964514	7.33279342	3.28767584	0.15090334	0.84305623	0.225666972
830032	155.976316	318.443442	123.601491	13.0127811	26.1309933	37.70987562
830033	0.33891794	0.24110426	0.31497494	1.69603692	1.478135	0.744280217
830051	6.22910537	5.20379736	3.99725701	1.19432481	2.87015145	1.245198111
830060	14.779376	15.0003656	13.0902258	7.40049795	9.37239897	9.836072508
830082	296.863967	240.088283	344.141206	1101.77194	633.457494	952.1230062
830088	69.4639093	97.904034	48.0067439	12.541034	30.7855297	18.7627431
830090	24.0225246	26.9341761	22.6041653	14.1633107	17.9440089	14.82392666
830105	4.73330046	5.83233617	1.65950664	0.24438748	0.77396012	0.222577617
830136	6.48922734	5.37526073	8.68718133	18.2600111	14.0720122	13.22562491
830149	6.90915933	9.52137052	7.5301936	2.84779048	2.76825762	3.587378941
830301	7.5948475	6.50037605	9.37044888	23.0776085	15.4083743	16.8154018
830332	2.51006075	1.43645532	3.29140543	0.21030862	0.37729429	0.083251068
830371	0.89826135	0.65789188	0.94461689	2.49972775	1.54534104	1.618947265

830399	78.1532873	83.8623463	66.4870191	50.238911	44.112409	58.24597283
830414	3.00307213	3.94152386	6.9178152	23.510921	9.41284445	21.64194058
830426	3.97451204	4.4900122	6.34148567	16.9622338	15.3035203	8.176723058
830527	14.7743703	16.8623698	9.34199104	2.97855905	4.21869432	4.748893202
830559	27.1665019	30.1361105	27.8307644	17.143336	15.0784213	20.89294469
830584	0.49231282	0.94846322	0.3707787	0.04624254	0.01524657	0.053346581
830589	31.7485787	33.8194443	30.9633531	10.9368682	25.5526153	10.96276723
830591	1.102874	0.93202083	1.03059267	0.09425739	0.14243844	0.498381294
830594	1.01243507	2.28247087	1.02634461	0	0.14648371	0.026283865
830597	1.67175293	1.66963861	2.88626032	7.68287348	6.44643998	5.83301929
830602	3.2053519	4.18317261	3.90257006	1.56155448	1.24117562	2.090967765
830609	1.82400788	1.04201298	1.40744546	0.50438841	0.83150613	0.70461469
830613	40.009641	38.3211798	38.8242249	21.2742852	24.155727	29.55292088
830615	24.9332009	30.5628841	25.2072996	10.9036485	9.80996021	14.64435193
830659	0.40467262	0.67321645	0.45882292	0.05245459	0.13588718	0.122466693
830662	12.9192761	13.4829346	11.6285437	3.65155281	5.36595936	8.603448534
830680	1.93682908	2.9242631	2.58653774	0.47686654	0.86706081	0.795524511
830694	0.66511248	0.86987907	0.2842886	0.123162	0.01691982	0.059201178
830696	0.49334302	0.83855208	0.2902332	0	0.01989084	0.03479827
830711	1.63847198	2.34478859	3.19804899	0.13972531	0.71406402	0.322381388
830722	23.7364294	16.9758407	18.1548654	4.18243922	2.68401338	10.86591872
830730	65.0862653	69.1612945	63.6418538	30.3120256	44.2739212	41.50660371
830762	15.4704592	13.9498276	18.8827478	34.042455	31.7747549	21.12982921
830788	1.44396185	1.50196891	1.36431845	0.43805658	0.45955334	0.287132539
830789	1.65301054	2.27410502	2.04023729	6.15776335	3.67548499	4.416649039
830836	5.51442764	6.34021831	5.09349097	2.19367415	2.05198342	1.860096132
830867	1.95557733	2.25321516	2.62285423	7.43147929	5.24902198	4.731158457
830879	35.9093861	42.7597664	29.7534931	9.90791652	13.0016824	18.23975454
830882	35.137771	47.7451749	47.3101346	74.9020911	82.0999154	88.29232859
830885	0.04009036	0	0.79546179	2.20855691	1.15652171	2.048269712
830903	5.8478971	7.89034685	3.91602441	0.77273656	1.30422039	1.06124839
830904	5.61920516	10.447137	5.14110437	1.25240813	1.11382382	1.520852655
830917	0.36602649	0.33174894	0.32854598	2.20620131	1.49587046	2.246373882
830920	41.618344	45.2067086	37.7330511	22.7448325	25.9878221	21.28104905
830985	0.89310464	0.73990066	0.67784223	0.01897808	0.03128618	0.200691271
830996	10.9424523	10.9778739	6.80055087	2.84150714	6.42145977	2.230885099
831013	82.614673	85.4778098	54.6428934	9.31882935	20.4876942	23.32931023
831046	170.568699	142.71236	155.700491	64.4620443	109.902993	70.73785016
831051	17.5982705	25.1318526	13.8087042	3.45328354	5.95328766	4.827891752
831084	11.5026473	12.6369095	16.9094703	2.29064154	9.97837992	1.614887154
831130	6.04835445	7.09052477	4.5200912	1.79794419	3.75035323	1.575604787
831132	0.49345794	0.6599688	0.35474835	0.02781006	0.06876914	0.053470665
831134	3.63239869	2.62023327	6.57917415	0.19544294	0.51258478	0.461184486
831145	0.3426008	0.88719144	0.58575055	0.0105735	0.03486174	0.020329765
831149	24.8378746	23.4527306	27.4545215	7.49883704	19.1562359	6.538779556
831153	26.6875619	42.1619569	20.2055323	0.93004204	4.00131608	1.308439346
831157	36.7783127	27.5283464	35.3128657	93.5775713	92.5996277	63.67096506
831177	4.09536969	3.37931867	2.72046747	1.79654871	1.88210517	1.682549573
831181	8.50217382	7.93010835	6.37059462	1.87363738	4.26864502	2.651500022
831188	5.38185157	5.55202817	3.35900003	0.45372871	1.12198713	0.708815564
831196	3.02317301	3.33108177	3.28615424	1.14440083	2.14385719	1.300206863
831216	62.1311266	65.9740839	62.4256293	41.5993879	33.1981762	51.53484154
831234	6.98116656	13.3897334	5.73947423	1.07448133	0.31828568	3.115012774

831258	5.02033997	5.50216329	4.17467979	3.08988225	2.25105005	2.610415318
831268	3.72328184	1.50359686	17.2002644	127.083652	51.2436174	86.41198087
831309	0.80309958	0.5776158	1.00991857	2.38155922	1.70378076	1.490349864
831313	2.86856217	4.10567672	2.15172291	1.19212536	1.27118673	0.936854678
831357	6.85170268	7.45507836	5.27225379	3.55253338	2.43630612	3.565515163
831375	8.54551847	14.6371652	7.40348174	2.44187167	3.3254388	2.279461024
831387	62.3962124	80.6933688	60.473547	12.5537992	22.2009931	14.0471207
831411	2.77816544	3.56384918	1.20505882	0.20126028	0.23106545	0.739379567
831424	2.56591927	2.36107036	3.9520927	7.69341627	5.26845901	5.087166154
831434	7.83032686	9.03191824	4.01662362	0.03166879	0.30019242	0.228337062
831440	12.8231697	8.09290361	9.24803521	1.3483016	4.09930372	1.806174735
831442	42.5829787	41.0150953	46.0280879	15.7749387	37.8893047	13.83064569
831444	9.89190135	9.60032502	10.7504413	2.45807242	3.91165094	3.279375351
831450	8.70506272	7.51004321	5.70557848	3.20213137	3.84043482	4.935186759
831454	742.217976	406.239882	580.052837	172.700489	259.365558	266.6384223
831457	3.42555692	3.70264782	2.95322597	0.78779252	2.01553057	0.66882679
831460	196.327734	256.785968	190.240031	71.0551884	126.299254	128.8025218
831485	1.64321493	2.68348028	3.48281794	0.40296781	0.35588029	0.553421383
831496	0.23429128	0.5019192	0.21030024	0.01518469	0.01251631	0
831499	9.41533774	6.16266927	11.1796774	19.8028855	17.6719618	20.09399118
831500	33.3359953	37.2444637	26.3759017	19.7729546	22.7822332	17.49636546
831530	1.10410232	1.11698558	0.60040534	0.22651495	0.24611753	0.138575307
831590	5.63393313	5.50433368	6.32956064	3.32442387	2.74022834	3.6325555
831595	21.4287546	34.2704525	22.2149975	4.47791516	14.8411998	3.636646703
831677	2.0605203	2.49996627	1.54048443	0.37599956	1.06870959	0.679312283
831696	36.8679652	41.1723355	30.3511866	18.4642544	23.2939828	20.70111754
831716	3.85867927	5.3056391	5.66367786	2.04472346	2.25585322	2.600778083
831734	8.4852252	15.6478695	4.84292423	0.13841593	0.43881664	0.051179536
831749	1.68832415	2.22145581	2.93999561	5.19119349	4.88723624	5.076200552
831791	14.206802	13.241353	10.3527718	4.72215255	8.91535167	3.944488863
831807	18.2319721	13.3065138	8.55295118	1.19816129	1.15788803	2.561890851
831810	6.62014397	6.55420997	5.76931968	1.71623635	2.92914723	4.949735283
831852	0.55213942	0.92306447	0.60107245	0.04667581	0.03847355	0.044872004
831863	29.2712178	56.0171623	21.5523163	3.34495293	10.2437174	0.798053455
831885	2.1934807	4.81955438	2.46725799	0	0.74400061	0.564027019
831890	1.0974959	1.84747213	1.03696224	0.06883545	0.51065197	0.022058425
831901	9.73744214	10.7341919	13.0146353	29.4415041	20.9262352	24.7242999
831905	0.75653172	1.2189942	0.8577652	0.09806337	0.16166171	0.141410511
831919	2.82757862	3.26939301	3.19110193	0.95022977	0.65270621	2.631772428
831922	0.94858904	0.90001986	0.61109216	0	0.06910297	0
831964	4.8918621	6.89257554	4.53749612	1.63678822	2.26276127	2.316935789
831985	52.6303032	65.8120503	46.8603536	12.6464687	30.1344188	13.58511766
832028	22.4538426	27.4317938	17.0822792	6.6557422	12.5368821	5.766863964
832034	803.761528	815.372045	954.690588	1589.41128	1282.98281	1790.705462
832044	87.3197214	79.6684496	148.945813	10.1424318	44.377659	9.500002833
832094	10.147446	7.05382766	15.9226616	32.911269	29.1968974	22.69456678
832129	43.3734361	41.8592937	38.1765158	16.5182158	31.0176332	13.9666267
832149	14.3984095	17.5230883	15.5549982	2.43133369	6.08432749	4.590669607
832176	5.18830442	5.34021338	4.79505191	2.44849465	3.12152091	2.353871666
832234	50.1283906	53.1288624	42.1337627	18.36037	23.9210597	19.92316978
832267	67.9309273	68.4458544	90.8826438	45.9615669	57.2289598	39.41470875
832290	2.18382963	5.31560209	5.19630505	12.9720142	9.3224582	8.501051768

832307	2.30925643	1.34678624	1.1852224	0.14781782	0.25891434	0.159868516
832321	27.2465622	45.0278895	21.3431059	0.41162723	2.32980955	3.614240281
832352	32.5140424	56.0428886	34.7781424	12.8760754	17.8486362	11.87863133
832358	0.40007309	0.57240311	0.23735298	0	0.00994079	0.06956414
832451	18.366211	26.8689221	21.2595972	7.1254048	9.79292974	14.5563369
832488	131.953773	121.018614	168.558944	72.5063838	93.5396102	82.00773719
832518	14.1694813	11.1808042	15.5498359	62.0053456	31.3734899	50.49103851
832524	31.1539559	21.8666175	43.7573825	12.3040335	15.6462121	5.476175727
832529	1.30571018	2.16013506	1.00146779	0	0.18053881	0.153137354
832538	210.073677	140.991649	131.949797	91.0297354	110.889359	55.79940018
832584	21.3597994	20.5702514	28.533453	13.3428064	9.7599007	14.01644264
832585	14.2869601	11.1326533	15.1357796	6.65550711	10.4924289	5.579007726
832590	39.33473	29.9934974	21.4659369	1.24783591	10.717551	2.447210161
832597	1.95262961	0.54275539	3.22307056	0.12197798	0.20108598	0.161238131
832618	20.9012663	23.4341434	34.1404409	10.651513	17.8500989	12.22006743
832620	123.359855	110.877213	81.5772496	22.114087	25.8530674	50.88257949
832624	0.62866868	1.12686785	1.09419896	0.04288919	0	0.22677449
832634	2.6112607	3.34959626	2.58638974	0.30770689	1.47424798	0.684073202
832636	351.468343	255.319793	326.484569	88.3352503	86.3633068	61.6326199
832667	534.08823	1144.8172	239.01531	4.04852256	17.0010818	31.34690916
832669	1615.16291	2734.86357	973.267299	111.740832	276.497784	449.0733801
832703	38.6145179	23.2407547	55.8797596	91.7923182	74.9748738	70.77358568
832710	43.5376141	44.6154493	40.0072084	23.4357856	26.7927902	31.05442084
832720	2.96195313	1.43034187	3.47516369	0.29533471	0.66075494	0.324481593
832722	1.15387104	1.55921303	0.64323448	0.0747837	0.34519567	0
832743	4.85708205	6.54054112	4.44426243	1.75253197	2.77987221	1.656488676
832781	0.29782124	0.34763835	0.29898173	1.31897921	0.80213938	0.680393876
832873	5.56675127	3.92347976	5.9461802	16.7660076	9.09654082	11.23419904
832875	31.1016178	34.207405	27.2453465	16.3369462	18.8184708	22.01430851
832912	1.59216967	2.53265472	1.51218686	0.39771275	0.79740813	0.733685194
833302	16.6135853	15.1838276	21.9435185	48.427218	30.4885251	39.00102215
833437	3.58158977	2.08456257	2.54598847	0.71311719	0.77973772	1.035333261
833498	6.14545456	4.30762645	8.02134339	15.9226323	12.1317699	13.38620865
833540	1.37972078	1.96361552	1.58014626	0.35226564	0.4020405	0.299576951
833613	42.8250994	39.2446344	43.9328448	22.9056957	23.5545194	27.02711108
833670	0.42838403	0.28646828	0.35196175	0.06035665	0	0
833672	10.4210825	10.4935061	7.65564044	5.29131706	7.34600173	5.163672514
833692	0.26560875	0.40675167	0.24335398	0	0.04299802	0.030089362
833738	57.8911923	55.8295399	87.7667266	182.901564	125.548651	110.7034442
833744	1.59998889	1.96934431	1.13380473	0.23043801	0.33240116	0.203071597
833757	5.26836921	4.60216589	3.5777838	0.97218124	1.78835955	1.29933716
833949	54.046068	66.5476021	51.9184139	25.0856101	38.0187675	29.96835739
833951	0.53965818	1.08855412	0.5098927	0	0.028357	0.022048661
833962	25.8560244	25.4955963	26.0454915	7.17450104	16.8435925	6.527744798
834022	9.9247596	5.79530953	12.9923549	32.2563433	21.9320281	22.77341982
834109	2.34106764	1.29322406	1.08722638	0.32574215	0.76310533	0.148335976
834116	6.46901947	8.04107892	3.93303063	1.11659799	1.41322875	1.689812787
834120	1.85530399	1.43469285	1.63383848	0.1050679	0.84679944	0.47136837
834143	18.4658486	14.0112887	19.9449473	39.5025293	33.7213824	25.63281251
834147	2.8932007	2.04556656	1.56078136	0.17327808	0.39787859	0.559238521
834161	0.23973338	0.31125924	0	2.54248267	1.79298468	0.733268244

834177	40.5668233	34.8943819	40.758852	7.64354364	25.1318722	9.950768838
834178	33.541949	33.5012768	52.8253559	5.028899	19.3878344	8.987034573
834193	1.15252646	2.14075067	1.12263661	0.13861216	0.16503367	0.310929374
834195	1.80894458	1.98732243	1.23838075	0.23869173	0.30090691	0.148478908
834260	573.951817	939.948325	543.494742	134.377153	115.012417	247.7486142
834277	18.5877478	21.5921751	13.514975	6.48222628	8.38790534	8.958094969
834281	5.63595955	6.51886992	3.32428066	1.24378664	2.08578847	0.508524615
834294	8.23125112	3.05379705	7.07537014	0.72993912	1.49776958	1.104852041
834364	35.1403732	23.8851923	38.7382433	72.2594547	47.720312	70.63669286
834383	11.1783942	10.8613068	8.35039692	3.64871583	7.54884119	2.714391886
834410	8.64546482	9.06129479	7.53223422	4.00741467	5.57414713	4.815683304
834422	0.71031635	1.19492592	0.65715598	0.10550011	0.33203197	0.165964946
834459	107.810261	140.018291	93.9198666	37.293503	37.1802817	64.04073982
834466	7.04537385	9.09190349	10.8383597	3.1641748	5.05724627	1.545677894
834476	1.50589749	2.43952753	2.06190925	5.29350159	4.92232896	3.03745644
834492	1.48357461	1.73272388	1.14317176	0.10268678	0.15389418	0.206411218
834497	28.5964891	46.2702721	24.2229907	8.60493727	9.20080758	14.57792911
834543	21.7845784	13.8227987	18.4542228	10.3544437	12.2864675	7.730073195
834564	13.8176322	13.8683055	8.09614861	0.79943431	3.30877541	1.635191217
834570	13.5169402	14.3768348	36.6586412	0.94743004	4.83540759	0.187154025
834585	30.7483619	42.52305	24.2759441	9.64914478	9.80834776	13.46440765
834592	1.7890381	2.33397542	1.16212361	0.27054907	0.74866276	0.492320029
834606	1.35202986	1.39797825	0.322048	0	0	0
834624	1.0905153	1.05360399	1.8341988	6.34594097	3.72682691	6.303092607
834635	16.3058509	18.7001319	9.59016348	4.21235792	7.05132443	5.102101144
834639	0.90459613	0.68643728	1.33932696	3.85614485	2.57070786	2.115035523
834712	6.42656394	7.79213793	5.81292434	1.1513366	2.10736958	2.164854233
834716	4.37977996	10.7959202	12.727598	0.02385366	3.10657935	0.068795476
834727	0.58166253	0.3134813	0.03110832	0	0	0
834785	1.72300355	2.54359359	2.12823067	0.20938093	0.45064321	0.402578644
834792	1.60677896	1.27559802	0.99732823	0.22803762	0.30074391	0.233840021
834800	3.55992229	4.06196434	2.02593443	0	0.96924757	0.205534764
834805	13.384904	8.18903756	9.09250098	3.70927148	4.53685731	4.342376786
834811	14.3819514	11.0223187	10.7027721	3.67075939	8.47196811	4.890049263
834884	0.07165075	0.17559754	0.14581272	0.00714426	0	0
834900	2.41281705	2.76691001	2.1071657	0.53579517	1.63304377	0.646856162
834901	10.1613012	10.9255756	6.91849488	2.50187586	3.06097125	6.643932755
834912	2.37571786	1.90269865	1.6192556	0.7639881	0.71692763	0.711864619
834913	36.0200859	34.6366568	67.3920232	6.71352648	14.6408283	3.750800281
834938	2.57034321	1.8396102	1.62959617	0.74882701	0.92138258	1.001583734
834948	5.43612677	3.94590245	2.30445192	0.89653259	1.08903259	1.678409112
834951	93.2968582	115.170095	88.9901944	56.0700922	66.2055972	49.5450493
834976	1.55173995	2.21056163	0.49945835	0.0221033	0.12753389	0.106245573
834981	6.11424795	6.1198142	3.50185329	0.83425556	1.56500384	1.852932226
835006	10.0828999	10.757712	8.50206303	4.21734187	5.45785742	5.582091416
835031	0.44389886	0.49030725	0.37634003	0.18661357	0.13074723	0.167441696

835035	140.855236	147.812318	128.633144	81.2440215	89.2530962	100.7826093
835043	0.62286431	1.36856843	0.48762216	0.02306774	0.09507043	0.110881399
835081	31.907456	35.1309097	38.458035	88.1459794	94.6838751	52.19823843
835100	3.30687818	2.84319036	1.41280991	0	0.12289391	0.609161666
835164	42.9291008	22.691046	28.7789941	0.59359451	12.5457213	4.667663896
835220	6.91163105	7.19919298	4.65137834	0.8029305	2.08999846	1.625054632
835230	2.5420979	5.34842015	2.05444297	0.2486887	0.61496115	0.328732301
835246	207.059067	62.2030269	134.720655	13.1791625	30.5779245	31.27240479
835264	1.741303	3.24685318	2.47543617	0.31061239	0.73394976	0.736568028
835281	21.784737	24.7713835	16.7995027	10.6502706	11.5170313	9.80424727
835288	5.60278296	7.77507365	8.99178469	15.4978709	15.2520126	15.75079569
835304	6.37515817	6.13816604	4.62070499	1.18172378	2.07335928	1.769000901
835330	33.920755	41.9575882	32.4478132	16.7442629	13.5142833	16.98660187
835372	0.40476043	0.71564621	0.37048429	0.11476931	0.18920218	0.086691008
835418	76.6783513	81.587958	54.6063496	20.9047005	31.3009216	29.72367801
835423	26.0993759	27.4116851	35.3436603	92.2165238	71.5183883	67.05936656
835468	6.5074116	3.25099142	5.02214837	1.79972089	1.93299191	2.341846273
835504	89.3786811	69.3138703	86.6310703	38.0356388	58.8949792	36.39937833
835508	3.91551562	4.6513451	2.98971828	0.2558322	0.74826694	1.428070469
835526	10.555397	13.5245278	10.3963766	4.61301731	6.34376561	6.410771621
835550	23.098355	34.1899271	27.9903644	8.21077521	8.16243117	4.804718896
835575	1.55608758	1.84361241	2.04683221	0.65257055	0.40749652	0.475266455
835588	1.65959738	2.89800517	1.70010754	0.22644295	0.70927191	0.914306306
835633	2.54547975	2.59673741	2.02570798	0.49099836	0.75277181	0.349297423
835638	18.5249517	14.7374197	12.4591647	3.31206418	4.65569424	1.705750838
835639	10.70167	10.7653989	11.1469124	5.59883844	5.57928459	5.583307661
835641	3.44253535	5.66524942	3.37263339	0.7680996	1.85414512	0.826322785
835646	0.15088748	0.24864946	0.05702604	0	0.00806071	0
835667	3.31864018	6.23285879	1.75224522	0.22141088	0.1564309	0.25846603
835676	0.20780435	0.25584862	0.11848264	0.0185766	0.04210846	0.017858704
835699	165.6472	168.184557	158.346257	95.4214816	127.76552	66.01980343
835701	225.309471	237.942835	260.629275	99.1742034	161.225487	51.81819727
835719	0.06149245	0.14637165	0.23240305	1.1026262	0.75556124	0.561935558
835754	4.47102022	5.25300645	5.01649386	1.50252109	3.51641429	2.618075781
835771	0.57964195	0.56758238	0.5833888	2.48270565	1.9791073	1.742963309
835859	12.102431	9.75398929	6.4749963	0	0.03606895	0.77824882
835860	107.215375	79.6758896	164.64806	4.54560722	20.8559281	4.824163752
835877	6.5965994	9.86706319	7.5109325	2.82009173	3.23346924	3.267233007
835886	6.1232379	7.51117686	7.24075293	0.90083432	1.32451688	4.096046419
835908	0.47023639	0.74771896	0.35076312	0	0.02644312	0.04626123
835924	21.3772376	22.6799066	29.0247753	7.66766602	16.3014829	7.505885687
835942	4.17651036	5.18386041	3.60191144	2.38432645	2.6679154	2.170376432
835952	3.0779262	4.16781745	4.74773961	0.09278321	0.8412643	0.553024949
835981	18.0641177	18.5467222	13.6821389	8.94227811	9.71928157	10.78327755
836019	1.44317972	1.19094405	0.89229624	0.12930985	0.39081696	0.306637804
836047	23.4347689	25.6174137	19.5321869	11.3471282	11.6255769	16.79118543
836099	35.9703901	33.1259563	38.9234	69.5806082	75.6125717	57.93024098
836109	13.0415239	28.4580269	11.4749006	2.62371897	2.73754072	1.277126268
836119	7.0593945	9.12092009	6.27076328	3.47978598	3.37289531	2.38508096
836132	15.4742341	13.6114309	15.1646487	41.0404968	23.3357104	39.94698326
836170	0.65596432	1.40926754	0.82637846	0.01889502	0.14017167	0.29063705
836175	32.5991174	31.5122955	38.4249443	77.472498	52.9091536	71.27539445

836201	14.35335	20.1671665	20.1855476	0.81828162	4.08241733	2.380678018
836207	11.845821	9.57153057	8.86369903	3.88823759	5.59337669	6.833113919
836211	13.474081	9.8735907	11.0483464	4.60755047	9.00787153	3.64934497
836214	0.23452309	0.40920745	0.25634655	0.01192134	0.02456606	0.05730318
836216	4.04925777	7.1340805	7.44026105	2.08014595	2.07347622	1.697468947
836221	0.7796507	0.87556515	0.76938808	0.0898312	0.10181231	0.097154599
836265	7.07414609	11.7088772	10.456101	3.20787124	3.49228325	4.407652193
836327	15.4170358	17.0574138	20.0995085	9.32267687	13.7722428	6.070813893
836330	5.03210279	5.04005687	5.973667	0.83624699	1.48581299	1.447073845
836343	9.67170564	9.39183544	5.91088849	3.22896099	3.8108428	3.127693084
836370	1.35583761	1.86191891	0.97360165	0.15817209	0.33318508	0.253432451
836375	0.48221292	0.96534655	0.86060781	0.01389012	0.0343477	0.093473317
836379	3.66043535	4.14918477	2.41071946	0.97508425	0.82859207	1.652533
836388	59.1783138	52.5257761	39.4398845	10.3761894	20.8600104	15.51109217
836391	40.5346421	41.6899843	31.9561655	12.826138	17.3169486	18.89221993
836439	38.0825526	52.5957667	24.1789965	6.00546922	9.22803507	10.63443378
836496	8.51629443	8.59494339	11.7828347	21.7464456	15.9889898	18.39572677
836513	0.54833084	0.96384173	0.85331979	0.12542805	0.39286979	0.080387225
836554	20.3135897	18.0558294	19.9957246	9.48329271	10.5051753	16.43621565
836584	1.42828209	1.9643986	1.35980508	0.52290656	0.51256081	0.720081832
836595	43.5532219	48.1373981	37.6930786	21.4157714	25.5394438	19.22714821
836625	148.905816	103.929327	106.603534	52.2083151	39.6722819	92.93458098
836664	0.90935061	1.15795567	1.58169863	0.10715646	0	0.025753839
836669	8.08254952	9.14972917	5.70045218	3.44267587	4.06386684	3.112144036
836690	4.02223584	3.63363006	4.17115398	0.29066001	1.31770543	0.908138875
836702	117.667059	133.377434	67.0915592	2.69624233	5.02590128	27.12329492
836710	13.6020509	15.1376314	10.0870029	6.29391164	6.78548909	6.59163602
836721	5.13612941	4.08080672	4.44554825	1.27207107	2.20485725	0.834322736
836736	83.4419459	72.4622113	101.621797	202.733947	153.291316	194.8992188
836763	0.95512949	1.44280628	0.83868828	0.12111463	0.2052089	0.446330752
836793	2.00753856	1.95186708	1.90629448	0.83270954	0.86869825	0.475314196
836794	4.65958629	2.51514631	3.64761077	1.08228743	2.00482429	0.693641356
836796	9.30946207	4.97453822	6.00625441	1.89932457	4.14343538	1.89456824
836813	0.91456992	1.33967274	0.6720978	0.02195345	0.12666926	0.042210105
836826	3.01144535	2.52641481	2.50907745	0.0709728	0.17550261	0.10234505
836842	13.7599546	19.7084363	9.73337518	1.85036239	2.95606017	3.887805591
836845	1.08568351	1.38307253	0.80754576	0.14971146	0.16453722	0.244673873
836874	36.5456034	41.2170781	31.1005744	25.5212216	22.5129441	20.78799583
836882	0.27430456	0.66010745	0.25917497	0.01693142	0.06978045	0
837029	6.63418698	4.42739725	10.0537272	26.8368007	24.4625734	18.34024105
837030	6.94711702	4.15748091	10.191725	23.3270979	17.3685323	23.44104807
837048	1.05055116	1.61985228	0.85329355	0.20704946	0.41353443	0.237326415
837068	14.8701945	15.7398618	13.3484853	6.8438584	8.71888355	7.753039496
837095	245.862547	315.263616	197.931469	55.6452144	81.038969	113.8123787
837117	0.35928173	2.30247542	1.15983919	0	0	0
837147	2.43528474	2.84803332	0.04453478	0	0	0
837194	1.05104284	1.16305219	0.53251652	0.22706429	0.16275013	0.38912441
837197	4.42377329	5.71764264	5.3271641	0.58466311	1.3345511	2.183420299
837210	19.5021037	21.4734159	25.8766654	13.2826101	16.0615218	13.66654166
837249	1.87470347	3.36136669	1.60419805	0.02292484	0	0.044077809
837252	38.4560741	25.9477019	56.4721806	7.21598198	14.7329464	1.74153582
837286	32.7341277	28.6025943	34.3374918	13.7407235	21.6884856	16.45530567
837365	95.0204273	79.4723642	97.7806292	50.7388183	56.7912565	64.22689648
837387	35.2383836	22.4373791	22.2536469	10.8950233	15.4412206	14.91977162
837440	22.0254045	38.1351797	19.5006388	3.49243791	4.05256355	2.966309637
837452	635.459812	576.776827	560.296378	107.575467	149.815168	219.880079
837461	11.0197919	8.29569641	9.46658453	4.87665601	6.72799059	5.270064103

837465	2.29023724	2.50609035	5.40120436	16.2215944	12.7592553	9.275281976
837467	5.13339358	5.50314962	3.94083241	1.52935182	2.81976617	2.669663481
837471	10.1913794	9.8127264	20.3701948	34.9482209	25.7437784	27.15481785
837482	32.8686419	20.7568601	30.3170442	10.85184	16.671556	14.51208883
837484	1.31446985	2.20118288	2.86979182	0.49626577	0.83175088	0.524796139
837504	49.278717	47.5986141	29.2933883	9.06185077	13.8022015	9.627006945
837526	3.81828122	3.7842427	5.17356587	0.50546485	1.92696159	0.404942475
837537	12.2089794	18.1003022	11.0731086	3.19623595	2.64443397	6.225990557
837576	13.9550755	16.5614477	9.53904331	1.60790111	6.86603531	1.52429424
837607	1.09197616	1.68572157	0.11562681	0.04880843	0	0
837610	101.329711	110.158158	84.406753	59.6891092	57.9576477	75.77346111
837617	26.2204602	21.8904033	20.4282108	10.7809308	18.3659651	7.251302494
837620	0.86433218	0.324241	0.69714786	5.19194509	2.16231064	5.910750529
837698	1.39583315	2.74221336	0.57408518	0.06385803	0	0.020463404
837752	16.1074923	15.6544065	13.2035912	8.38105316	8.66065419	8.517911179
837758	5.80660054	6.99088486	5.16692105	2.34563351	3.59067281	2.97360743
837760	7.70373297	12.4497676	10.7072565	1.01304756	0.23857897	0.417384842
837761	186.008225	229.303329	98.3990428	19.9603581	41.16766	73.43957371
837787	6.82004224	8.94235685	6.29424928	3.50327459	4.02691805	4.670315156
837792	2.60674518	2.97989291	2.16341706	0.70013712	1.85676675	0.321907855
837793	0.74112533	1.5379462	0.79600807	0.0164216	0.23687753	0.110508866
837800	1.98888102	1.72685656	4.24331412	0.5405553	0.75403208	0.2798198
837807	14.7891727	13.2886607	8.88401195	4.04718499	5.13129725	5.331259654
837831	10.9610118	12.1294866	9.06458021	3.57413213	6.84166675	3.833563171
837841	3.91459672	3.7347406	2.06834222	0.5007424	0.60536339	0.673947431
837865	33.879304	37.2201013	34.1325493	12.9525367	28.1270656	9.073974887
837875	7.06843635	6.88122656	3.99855524	0.73197187	1.90712076	2.264731869
837884	93.5835801	76.1373149	107.71044	215.821029	172.577197	225.6154151
837885	5.56545596	8.05970487	5.63874921	0.56639401	0.76172307	1.089011007
837928	0.36831416	0.70370986	0.36312977	0.02075727	0	0
837931	11.5529463	11.4783201	16.8864147	8.56802137	4.22499979	9.589595543
837940	4.78545647	7.25260185	6.47583599	2.13929384	1.03953493	4.346742776
837980	277.461565	229.916298	313.428768	163.294481	181.519018	95.80067425
837981	10.7415057	14.8512371	21.161105	6.86697558	5.9335069	7.421107574
838014	7.57531049	6.32495008	6.97529486	0.98192904	1.22142237	3.192375585
838035	9.03170366	12.2294721	8.35198462	4.21669121	5.67550852	4.789223505
838049	9.61054601	11.3639459	7.76675714	4.60468621	5.746324	5.23592478
838062	1.66953268	1.8974103	1.57744766	0.51170515	0.83478093	0.491930118
838065	13.0018283	11.7913261	13.1062444	3.76865106	9.66755711	7.736988945
838100	6.17105088	3.48560971	6.40756637	11.3767507	11.5442674	8.491531038
838127	0.42125343	0.76414612	0.20363748	0	0.0628024	0.012207825
838153	21.4110946	37.275533	25.5234696	6.9581925	14.1590294	9.291386519
838210	3.00072798	1.83871227	2.91943376	9.15278361	7.78774715	6.244501926
838222	6.80762711	6.6255154	5.92504597	2.984118	4.18002153	2.07679685
838263	14.4134577	14.5041	12.9808673	6.66130459	8.92936008	8.430691975
838284	17.7404471	25.5506672	15.3949331	5.10038996	5.44655766	9.385322707
838315	8.61040949	10.2972108	8.15050245	2.96528662	3.0382785	4.94911809
838319	3.24666819	2.55621973	4.0730837	7.93752023	6.10906424	6.54066156
838339	17.657088	18.8897203	19.1271431	5.47041741	12.8145997	4.444712605
838346	2.24831772	1.92112793	1.15871407	0.1766255	0	0.113199828
838347	0.08124003	0.39270443	0.58336945	1.4321504	2.46512203	0.992109064
838361	1.60207803	1.00207626	1.41427251	0.3031606	0.82462607	0.233155878
838363	23.8860876	17.895954	21.8090733	14.079371	12.6705369	14.6923282
838405	0.47664506	1.19690618	0.82028973	0	0	0

838421	1.37724664	1.52950153	0.56577522	0.1164273	0.2399193	0.2798198
838443	15.5012219	20.2785726	15.4884932	4.75341491	9.5186572	6.465451732
838446	8.89328354	11.039279	8.35582129	4.3470291	5.87899204	4.767197244
838448	3.93769149	3.99321462	4.27500164	1.97948461	2.95986325	1.863668787
838465	4.59896851	5.2993592	3.70071524	0.88430973	1.90374518	1.780283244
838515	7.1680683	7.9559204	5.92263309	3.1353742	4.83787085	3.234758193
838518	71.5207926	68.6739289	65.5783088	36.2801532	48.4472303	39.36331561
838564	0.04373095	0.03603241	0.14461623	0.75107707	0.81766807	0.354213905
838584	4.64450839	5.46267408	3.43322688	1.31030446	1.91197478	2.706582361
838586	3.47840997	2.79657863	2.94295991	0.24593424	1.08115532	0.413752537
838592	4.53714567	3.59006146	5.39689347	13.7927012	11.7151945	14.43756729
838596	4.57467399	4.86810565	3.54263402	22.9285479	16.7717373	13.48033144
838608	36.6189946	23.5962293	18.6202596	3.17342776	3.9514771	6.858867095
838677	4.13333688	8.85877776	4.74124148	0.48877497	1.62702892	1.409658239
838689	12.1681105	11.7560523	10.3655928	5.53078782	6.7392015	6.48343658
838700	3.54728561	5.05959771	2.91989542	0.5922941	1.16913742	1.258684012
838703	1.97213309	1.83909304	1.09418103	0.17834818	0.31851592	0.428639625
838721	85.6903957	91.564187	128.811698	76.1534342	48.3637464	70.12668064
838770	156.679303	97.6127833	259.126886	532.007222	353.241653	462.2081301
838793	4.55175434	4.81307495	7.9428498	0.0737511	0.44073431	0.38995528
838812	20.7378733	16.8059064	19.2146897	41.6094549	29.5142913	36.64922618
838821	17.4285337	22.0367653	20.4853427	50.4081502	50.2172044	47.11229263
838828	0.93522535	1.16970866	0.47580718	0.0932508	0.18447359	0
838847	34.5666935	34.841693	35.0705015	10.7256522	17.4201979	9.114639025
838883	13.2459568	9.17416557	16.2146766	48.7277862	26.3399386	43.25638245
838896	54.2856225	43.8180668	66.1030095	24.8027298	29.4093417	40.58815972
838898	12.586197	16.2792745	13.3363619	7.83360965	7.74189357	8.432550188
838911	5.97159455	7.88340922	5.74353494	3.78472802	3.74533532	2.775101032
838916	36.3841873	32.0270405	35.1145393	22.0154227	17.0646204	30.44752439
838921	697.418992	1004.35204	783.064201	1754.86179	2060.18049	1309.300026
838956	7.36384349	11.8627158	7.12493408	1.11283973	2.83695539	3.981545777
838985	4.37943918	7.00234753	3.57451194	1.01274978	1.66956187	1.332310736
838999	0.85883311	0.94352161	0.59236811	0.11132391	0.24775508	0.224745684
839028	4.31775694	3.54767892	4.71725872	2.1446466	2.70593077	2.106980101
839030	7.55374324	11.7247487	8.29943669	1.4267373	3.10146216	1.277471065
839077	14.9576969	17.2913711	15.3411836	31.6157029	27.4644685	29.02141732
839104	0.55423457	1.12823174	0.32344022	0.02112975	0.01741665	0.081252732
839134	6.98544577	7.30780411	9.06594374	16.1127776	11.8347231	17.36064132
839138	17.6300164	14.1400765	19.4081271	39.9175558	34.597855	32.118367
839162	14.2670508	16.6356225	12.2482689	2.99368975	3.88052137	5.477479111
839196	17.1437988	22.9503696	13.0495713	3.49562436	8.73664681	3.240515567
839224	2.66144686	3.01249162	2.30890742	0.73178173	1.74062516	1.447203616
839353	0.23662143	0.30154709	0.56786857	1.07349937	1.18823399	0.737152564
839358	1.02059197	0.87659903	1.12209458	0.14431796	0.2379144	0.115617281
839394	1.51882507	1.76364111	1.94367871	0.11214317	0.95517654	0.742686704
839395	31.6429901	39.3534309	27.6983777	5.06234621	16.5453119	4.454071217
839405	59.4785805	83.7793906	58.3343742	15.8243066	27.2679056	20.9870529
839406	0.97298181	1.79579631	0.5332032	0	0	0
839418	79.8156681	88.5069707	93.8843164	39.3216418	31.2309902	44.90083533
839421	1.11838459	0.96156559	1.86836597	6.34495715	5.07410831	3.332647013
839431	0.85440412	1.38598408	1.06585985	0.02595699	0.17116487	0.17467709
839439	7.88712348	8.10148842	7.25419623	4.72788654	4.42181586	3.654141187
839452	12.3340691	16.5779658	6.3640505	1.91103	1.99732238	1.056677279

839453	0.30551731	0.39351367	0.22894212	0	0.04502439	0.013128077
839464	21.7694255	21.2761651	33.5618363	61.246995	60.3725497	45.50902621
839467	0.17311887	0.71041528	0.59655051	0.02640007	0.08704333	0.019034876
839480	1.18537736	0.35793276	0.75321401	0.1886946	0.17405176	0.060467477
839490	7.03964991	5.86524704	7.23169066	0.85737832	2.9278095	2.649357684
839497	1.86685369	3.03491978	2.07995711	1.07704679	0.86471993	1.15644749
839498	2.70443596	2.6389983	2.1461177	0.9955343	0.80313132	1.150510111
839502	2.50927606	4.33797438	1.04703573	0.16510563	0.34022973	0.095235037
839522	35.8671501	42.2700254	31.1176928	15.9652312	20.5926302	20.91420562
839558	8.63384839	7.42091571	17.198414	1.19907765	4.24173618	2.341500788
839570	7.46296937	10.2011375	8.07564059	1.73107802	2.98805152	2.878052239
839591	36.3233773	35.5111604	45.6816771	250.765854	95.4801921	166.3304528
839606	1.2606683	0.73043533	1.50115602	3.13950015	2.2326126	2.937473186
839610	10.6997836	15.1493791	9.10911521	3.50317719	5.6400483	4.825204228
839637	4.09655165	5.31648758	3.04583787	1.20096317	1.27626036	2.242310775
839686	12.6350122	10.6123031	11.013395	27.55427	18.320341	20.40368698
839688	61.2779783	66.4038399	42.7073146	7.38637531	13.2204818	8.216785908
839698	20.1193823	18.1186005	18.69724	43.2912085	27.1492133	43.51111084
839717	17.1830193	12.8867413	24.3464158	47.1454532	34.9569838	38.9905333
839723	2.57678259	5.80987928	3.99170532	0.40780101	0.6402644	0.896094593
839802	1.99207858	1.18603396	1.87005991	9.06261089	3.84488034	6.534287195
839843	101.302334	138.173672	66.4767353	11.2855246	17.4738035	17.1462929
839844	71.0845363	86.9355581	80.5626218	14.4843487	26.099664	33.62780528
839867	9.00382974	10.3029614	6.96539402	3.54608353	3.76392125	4.019083241
839870	2387.27007	3525.31779	2047.12554	219.443967	1301.85561	363.7229369
839877	5.23811133	5.40469115	4.53676409	0.53522879	2.14284474	1.279011047
839904	3.68656196	2.43614399	3.29989768	9.84464648	9.92121205	6.243260988
839905	67.0944203	58.8381472	44.8611142	203.84635	104.019103	217.4556108
839937	3.65743389	4.28551593	3.30620585	2.49269281	1.8140792	2.09302484
839974	4.73350534	2.0869507	3.40580921	1.09998169	1.58045787	0.747021851
840062	4.77384387	2.33622506	4.9410882	11.8696428	14.5134243	8.57172207
840102	1.81955652	2.47807537	2.72798163	0.19492155	0.65874005	0.730816047
840139	13.2301729	5.85552659	13.2417826	23.3566501	28.4736354	17.45300241
840168	4.34174726	2.73330302	5.59261205	11.6773073	9.72951818	7.011204988
840213	5.91684581	9.24017022	5.14768377	0.78105749	3.77340411	1.001164311
840240	16.9066889	19.0645876	16.0751089	7.61543703	9.71148555	9.644537364
840242	2.86998371	2.4763204	1.53538409	0.2038432	0.2660351	0.342939808
840255	3.93203547	4.70128679	7.54674844	2.30880419	2.15443227	2.931520217
840303	18.4878389	16.1987891	40.9231458	84.6463435	51.8752063	75.06170823
840304	1.75040391	3.14370189	2.09968102	0.51297175	1.18717112	0.701787207
840345	2.53465972	2.0638796	1.9553308	1.04362426	1.26167051	0.980997617
840399	22.6327062	22.5164692	30.1249904	1.4472108	6.93583141	0.695641403
840415	3.77813784	6.49798532	8.69049542	0.06754908	2.19931237	0.422101055
840475	64.5313129	65.19692	68.786826	20.111426	31.524768	14.15669866
840476	112.937079	98.2006358	94.7126028	40.1808189	48.5123726	58.37202082
840543	93.7945087	98.1598954	112.036471	50.0455287	47.6233137	49.98102892
840603	1.6464953	1.35144281	1.32322286	0.22078188	0.18198424	0.613165737
840632	0.470058	0.36648441	1.11749191	2.27996094	2.34913395	1.606097716

840792	18.2102761	22.7646983	9.50827076	1.55405967	2.68297094	2.458633966
840948	0.07112116	0.01302239	0.11199732	0.72214617	0.75988675	0.295420667
840990	3.15744377	2.2194975	1.90884921	0.98790534	1.2408417	0.798976996
841056	1.53285241	2.43402424	1.70388959	0.25880028	0.16515231	0.393262697
841066	4.64147276	6.71328628	2.87106108	0.62760829	1.05247806	0.540938194
841080	4.8788237	5.40518821	4.01793727	0.35252265	0.93336027	1.150203616
841102	45.283376	47.0042468	33.8355837	14.7441947	24.0896635	14.72747005
841124	1.80112146	3.36112682	1.86496275	0.70359459	0.65903758	0.784013626
841132	4.6303889	5.04744981	3.9216271	1.91255944	2.08914122	2.003079795
841141	5.31020981	6.7897263	5.25462462	1.83707433	3.87187536	1.889034837
841248	1.30400254	1.46442397	1.98957904	0.36309673	0.58826032	0.493505293
841269	7.15447809	15.9808221	5.5439574	0.37730655	0.59745331	1.097721601
841336	27.1102436	34.852085	41.1672421	14.196092	8.66090551	17.71754379
841367	11.6681253	14.669124	7.91591176	2.48869701	4.83002599	2.501271315
841382	3.83936642	5.13045675	2.57856014	0.78000857	1.48509842	1.267377213
841396	12.1869408	17.7144164	11.5691992	2.29050144	3.68364321	3.781668459
841398	68.7634358	94.7688348	56.8701759	37.8934644	31.0044584	28.2578259
841407	5.5640186	6.9763237	6.50332399	2.94375834	5.03956359	3.058572284
841425	37.684479	61.6740047	37.6910285	7.18044851	12.6553037	12.80975635
841497	2.74774419	1.94811191	2.33958906	0.5591023	0.93877296	0.278701416
841532	14.5118032	10.9037706	16.0603506	31.7755577	21.7646366	25.26419312
841550	24.3304923	28.4676729	30.7364969	58.8055549	52.7187801	47.34996826
841575	79.3127836	82.8383974	81.2059366	46.8314535	41.9862447	62.42199108
841649	31.2197922	36.1488017	28.2564844	6.11329166	15.6029438	12.52019677
841665	19.2254796	22.8273019	15.4698188	8.62043828	10.9469259	9.843795881
841708	0.22073543	0.34509884	0.08021558	0	0	0.010579423
841710	0.61338033	1.17620077	0.55847413	0	0	0.027794601
841717	2.26201809	2.28011849	2.88639436	0	0.39865237	0.319654124
841741	1.13983154	1.60285307	1.10568181	0	0.34099557	0.056815123
841787	6.19974067	13.061266	4.77237331	0.28363219	0.52602744	0.840735951
841815	92.241012	82.0921404	118.519375	244.966327	173.726913	159.2074728
841821	64.9177484	61.5983439	76.5145561	25.7073247	51.9965558	34.94606285
841887	1.08565214	1.61015443	0.94883885	0.16417883	0.14499423	0.112738625
841921	17.6356593	26.2463366	15.2119032	4.04725574	8.55199361	5.348331837
841970	8.51853031	17.5603921	5.35372927	0.66995879	1.22717342	0.930320428
841982	44.8679824	50.4640917	35.8772209	25.2071129	29.3739537	23.17487884
841992	119.317808	103.380932	133.367087	63.9666129	72.1024281	81.2845159
842000	41.8126055	42.47096	50.3148332	24.4691587	30.2659281	17.66378316
842015	0.02526555	0.02775695	0.11935997	1.03161797	0.33743391	0.613940915
842105	7.93803507	7.58935467	9.4663247	4.47040511	5.20841718	4.462179279
842107	1.63875173	2.66718084	2.5649674	0.68660603	1.3559216	0.75633237
842108	0.82507072	0.45321498	0.86618109	0.14853826	0	0.071398974
842128	8.64737584	8.00118115	6.50002281	0.94653153	2.28926842	1.281117288
842157	1.39869582	2.43219533	1.7674706	0.21133379	0.3208882	0.363561492
842166	76.0571922	78.1213393	125.6343	28.149808	37.4536235	36.79299048
842183	4.0878504	3.49295987	2.38418533	0.19625056	0.8627401	0.943331903
842190	32.1788211	10.500575	29.8566346	4.76803525	6.56057584	3.392713695
842192	19.5528438	16.8656609	18.4253802	34.7567204	32.1215828	38.32538824
842196	20.8758943	9.00869564	33.759757	0.94604994	5.65356556	2.246974036
842198	188.461322	157.262437	172.265241	56.2603044	145.616791	56.02583281
842208	0.32200795	0.50429726	0.09710018	0.00888072	0	0.008537526

842213	16.8491486	15.9295486	13.890538	7.61990807	8.57560181	10.17770404
842257	0.1155205	0.02538237	0.37110603	1.13802635	1.70328834	0.806139818
842285	23.2695978	17.4319351	23.680449	10.0333245	17.2951371	10.96225769
842291	2.12499227	2.47349563	2.4858301	0.98373696	0.8784387	0.929958083
842300	2.3301592	2.26153625	2.49878995	0.44472229	0.38184581	0.498791804
842334	0.46775464	0.60456396	0.45062088	0.13077366	0.05879619	0
842373	5.98662289	5.67615229	3.70561092	1.80554392	2.3537708	3.397673542
842410	4.06760406	8.58656241	4.46261745	1.28544939	1.05955949	1.570897533
842542	1.39666779	1.13451979	0.79177977	0.02194415	0.38889079	0.210961137
842549	2.63098693	3.02441861	2.42002087	0.29360589	0.22339478	0.4342452
842578	56.694668	41.3345298	43.5499994	23.8983424	23.0319711	24.75781221
842580	18.4589638	8.07668277	20.7697688	47.2174147	33.1705954	35.66095255
842588	0.34624127	0.57057557	0.10468606	0.01795223	0.01479752	0
842613	0.1056013	0.24653085	0.15590115	0	0.00705179	0.008224558
842632	9.31584345	9.03635641	5.41280609	1.51581297	1.23540299	2.161290608
842658	0.55486495	0.18505092	0.31830115	2.11915573	1.58796363	0.765515944
842743	123.109018	72.3357922	144.054504	333.353529	411.540777	293.379963
842748	18.735981	22.8951296	18.3045429	8.13880032	10.0583952	9.829961601
842772	8.46403016	8.14419706	5.09075993	1.38688729	2.22510241	1.880891985
842775	3.7097313	5.40506524	3.70815705	0.35185159	0.54378992	0.732884004
842806	10.6604039	16.7207976	12.8514331	4.22872021	5.92828869	5.249678413
842809	10.1331447	11.7872067	6.39736868	2.7912839	4.76749529	3.530807499
842857	4.01175062	1.22619034	0.51733556	0	0	0
842890	10.4141976	6.29007098	10.0517918	6.1177293	5.96027157	3.972292852
842904	1.77466754	1.47797213	1.01688817	0.44523185	0.91136345	0.663439842
842922	22.437971	20.2110345	30.5303279	6.42021882	10.953609	3.644482277
842923	56.6242981	48.505354	107.534306	1.84814373	16.6674871	2.418730144
842933	35.4592155	33.9234823	57.4611134	6.3293373	26.4208511	10.69175342
842961	25.299831	18.2764603	17.2203323	5.04889409	11.6163476	9.858763432
843012	12.8829225	21.9103846	9.09024844	3.12216794	2.19116379	4.356478241
843014	2.96101707	1.49462134	2.77707686	1.49941997	0.76176779	0.126922268
843025	21.7110277	19.9265044	17.23766	7.47714619	6.08231773	12.45198111
843038	0.3003657	0.56568754	0.25339168	0	0.02292305	0.013367666
843052	4.07283993	5.11942466	4.56467895	1.53781647	1.68574876	3.048220591
843086	1.7197534	2.21114623	1.36598601	0.23271697	0.43412348	0.188398769
843100	2.02843233	2.97127146	0.70168836	0.07183881	0.23685876	0.027625027
843109	5.17546754	6.3630718	5.06610308	1.87690991	2.83376704	2.000892231
843113	167.894177	144.994889	175.771585	344.759764	311.025282	341.2993503
843123	5.06503605	6.63116916	4.73979905	0.86000649	1.22757089	1.794698493
843134	12.2425675	13.5136869	11.347508	6.87912098	6.34603564	7.881574077
843162	0.43470622	0.41473364	0.54043354	0	0.07333538	0.028510546
843176	0.69542945	1.29004108	0.76478899	0.14777566	0.04872292	0.014206482
843190	21.5412994	43.5002986	23.898914	64.5264864	59.1017366	56.57090286
843209	3.92447165	4.67926127	2.82055005	1.07284855	0.90419097	2.514732342
843240	1.75868408	3.06404658	2.08129843	0	0.20878311	0.33205283
843274	13.841206	17.1786573	10.5477849	4.27028944	8.07403415	5.058617328
843305	7.34302722	7.04542172	5.25385228	3.25226079	3.1879146	4.164744047
843306	7.62905511	9.97549375	6.52275338	1.42492153	3.6410195	1.64382589
843313	2.20317863	3.22962891	1.73676937	0.63368575	1.27448324	0.414253775
843345	14.0801255	14.4493042	23.3481929	2.75886909	7.54520643	2.244212871
843357	3.89174088	4.58214528	2.32831967	0.21708162	0.50271997	0.258381093
843373	70.2225308	86.2434408	55.6014217	8.66060431	33.642209	13.89057225

843420	203.412494	513.090962	345.212875	3.75146307	25.6526566	27.69952268
843422	0.01555257	0.01708619	0.05877898	1.29021377	0.24925468	1.395397105
843428	2.03030459	2.19020005	2.60013358	0.7926889	0.81020483	1.143082722
843432	37.550965	37.1276403	43.6135877	13.9459883	23.254468	16.99190734
843469	0.23389521	0.07137759	0.04910988	1.1285045	0.44427189	0.696274627
843481	0.44870204	1.02501887	0.53162405	3.06503363	2.07744804	1.677422973
843492	0.26676369	0.02442241	0.18903752	1.70011049	2.70769878	1.772918334
843556	5.83707892	2.62613951	8.71915478	0.03602931	1.75217835	0.381006376
843610	5.45459259	4.57660682	4.57919498	1.92935152	2.24970648	2.047055932
843628	6.9070132	7.13198983	4.04757673	2.31164253	1.7138711	2.081209308
843633	2.9125494	3.19975239	1.99073931	0.79321736	1.41524489	0.955617155
843716	9.07956425	13.0250619	15.0305728	3.02604111	3.78596056	2.742864426
843727	7.9665477	7.80900307	10.7716019	2.75964699	3.88900114	6.033434148
843771	51.1864559	61.8408309	64.4965778	7.10490938	19.0366976	3.536849359
843779	16.418108	14.9245343	15.6088395	8.44127583	9.98497385	10.99787419
843785	5.81430573	6.68841109	7.21806278	1.48704922	3.35683564	1.543298377
843808	13.471952	17.638963	10.1472346	4.80436925	6.40319932	6.583451394
843842	102.881924	51.7915036	150.527403	3.619613	39.3125847	5.594862747
843852	0.45712758	0.67483714	0.51289808	0.00925843	0.02289437	0.026701889
843855	28.0455177	25.6871433	22.929051	6.50817115	18.3681565	9.477000404
843861	1.70832364	1.91534333	1.31560128	0.10616833	0.31254124	1.210204253
843902	16.9663261	23.3273422	12.8525929	3.18831673	3.80269292	5.816729639
843905	5.7478606	6.03624881	4.22277012	1.41843341	2.70756188	3.272682068
843911	0.51016212	0.96254388	0.26196931	0	0.04739804	0
843923	7.47739831	6.28551736	6.12297719	1.65945198	1.92465873	2.004740724
843948	22.533358	38.6766316	16.8295582	5.10182887	6.73219838	6.296678659
843987	1.00875508	1.7346165	1.87860549	0.11370186	0.40612528	0.546539039
844004	7.68845188	5.03547359	8.56825056	23.4135867	11.9796942	21.83319007
844063	1.04555549	1.59170967	0.550394	0.07744417	0.23140198	0.05583848
844094	14.0737663	14.2003978	9.73366562	6.2226286	7.73352699	7.486980066
844099	0.88595261	1.01075054	2.20540192	0.08833785	0.12742517	0
844105	3.37231569	4.12907579	3.27549598	1.16789725	2.31039503	1.015833582
844127	13.3024156	8.27368063	10.8466937	25.2941047	16.8763359	23.37683247
844139	196.784222	215.812689	181.606739	84.917799	118.93719	121.6880522
844185	14.3030836	18.3357795	15.4505751	5.96524564	11.4926279	10.18485652
844193	29.5579769	32.3189973	24.6826587	15.4302381	17.8908717	16.16046383
844214	5.11022195	3.00474819	10.9261518	22.3287427	19.7635322	19.88131438
844219	10.830676	11.7073404	9.36938087	0.46561406	0.8373654	1.763352445
844220	167.839651	129.32115	166.372841	71.5170738	44.4086206	116.4349359
844222	55.3523779	20.5671384	70.0103789	5.20883636	12.2307308	7.979372146
844247	1.2153731	1.33521958	0.87051412	0.31761983	0.27227722	0.54962153
844321	3.52811183	4.06356379	3.38728108	1.22444254	1.05791294	1.347310029
844408	2.40588077	3.34038703	1.17145555	0.13392579	0.44156491	0.404643404
844422	16.777897	15.3360219	20.5857012	38.8347055	34.5496518	27.92062255
Novel0000[illegible]	0.03398817	0.05600956	0.27296489	2.7975717	1.74308715	1.588262897
Novel0004[illegible]	10.6905303	11.2200329	8.4926084	5.22228502	3.84664728	7.935086004
Novel0008[illegible]	9.63715317	5.25402764	7.23895577	2.29538625	2.02073078	2.551943085

Novel0010(	0.52771417	0.87897798	2.10701871	4.83237741	3.27385619	3.860750533
Novel0011(	10.3148627	5.5826759	9.60261181	0.78649179	1.90433098	0.897865811
Novel0011	10.968614	7.57909504	10.7231499	3.70991531	3.74734878	3.339769769
Novel0014	4.0782043	3.89243173	2.10970682	0.57407365	0.70978895	0.252948831
Novel0018	0.83328894	0.86968563	1.39750705	3.08736629	3.36837359	2.050812734
Novel0018	0.06488651	0.08554187	0.13487677	0.62239462	1.09537181	0.646856162
Novel0018(	43.0256907	41.7904213	51.2887091	104.168385	84.5247661	61.55901561
Novel0019	2.9680762	1.97819113	2.92589113	7.16878021	6.15214843	4.142441242
Novel0023	132.640479	88.1237547	156.817341	226.092738	232.650648	262.4724101

log_2FoldChange NSs vs WT	FDR NSs vs WT	Gene name	Description
-1.1166	0.014668	AT4G22305	alpha/beta-Hydrolases superfamily protein
-1.938	0.00011295	CDT1	cadmium tolerance 1
1.1113	0.0071638	AT1G76955	Expressed protein
1.0519	0.0065883	AT3G05937	hypothetical protein
-2.515	0.027659	PRE5	basic helix-loop-helix (bHLH) DNA-binding family protein
1.3529	0.0004694	AT5G35732	hypothetical protein
-1.942	0.020102	AT1G05347	other RNA
-2.5044	0.0065118	AT1G11303	G-type lectin S-receptor-like Serine/Threonine-kinase
1.6642	0.043341	AT2G13275	hypothetical protein
-3.445	0.0087857	CLE42	CLAVATA3/ESR-RELATED 42
-1.2667	0.00026199	RALFL22	ralf-like 22
-2.7961	0.00018614	AT3G29631	nuclease
2.17	0.041129	AT3G48835	polynucleotide adenylyltransferase domain/RNA recognition motif protein
-1.9833	3.06E-07	AT3G60415	phosphoglycerate mutase family protein
-3.0505	0.041751	RTFL14	ROTUNDIFOLIA like 14
-1.3382	1.73E-05	_IG005I10.1(	receptor-like kinase
-4.3523	0.0016953	AT4G13577	hypothetical protein
-3.0354	4.74E-05	AT4G18253	receptor Serine/Threonine kinase-like protein
3.5867	0.010042	AT4G38213	tubulin-tyrosine ligase
-2.3421	0.037516	AT5G03355	cysteine/histidine-rich C1 domain protein
-3.0548	1.23E-05	AT5G51465	hypothetical protein
1.4727	0.040592	AT5G63625	hypothetical protein
-3.2908	2.13E-05	GRP23	glycine-rich protein 23
-2.0289	2.47E-11	AT3G23450	transmembrane protein
-1.7889	0.02682	i43.TRNA-TR	tRNA-Trp
-2.4023	1.13E-10	AT1G24147	transmembrane protein
-1.4243	0.013882	AtEWR1	transmembrane protein
-1.4876	8.57E-05	AT5G08760	transmembrane protein
-2.8047	2.54E-10	AT5G44568	transmembrane protein
-1.537	0.0064233	AT1G65486	transmembrane protein
-1.7712	0.00018749	TCL2	Homeodomain-like superfamily protein
-5.2211	0.020904	AT4G16807	ATP-dependent caseinolytic protease/crotonase family protein
1.4359	0.042219	AT1G01355	Putative endonuclease or glycosyl hydrolase
-1.1699	0.013476	RHA2B	RING-H2 finger protein 2B
-1.4905	0.0036949	PAP1	phosphatidic acid phosphatase 1
-1.0597	0.037076	RR14	response regulator 14
-1.1726	0.00019914	ATMAP65-6	Microtubule associated protein (MAP65/ASE1) family
-2.8698	1.16E-07	T20F6.8	Leucine-rich repeat protein kinase family protein
-1.1571	0.012753	MYB88	myb domain protein 88
-3.1677	0.0022609	EXPA15	expansin A15
-3.0018	0.033389	ZIP7	zinc transporter 7 precursor
-2.367	0.020904	AT2G04495	transmembrane protein
-5.4185	0.0018805	FLA7	FASCICLIN-like arabinoogalactan 7
-2.0038	0.00048462	LHCB2.2	photosystem II light harvesting complex protein 2.2
-1.8094	0.0033881	LHCB2.1	photosystem II light harvesting complex protein 2.1
-6.8514	2.93E-11	GRP9	hypothetical protein
-2.7282	1.99E-06	T25M19.1	O-Glycosyl hydrolases family 17 protein
-0.91221	0.027682	T6P5.12	Subtilase family protein
-4.0854	4.40E-11	XTH4	xyloglucan endotransglucosylase/hydrolase 4
-1.2435	0.0091762	PLA2-ALPHA	Phospholipase A2 family protein
-2.4449	8.57E-10	MCM5	Minichromosome maintenance (MCM2/3/5) family protein
-5.8638	1.86E-05	F15K19.1	Bifunctional inhibitor/lipid-transfer protein/seed storage 2S albumin superfamily protein

-1.5895	0.045266	ABCG5	ABC-2 type transporter family protein
-2.9424	0.030492	XYP2	hypothetical protein
-3.3217	0.0054819	PR1	pathogenesis-related protein 1
-2.4927	9.25E-07	AGP9	arabinogalactan protein 9
-4.4515	4.19E-06	T26I20.6	Gibberellin-regulated family protein
-2.4527	1.51E-07	MCM4	Minichromosome maintenance (MCM2/3/5) family protein
-3.8821	0.0099119	T24I21.16	kinase with adenine nucleotide alpha hydrolases-like domain-containing protein
-3.4101	0.0051154	PIP2%3B8	plasma membrane intrinsic protein 2%3B8
-1.201	0.000724	LYM2	lysm domain GPI-anchored protein 2 precursor
-1.8673	0.0030182	HMG2	3-hydroxy-3-methylglutaryl-CoA reductase 2
-0.98315	0.012453	MLO8	Seven transmembrane MLO family protein
-0.74538	0.042003	TRM26	RB1-inducible coiled-coil protein
-2.5633	0.00015099	MCA2	PLAC8 family protein
-3.7093	2.24E-05	GATA20	GATA transcription factor 20
-2.0867	0.030486	SLP3	subtilisin-like serine protease 3
0.83702	0.044536	F6F22.10	tRNA/rRNA methyltransferase (SpoU) family protein
1.16	0.0020114	F6F22.4	RNA-dependent RNA polymerase family protein
#NAME?	0.027185	LLG2	LORELEI-LIKE-GPI ANCHORED PROTEIN 2
-10.002	0.0005512	EXPB1	expansin B1
-3.2947	0.026482	MCM10	minichromosome maintenance 10
-1.9889	0.015527	LAX2	like AUXIN RESISTANT 2
0.98963	0.0076792	F26H11.11	Cyclophilin-like peptidyl-prolyl cis-trans isomerase family protein
-6.3022	0.0034293	PRP2	proline-rich protein 2
#NAME?	0.019115	F7O24.6	SAUR-like auxin-responsive protein family
-2.3868	0.0021612	SFH3	SEC14-like 3
-1.4435	0.0016346	F7D8.15	Cysteine/Histidine-rich C1 domain family protein
-1.5015	0.0013291	SCPL9	serine carboxypeptidase-like 9
-4.2209	0.020208	AGP17	arabinogalactan protein 17
-1.1063	0.011466	MYB70	myb domain protein 70
-1.0573	0.030862	CLF	SET domain-containing protein
3.6389	0.030486	F26B6.19	GDSL-like Lipase/Acylhydrolase superfamily protein
-0.9517	0.011015	F26B6.33	Cold acclimation protein WCOR413 family
-4.859	8.77E-07	F27L4.13	HTH-type transcriptional regulator
-1.4016	0.00030075	AFR	Galactose oxidase/kelch repeat superfamily protein
-1.0525	0.046234	F25P17.15	major centromere autoantigen B-like protein
-1.8359	0.00082304	F25P17.10	Ankyrin repeat family protein
-0.93706	0.049305	F13D4.60	Polynucleotidyl transferase%2C ribonuclease H-like superfamily protein
-2.7354	0.012089	AT2G25220	Protein kinase superfamily protein
-1.6715	0.037968	T22F11.14	transmembrane protein
-2.0257	0.0048131	F13B15.17	transmembrane protein
-1.8227	0.012535	F13B15.19	AFG1-like ATPase family protein
-2.7549	2.54E-10	HSFA2	heat shock transcription factor A2
-1.4128	0.024	T1D16.17	calmodulin-binding family protein
-1.1056	0.00016829	KCS10	3-ketoacyl-CoA synthase 10
1.4886	0.0069282	FAP2	Chalcone-flavanone isomerase family protein
-1.4436	0.00016125	ER	Leucine-rich receptor-like protein kinase family protein
-1.0023	0.0015997	EIL1	ETHYLENE-INSENSITIVE3-like 1
-0.95484	0.038483	T20P8.11	Leucine-rich repeat protein kinase family protein
-1.0169	0.031859	SCPL51	serine carboxypeptidase-like 51
-1.6189	0.0010321	F24D13.13	UDP-Glycosyltransferase superfamily protein
-0.95993	0.028963	FUC1	alpha-L-fucosidase 1
-2.6302	0.012089	F24D13.7	enabled-like protein (DUF1635)
-1.0617	0.0094977	F24D13.6	UPSTREAM OF FLC protein (DUF966)
-1.9861	1.07E-06	BGAL8	beta-galactosidase 8
1.1071	0.00043253	T11P11.3	Histone superfamily protein

-1.9139	0.0084674	EXPA6	expansin A6
-1.9001	0.0003585	RBL1	RHOMBOID-like 1
-1.0047	0.022088	F16P2.32	NAD(P)-binding Rossmann-fold superfamily protein
-1.0873	0.00034568	F16P2.28	NAD-dependent epimerase/dehydratase family protein
-2.1563	4.85E-05	TUB7	tubulin beta-7 chain
0.90375	0.0050763	THIC	thiaminC
0.87351	0.0056246	T27A16.23	Tetratricopeptide repeat (TPR)-like superfamily protein
0.72068	0.044091	F23F1.11	Double Clp-N motif-containing P-loop nucleoside triphosphate hydrolases superfamily protein
-2.9424	0.012532	FAD3	fatty acid desaturase 3
-2.599	0.0091203	TBL45	TRICHOME BIREFRINGENCE-LIKE 45
-0.83002	0.040592	KT1	potassium transporter 1
-1.0708	0.011459	T27E13.11	UDP-Glycosyltransferase superfamily protein
-1.85	0.022036	ETC2	Homeodomain-like superfamily protein
-0.86075	0.011051	C4H	cinnamate-4-hydroxylase
-0.95393	0.0031444	RPT2	Phototropic-responsive NPH3 family protein
0.74306	0.04198	AT2G30610	BTB/POZ domain-containing protein
-1.5206	0.0088726	F7F1.22	Protein kinase superfamily protein
0.84347	0.027659	T16B12.8	trichome birefringence-like protein (DUF828)
-1.8729	0.00066243	T9H9.27	basic helix-loop-helix (bHLH) DNA-binding superfamily protein
-0.80043	0.029388	UGT74D1	UDP-glucosyl transferase 74D1
-0.91865	0.011936	PARG2	poly(ADP-ribose) glycohydrolase 2
-1.5813	0.029303	SOBIR1	Leucine-rich repeat protein kinase family protein
3.0054	0.0099443	RLP22	receptor like protein 22
-3.6652	0.039657	PAP13	purple acid phosphatase 13
-1.0366	0.00075811	AP4.3A	protein kinase family protein
1.4718	0.012089	PHT1%3B5	phosphate transporter 1%3B5
-2.176	0.0099394	PDLP3	plasmodesmata-located protein 3
-1.5966	0.00081623	scpl46	serine carboxypeptidase-like 46
-2.4104	0.0050324	GALS1	glycosyltransferase family protein (DUF23)
0.87002	0.0061848	PHO2	phosphate 2
-2.3179	0.0007735	T14G11.18	Peroxidase superfamily protein
-2.2047	3.11E-06	T14G11.29	hypothetical protein (DUF688)
-2.4768	2.29E-09	F13P17.14	S-adenosyl-L-methionine-dependent methyltransferases superfamily protein
1.789	0.0051957	F13P17.20	MATE efflux family protein
-1.2579	0.0022373	CCP1	P-loop containing nucleoside triphosphate hydrolases superfamily protein
-2.69	0.0034159	T31E10.4	Mitochondrial transcription termination factor family
-2.1136	0.0079316	PID	Protein kinase superfamily protein
-0.82585	0.019173	GlcNAc1pUT2	N-acetylglucosamine-1-phosphate uridylyltransferase 2
-3.648	2.66E-05	NPSN11	Putative plant snare 11
-1.7061	0.00039979	CSLA07	cellulose synthase like
-1.8183	0.028341	PGSIP7	Nucleotide-diphospho-sugar transferases superfamily protein
-4.4673	0.00027712	FLA16	FASCICLIN-like arabinogalactan protein 16 precursor
-2.9102	2.30E-05	OFP15	ovate family protein 15
-3.6343	4.74E-05	F2H17.19	P-loop containing nucleoside triphosphate hydrolases superfamily protein
0.94094	0.0047578	ABCG34	pleiotropic drug resistance 6
0.75474	0.032894	SBE2.1	starch branching enzyme 2.1
-1.4096	0.012189	TRM27	nucleolin-like protein
-1.6481	0.016213	DML1	demeter-like 1
-2.9134	7.06E-05	F1O11.20	Leucine-rich repeat protein kinase family protein
-1.2881	0.0011313	F13K3.3	Sulfite exporter TauE/SafE family protein
-3.114	0.0021008	XTH32	xyloglucan endotransglucosylase/hydrolase 32
-1.0754	0.029075	MAT3	methionine adenosyltransferase 3

-0.95627	0.015242	AT2G36895	D-tagatose-1%2C6-bisphosphate aldolase subunit
-4.5928	3.69E-07	TRFL8	TRF-like 8
-1.2204	0.02065	PAL1	PHE ammonia lyase 1
-0.88878	0.032328	RIP2	ROP interactive partner 3
1.4064	0.0010798	T2N18.11	Peroxidase superfamily protein
-3.0458	0.032104	MAKR3	membrane-associated kinase regulator
-1.34	0.0052112	\ transferase	Core-2/I-branching beta-1%2C6-N-acetylglucosaminyltransferase family protein
-2.4214	0.0027922	EXP3	Barwin-like endoglucanases superfamily protein
0.85338	0.023865	DET2	3-oxo-5-alpha-steroid 4-dehydrogenase family protein
2.3543	0.0075952	F16M14.3	proton-dependent oligopeptide transport (POT) family protein
-0.91519	0.0075797	AUX1	Transmembrane amino acid transporter family protein
-1.6093	9.50E-09	LP1	lipid transfer protein 1
-1.1764	0.0085393	T6A23.22	Mitochondrial ATP synthase D chain-related protein
-1.5866	0.047192	HTA8	histone H2A 8
-1.3047	3.85E-06	PIP2E	plasma membrane intrinsic protein 2E
-0.86345	0.036851	MTP11	Cation efflux family protein
-5.5312	0.0041434	F12L6.22	Putative membrane lipoprotein
-1.2213	0.021286	WLIM2a	GATA type zinc finger transcription factor family protein
1.5902	0.036297	T28M21.8	HAD superfamily%2C subfamily IIIB acid phosphatase
0.91406	0.047192	SMAX1-like 8	Double Clp-N motif-containing P-loop nucleoside triphosphate hydrolases superfamily protein
-3.6347	0.016282	TBL28	TRICHOME BIREFRINGENCE-LIKE 28
1.6183	0.027185	DREB2C	Integrase-type DNA-binding superfamily protein
-1.2642	0.00013339	T2P4.13	Nucleotidyltransferase family protein
-1.4803	0.0044969	ETG1	E2F target protein 1 (ETG1)
-1.5312	0.0024058	TCH3	Calcium-binding EF hand family protein
-0.86229	0.032282	CAM2	calmodulin 2
-2.5534	3.53E-06	T3K9.6	F-box family protein
-1.7936	0.0071638	F13H10.12	Glutaredoxin family protein
-4.0977	4.74E-06	BZIP34	Basic-leucine zipper (bZIP) transcription factor family
-1.157	0.0071513	COR15A	cold-regulated 15a
-1.2448	0.022608	LSH10	LIGHT-DEPENDENT SHORT HYPOCOTYLS-like protein (DUF640)
-3.6295	5.82E-06	PAR1	phy rapidly regulated 1
-0.97671	0.016396	F14B2.9	Proline-rich extensin-like family protein
-1.0999	0.00034239	F6E13.19	Late embryogenesis abundant protein%2C group 2
-0.85482	0.013779	MTHFR2	methylenetetrahydrofolate reductase 2
-1.018	0.0051365	F4I1.2	carboxyl-terminal peptidase (DUF239)
-1.6203	0.0050773	F4I1.11	Bifunctional inhibitor/lipid-transfer protein/seed storage 2S albumin superfamily protein
-3.0868	0.019972	F16B22.7	zinc ion binding protein
-2.3461	0.0048445	CYCP4%3B1	cyclin p4%3B1
-0.84686	0.049142	AFO	Plant-specific transcription factor YABBY family protein
-2.287	1.96E-05	BEN1	NAD(P)-binding Rossmann-fold superfamily protein
1.0359	0.033389	DTA2	downstream target of AGL15 2
-1.1862	0.00029276	AGP16	arabinogalactan protein 16
-1.8806	0.00016691	AT2G46535	hypothetical protein
1.2168	0.00010502	SAUR32	SAUR-like auxin-responsive protein family
-1.5382	1.79E-05	ROPGAP3	Rho GTPase activating protein with PAK-box/P21-Rho-binding domain-containing protein
-2.1174	0.0061848	F19D11.6	RNA-binding (RRM/RBD/RNP motifs) family protein
-1.5302	3.23E-05	F14M4.24	Pectinacetylesterase family protein
-2.3668	4.48E-08	F14M4.16	calcium/calcium/calmodulin-dependent Serine/Threonine-kinase
-1.3063	2.32E-05	PTI1-4	Protein kinase superfamily protein
-1.0164	0.0096121	SDR3	NAD(P)-binding Rossmann-fold superfamily protein

-1.543	0.0022373	BOR1	HCO3- transporter family
-1.2167	0.016396	T30B22.26	Tetratricopeptide repeat (TPR)-like superfamily protein
-1.771	0.0072305	T30B22.20	P-loop nucleoside triphosphate hydrolases superfamily protein with CH (Calponin Homology) domain-containing protein
-1.1993	0.0069851	GSTF8	glutathione S-transferase phi 8
1.1163	0.0026383	F17A22.28	B-box type zinc finger protein with CCT domain-containing protein
1.5667	0.0033881	NAC048	NAC domain containing protein 48
-2.0727	1.77E-05	WDL1	WVD2-like 1
-0.86936	0.041379	NEK2	NIMA-related kinase 2
-1.3504	0.049107	F22F7.12	Tudor/PWWP/MBT superfamily protein
0.93867	0.014681	PLDP2	phospholipase D P2
0.85641	0.012453	NF-YA2	nuclear factor Y%2C subunit A2
-3.0969	0.0008787	RECQI1	RECQ helicase l1
4.3861	0.019564	INVH	invertase H
-0.80407	0.031713	F2O10.14	neurofilament protein-like protein
-1.5166	4.20E-06	F2O10.13	Pectinacetylesterase family protein
-2.2048	0.036079	LUL4	RING/U-box superfamily protein
-1.52	0.032118	NHX4	sodium hydrogen exchanger 4
0.7561	0.027659	A/N-InvC	Plant neutral invertase family protein
-0.85606	0.018681	RWA2	O-acetyltransferase family protein
-1.7155	0.049642	AT3G06770	Pectin lyase-like superfamily protein
-4.0842	0.012738	AT3G06880	Transducin/WD40 repeat-like superfamily protein
-1.9253	0.011803	AT3G06890	transmembrane protein
-1.9094	0.0017609	AT3G07010	Pectin lyase-like superfamily protein
-1.2918	3.55E-05	AT3G07090	PPPDE putative thiol peptidase family protein
-1.9379	0.00011295	AT3G07195	RPM1-interacting protein 4 (RIN4) family protein
-2.5405	7.32E-06	T1B9.1	O-Glycosyl hydrolases family 17 protein
-1.8703	5.10E-07	CIB3	basic helix-loop-helix (bHLH) DNA-binding superfamily protein
-1.4343	3.84E-05	AT3G07350	sulfate/thiosulfate import ATP-binding protein%2C putative (DUF506)
-0.79497	0.029089	AT3G07470	transmembrane protein%2C putative (Protein of unknown function%2C DUF538)
-1.1763	0.015489	AT3G07540	Actin-binding FH2 (formin homology 2) family protein
-0.82702	0.049359	TK1a	Thymidine kinase
-6.5976	0.031651	PIP5K6	Phosphatidylinositol-4-phosphate 5-kinase family protein
-1.0206	0.030492	SCPL27	serine carboxypeptidase-like 27
-1.5932	1.99E-05	AT3G08030	DNA-directed RNA polymerase subunit beta (Protein of unknown function%2C DUF642)
-1.1646	0.037802	AT3G08630	alphavirus core family protein (DUF3411)
-1.0318	0.0043826	AT3G08680	Leucine-rich repeat protein kinase family protein
-1.3923	0.0095904	Fes1A	Fes1A
-1.2832	0.0048295	AT3G09440	Heat shock protein 70 (Hsp 70) family protein
-2.2099	0.0031651	EXO70H4	exocyst subunit exo70 family protein H4
-5.2702	0.034556	SAUR48	SAUR-like auxin-responsive protein family
0.93164	0.012014	SCPL7	serine carboxypeptidase-like 7
-2.5107	5.10E-18	AT3G10720	Plant invertase/pectin methylesterase inhibitor superfamily
-0.82889	0.033429	RLP34	receptor like protein 34
-1.901	0.0070623	FLA18	FASCICLIN-like arabinogalactan protein 18 precursor
-3.8436	0.0074649	ACT11	actin-11
-5.308	0.0069922	AT3G12170	Chaperone DnaJ-domain superfamily protein
-3.1673	0.00029591	scpl16	serine carboxypeptidase-like 16
-3.0789	0.00014642	DRT100	Leucine-rich repeat (LRR) family protein
-4.0565	7.37E-09	AT3G12710	DNA glycosylase superfamily protein
-1.3431	0.037568	MGH6.15	ubiquinone biosynthesis protein (Protein of unknown function%2C DUF547)

-1.8221	1.32E-05	GIP1	GBF-interacting protein 1
1.0953	0.0029347	DJC66	Chaperone DnaJ-domain superfamily protein
-3.487	0.0026051	AGP12	arabinogalactan protein 12
1.7014	0.044388	AT3G13680	F-box and associated interaction domains-containing protein
0.89843	0.017651	NF-YA6	nuclear factor Y%2C subunit A6
-2.4967	4.24E-10	ESM1	GDSL-like lipase/acylhydrolase superfamily protein
-0.79675	0.047435	AT3G14220	GDSL-like Lipase/Acylhydrolase superfamily protein
-2.1241	8.14E-07	AT3G14240	Subtilase family protein
-1.4703	2.54E-06	SRF7	STRUBBELIG-receptor family 7
2.0407	4.36E-05	AT3G14700	SART-1 family
-5.1579	5.66E-06	AT3G14740	RING/FYVE/PHD zinc finger superfamily protein
1.3518	0.028963	EMB3120	hypothetical protein
-0.92514	0.012427	AT3G15480	fiber (DUF1218)
-1.1811	0.00010356	AT3G15530	S-adenosyl-L-methionine-dependent methyltransferases superfamily protein
-1.6597	0.00053525	IAA19	indole-3-acetic acid inducible 19
-0.83949	0.032031	AT3G15570	Phototropic-responsive NPH3 family protein
-1.4019	0.045807	AT3G15760	cytochrome P450 family protein
-0.85414	0.024752	AT3G15770	hypothetical protein
-1.402	4.72E-05	PDX1.2	pyridoxine biosynthesis 1.2
-1.7542	0.0068521	AT3G16175	Thioesterase superfamily protein
-2.9074	0.0084557	DELTA-TIP	delta tonoplast integral protein
-1.8946	0.016282	T2O4.2	GDSL-like Lipase/Acylhydrolase superfamily protein
-0.92733	0.0030288	RALF23	rapid alkalinization factor 23
-1.0865	0.0073226	AT3G16800	Protein phosphatase 2C family protein
-0.76967	0.022825	AT3G16850	Pectin lyase-like superfamily protein
-2.7095	0.00046001	CTL2	chitinase-like protein
-0.87821	0.013963	MTO3	S-adenosylmethionine synthetase family protein
-2.9238	0.0001267	F13E7.24	zinc knuckle (CCHC-type) family protein
-0.99298	0.017799	RBL14	RHOMBOID-like protein 14
-2.2465	0.00045835	RLK902	receptor-like kinase 902
-3.3079	0.016213	GDH3	glutamate dehydrogenase 3
-3.734	0.0056246	PPT2	phosphoenolpyruvate (pep)/phosphate translocator 2
-0.96809	0.0046671	F4P13.4	Protein kinase superfamily protein
-1.7991	0.00030075	T13O15.9	ARM repeat superfamily protein
1.7196	2.86E-05	SRG3	senescence-related gene 3
-3.671	0.0088441	ICME-LIKE2	alpha/beta-Hydrolases superfamily protein
-1.2155	0.0052607	F14P3.10	O-fucosyltransferase family protein
1.5331	0.040831	WRKY45	WRKY DNA-binding protein 45
-0.77034	0.032175	SP1L3	SPIRAL1-like3
-1.4333	0.022244	LNG2	longifolia2
-1.0764	0.0006828	AT3G18050	GPI-anchored protein
-0.7199	0.045301	ASPG1	Eukaryotic aspartyl protease family protein
-3.1181	1.73E-11	PUB29	plant U-box 29
-3.0702	0.0011866	TSK	tetratricopeptide repeat (TPR)-containing protein
-2.7063	0.047838	AT3G18960	AP2/B3-like transcriptional factor family protein
-0.78495	0.049642	AT3G19100	Protein kinase superfamily protein
-2.1908	0.046603	AT3G19300	Protein kinase superfamily protein
-1.3221	0.027997	AT3G19370	filament-like protein (DUF869)
-0.80838	0.045807	AT3G19540	glutamyl-tRNA (Gln) amidotransferase subunit A (DUF620)
-1.2386	0.0056246	SCO3	SNOWY COTYLEDON protein (DUF566)
-2.2748	0.0066771	AT3G19620	Glycosyl hydrolase family protein
-1.318	0.035982	DWF1	cell elongation protein / DWARF1 / DIMINUTO (DIM)
1.0949	0.027659	AT3G20640	basic helix-loop-helix (bHLH) DNA-binding superfamily protein
-1.8413	0.004546	HTA13	histone H2A 13
-0.69472	0.048454	AT3G20820	Leucine-rich repeat (LRR) family protein

2.4127	2.99E-06	AT3G21080	ABC transporter-like protein
-2.7582	0.0039059	MSR1	O-fucosyltransferase family protein
-0.84919	0.028963	UGT71B6	UDP-glucosyl transferase 71B6
-1.2822	0.0014381	CYCP2%3B1	cyclin p2%3B1
-8.3567	0.00023302	AT3G21950	S-adenosyl-L-methionine-dependent methyltransferases superfamily protein
-4.0835	4.06E-09	CWLP	cell wall-plasma membrane linker protein
-3.8047	0.0065118	PCC1	pathogen and circadian controlled 1
-2.7552	0.0042072	AT3G22235	cysteine-rich TM module stress tolerance protein
1.5851	0.007159	AT3G22560	Acyl-CoA N-acyltransferases (NAT) superfamily protein
-3.2271	9.05E-05	NET1A	Kinase interacting (KIP1-like) family protein
-1.2693	0.010769	IAA7	indole-3-acetic acid 7
-0.84018	0.013599	AT3G23080	Polyketide cyclase/dehydrase and lipid transport superfamily protein
-2.0531	0.015857	WDL3	TPX2 (targeting protein for Xklp2) protein family
-1.4349	0.00034239	RLP37	receptor like protein 37
-1.5776	0.019938	RLP38	receptor like protein 38
-1.757	0.00046001	LSH4	LIGHT-DEPENDENT SHORT HYPOCOTYLS-like protein (DUF640)
-4.6054	0.0020114	AT3G23500	Cyclopropane-fatty-acyl-phospholipid synthase
-3.5132	0.0070496	AT3G23530	Cyclopropane-fatty-acyl-phospholipid synthase
-3.9447	5.90E-05	AT3G23740	hypothetical protein
1.4613	0.0047631	AAE16	AMP-dependent synthetase and ligase family protein
-2.774	0.0010415	RALFL24	ralf-like 24
-1.3489	0.0010113	GAE6	UDP-D-glucuronate 4-epimerase 6
1.2425	0.045077	AT3G23870	magnesium transporter NIPA (DUF803)
-2.8144	0.0041805	TOPII	topoisomerase II
-1.5732	0.0062602	AT3G24480	Leucine-rich repeat (LRR) family protein
-7.0277	0.00016332	CDC45	cell division cycle 45
-2.801	0.00493	AT3G25130	acidic leucine-rich nuclear phosphoprotein 32 family B protein
-1.796	3.68E-05	AFH1	formin homology 1
-1.0118	0.020904	NIK2	NSP-interacting kinase 2
-2.4629	0.00054586	AT3G25670	Leucine-rich repeat (LRR) family protein
0.89246	0.021003	RTFL16	ROTUNDIFOLIA like 16
-3.017	0.024123	HMT-1	Homocysteine S-methyltransferase family protein
-2.516	0.0021797	NUDX13	nudix hydrolase homolog 13
-1.6082	0.0074644	AT3G26700	Protein kinase superfamily protein
-1.1604	0.012529	AT3G27050	plant/protein
-2.9648	0.0046671	LHCB2.3	photosystem II light harvesting complex protein 2.3
-1.2432	0.0066963	KLCR2	Tetratricopeptide repeat (TPR)-like superfamily protein
-3.6948	0.044765	AT3G27970	Exonuclease family protein
1.9824	0.0016022	AT3G27980	Plant invertase/pectin methylesterase inhibitor superfamily
-0.8857	0.024123	UMAMIT44	nodulin MtN21 /EamA-like transporter family protein
-3.9947	8.82E-21	CSLC04	Cellulose-synthase-like C4
-2.9776	0.024765	GATL10	galacturonosyltransferase-like 10
1.3783	0.013465	ABCB15	ABC transporter family protein
-4.4044	0.015625	AT3G28420	Putative membrane lipoprotein
-1.442	9.50E-07	ABCB19	hypothetical protein
-3.0573	0.029948	EXPA5	hypothetical protein
-0.69826	0.04145	AT3G29240	PPR containing protein (DUF179)
-1.667	0.00062681	AT3G42660	transducin family protein / WD-40 repeat family protein
-2.1216	0.0012338	LTPG2	hypothetical protein
1.0053	0.029603	ZIFL2	zinc induced facilitator-like 2
2.6397	0.0061848	AT3G43930	BRCT domain-containing DNA repair protein
-2.8831	0.0002164	NAC061	NAC domain containing protein 61
-2.0571	5.52E-05	PSK3	PHYTOSULFOKINE 3 PRECURSOR
-1.6885	0.0076918	LAS1	lanosterol synthase 1

-4.4627	0.038839	:ctin receptor	Concanavalin A-like lectin protein kinase family protein
-3.1643	0.020308	DOF6	Dof-type zinc finger DNA-binding family protein
-1.1708	0.044387	AT3G45850	P-loop containing nucleoside triphosphate hydrolases superfamily protein
-1.8016	8.09E-08	EXLA1	expansin-like A1
-2.5033	0.0019694	DUT1	DUTP-PYROPHOSPHATASE-LIKE 1
-1.0725	0.0086043	AT3G47010	Glycosyl hydrolase family protein
-1.0927	0.043461	AT3G47540	Chitinase family protein
-1.497	0.0020114	AT3G48080	alpha/beta-Hydrolases superfamily protein
-3.5974	0.031812	WNK3	with no lysine (K) kinase 3
1.1364	0.00044985	CYP71A25	cytochrome P450%2C family 71%2C subfamily A%2C polypeptide 25
-1.058	0.016396	CEP3	Cysteine proteinases superfamily protein
-2.8668	0.017799	AT3G48490	hypothetical protein
-1.0372	0.030874	NPC6	non-specific phospholipase C6
-3.3256	0.04057	AT3G48630	hypothetical protein
-2.0462	0.026482	DCF	HXXXD-type acyl-transferase family protein
-2.0929	0.022422	AT3G48970	Heavy metal transport/detoxification superfamily protein
-0.90834	0.0072199	AT3G49720	transmembrane protein
-1.1279	0.008089	AT3G49810	ARM repeat superfamily protein
-1.185	0.0017276	LBD38	LOB domain-containing protein 38
-3.0563	0.025311	MYB77	myb domain protein 77
-1.8143	0.029075	KICP-02	ATP binding microtubule motor family protein
4.9968	4.08E-05	MAPKKK20	mitogen-activated protein kinase kinase kinase 20
-1.3333	0.00026534	KRP2	KIP-related protein 2
-0.70597	0.042555	ZAR1	HOPZ-ACTIVATED RESISTANCE 1
-2.0851	5.45E-06	GATA6	GATA transcription factor 6
-5.7798	0.033579	AT3G51220	WEB family protein (DUF827)
-1.5886	0.023765	IMK2	inflorescence meristem receptor-like kinase 2
-0.97017	0.0032448	CAM9	calmodulin 9
-1.1805	0.013605	AT3G52360	transmembrane protein
-1.0933	0.0008775	AT3G52470	Late embryogenesis abundant (LEA) hydroxyproline-rich glycoprotein family
-3.9678	0.035074	:ation Proteir	Nucleic acid-binding%2C OB-fold-like protein
-1.387	0.0095641	AT3G52870	IQ calmodulin-binding motif family protein
-2.1523	0.048652	AT3G53190	Pectin lyase-like superfamily protein
-5.717	0.041384	AT3G53980	Bifunctional inhibitor/lipid-transfer protein/seed storage 2S albumin superfamily protein
-1.4161	0.011616	AT3G54250	GHMP kinase family protein
-2.3437	5.06E-05	T14E10.1	Eukaryotic aspartyl protease family protein
-3.2006	0.0018294	HTA11	histone H2A 11
-1.1805	0.032767	SETH3	Sugar isomerase (SIS) family protein
-1.5765	0.017419	AT3G54750	downstream neighbor of Son
-0.97998	0.006244	PMR6	Pectin lyase-like superfamily protein
-1.4282	0.0057655	TON1B	tonneau 1b (TON1b)
-0.90583	0.044091	AT3G55240	Plant protein 1589 of unknown function
-1.2601	0.028317	AT3G55420	hypothetical protein
1.9812	6.31E-05	AT3G55890	Yippee family putative zinc-binding protein
1.629	0.0030182	AT3G55910	ADP-ribosylation factor GTPase-activating protein
-1.3072	0.047192	CCR3	CRINKLY4 related 3
-1.2782	2.83E-05	AT3G56060	Glucose-methanol-choline (GMC) oxidoreductase family protein
-2.3661	0.012116	AT3G56370	Leucine-rich repeat protein kinase family protein
-1.9077	1.38E-09	AT3G56480	myosin heavy chain-like protein
0.80049	0.042413	EDA7	embryo sac development arrest 7
-0.90811	0.029371	AT3G57030	Calcium-dependent phosphotriesterase superfamily protein
1.1538	0.0029347	AT3G57680	Peptidase S41 family protein
0.91226	0.031713	AT3G57770	Protein kinase superfamily protein

-2.0672	1.77E-05	AT3G57780	nucleolar-like protein
0.91795	0.041379	AT3G57940	GNAT acetyltransferase (DUF699)
-5.441	2.27E-06	BZIP61	Basic-leucine zipper (bZIP) transcription factor family
-1.4493	0.0014254	PME61	pectin methylesterase 61
-1.2481	0.0044395	BGLU16	beta glucosidase 16
-2.046	0.020904	AT3G60540	Preprotein translocase Sec%2C Sec61-beta subunit protein
-3.1965	0.027659	MAP65-4	microtubule-associated protein 65-4
#NAME?	0.04695	AT3G61090	Putative endonuclease or glycosyl hydrolase
-0.83556	0.011592	SLD1	Fatty acid/sphingolipid desaturase
2.4908	0.015544	AT3G62040	Haloacid dehalogenase-like hydrolase (HAD) superfamily protein
-1.6132	0.0082501	XT1	xylosyltransferase 1
2.4252	0.00033589	AT3G62960	Thioredoxin superfamily protein
-5.869	0.030581	PLP9	PATATIN-like protein 9
-2.5225	0.016396	CRK37	cysteine-rich RLK (RECEPTOR-like protein kinase) 37
-0.88339	0.009968	CRK40	cysteine-rich RLK (RECEPTOR-like protein kinase) 40
-1.1383	0.0063221	T4B21.10	Major facilitator superfamily protein
-1.7491	0.026347	MSRB6	methionine sulfoxide reductase B6
-6.324	3.42E-05	ACS11	1-aminocyclopropane-1-carboxylate synthase 11
-1.5996	0.0010798	SAH7	Pollen Ole e 1 allergen and extensin family protein
-3.5138	1.45E-05	EXO	Phosphate-responsive 1 family protein
-3.2283	0.020797	AT4G10955	alpha/beta-Hydrolases superfamily protein
-2.6202	1.69E-09	T22B4.2	Ankyrin repeat family protein
-1.4736	0.015242	F25E4.70	bromo-adjacent domain protein%2C putative (DUF3527)
-1.6693	1.81E-05	AT-HSFB2B	winged-helix DNA-binding transcription factor family protein
1.0372	0.020904	AHL1	AT-hook motif nuclear-localized protein 1
1.5095	0.0091197	T4C9.130	Copper amine oxidase family protein
0.77477	0.041379	CYP706A4	cytochrome P450%2C family 706%2C subfamily A%2C polypeptide 4
-1.7712	0.0022112	SKU5	hypothetical protein
-2.6411	0.026176	MYB55	myb domain protein 55
-3.852	0.039949	ORC1B	origin of replication complex 1B
-1.1064	0.031975	T20K18.50	calcium ion-binding protein
-6.6184	0.015232	FLA2	FASCICLIN-like arabinogalactan 2
-1.4251	5.88E-06	ENODL19	early nodulin-like protein 19
-2.075	0.032767	STOMAGEN	stomagen
-1.8752	4.87E-06	LRX3	Leucine-rich repeat (LRR) family protein
-1.562	1.45E-05	CER26	HXXXD-type acyl-transferase family protein
-2.44	0.043776	DL3140C	Pentatricopeptide repeat (PPR) superfamily protein
-3.0312	0.0091981	DL3195C	Transducin/WD40 repeat-like superfamily protein
-2.3189	7.13E-07	XBAT34	hypothetical protein
-3.1944	0.042609	DL3230W	cotton fiber protein
-2.0163	1.32E-06	HCD1	3-hydroxyacyl-CoA dehydratase 1
1.5073	0.00066941	CIPK4	CBL-interacting protein kinase 4
-3.9356	0.038337	CAM8	calmodulin 8
-3.1125	0.0012865	IQD19	IQ-domain 19
-0.86793	0.021073	NET1B	kinase interacting (KIP1-like) family protein
-3.1658	0.031975	GPAT3	glycerol-3-phosphate acyltransferase 3
-1.0426	0.046603	DL3768W	Senescence/dehydration-associated protein-like protein
0.83362	0.028633	UGT84A3	UDP-Glycosyltransferase superfamily protein
-1.7428	0.037516	UGT84A4	UDP-Glycosyltransferase superfamily protein
1.0599	0.0015793	UMAMIT38	EamA-like transporter family
-1.6241	0.0099119	P-like proteir	Uncharacterized protein family (UPF0497)
-1.136	0.0093679	P-like proteir	Uncharacterized protein family (UPF0497)
-0.78742	0.038965	RALFL33	ralf-like 33
-4.0117	0.019462	DL3955C	ARM repeat superfamily protein
1.6268	5.69E-07	DL4040C	hypothetical protein

-4.1169	0.026211	DL4110W	proline-rich family protein
1.1121	0.00026199	DL4135W	Papain family cysteine protease
-1.4577	0.02573	AtCYS4	Cystatin/monellin superfamily protein
-1.6663	0.0074329	DL4400C	Integrase-type DNA-binding superfamily protein
-1.0969	0.0021235	HB-2	homeobox protein 2
0.91809	0.030557	DL4420C	hydroxyproline-rich glycoprotein family protein
-3.8192	0.00014452	DL4515C	Protein kinase superfamily protein
-0.87006	0.013316	RLM3	disease resistance protein (TIR-NBS class)
-2.6171	0.00034228	EXLB1	expansin-like B1
-1.5673	0.030744	EDA8	Putative membrane lipoprotein
-1.6017	0.02741	DL4655C	structural maintenance of chromosomes protein
-0.86239	0.018044	GATL6	galacturonosyltransferase 6
-3.3431	6.10E-06	DL4875C	SBP (S-ribonuclease binding protein) family protein
-1.0921	0.0072305	T2H3.13	Drought-responsive family protein
-0.94212	0.0035785	T6K21.210	S-adenosyl-L-methionine-dependent methyltransferases superfamily protein
-1.1894	0.030862	T9A21.190	Glycosyl hydrolase superfamily protein
-4.753	0.027659	MRH1	Leucine-rich repeat protein kinase family protein
-1.3767	5.80E-06	F28A21.80	Leucine-rich repeat (LRR) family protein
-1.1248	0.011803	F28A21.150	Rho termination factor
-1.8124	0.014151	RLP51	receptor like protein 51
-3.2258	0.00083369	F13C5.1	GDSL-like Lipase/Acylhydrolase superfamily protein
-2.0109	0.048239	CYP707A1	cytochrome P450%2C family 707%2C subfamily A%2C polypeptide 1
1.3681	0.012753	T5K18.150	chitin synthase%2C putative (DUF1218)
1.1392	0.01271	AT4G19645	TRAM%2C LAG1 and CLN8 (TLC) lipid-sensing domain containing protein
-1.0559	0.029316	GALS3	hypothetical protein
-1.1487	0.021947	FAH2	fatty acid hydroxylase 2
-4.1548	0.0038987	GHR1	Leucine-rich receptor-like protein kinase family protein
-3.0056	0.039657	ATK1	kinesin 1
-1.1961	0.0016594	RK3	receptor kinase 3
-0.9097	0.01729	YAB3	Plant-specific transcription factor YABBY family protein
-1.3065	0.00018749	CRK41	cysteine-rich RLK (RECEPTOR-like protein kinase) 41
-1.1308	0.0070208	AT4G00955	wall-associated receptor kinase-like protein
-1.7823	0.0002454	MEE47	hypothetical protein (DUF688)
-0.9289	0.02059	BAM2	beta-amylase 2
1.4374	0.00012986	Tic20-IV	translocon at the inner envelope membrane of chloroplasts 20-IV
-2.3378	1.73E-11	CYP86A2	cytochrome P450%2C family 86%2C subfamily A%2C polypeptide 2
-2.4198	0.0056366	XTH9	xyloglucan endotransglucosylase/hydrolase 9
-1.6834	0.02864	tin receptor l	Concanavalin A-like lectin protein kinase family protein
-6.5879	0.0030665	F2N1.19	RING/U-box superfamily protein
-1.0131	0.011301	GRH1	GRR1-like protein 1
-3.0029	0.029227	ATPMEPCRB	Plant invertase/pectin methylesterase inhibitor superfamily
1.1073	0.005029	T15B16.24	GRAM domain family protein
-1.3475	1.54E-05	RBP-DR1	RNA-binding protein-defense related 1
-3.2886	0.044765	F4C21.2	Rho GTPase activating protein with PAK-box/P21-Rho-binding domain-containing protein
-3.0345	0.027659	T10M13.12	transcription coactivator
-2.0013	0.019596	GPAT8	glycerol-3-phosphate acyltransferase 8
-3.4923	1.89E-07	PRL	Minichromosome maintenance (MCM2/3/5) family protein
-4.5571	0.00041514	T5J8.17	phenazine biosynthesis PhzC/PhzF family protein
-1.5747	0.017352	F17L22.110	Subtilase family protein
-1.4774	0.0035095	F17L22.200	transmembrane protein
-0.87646	0.011275	ATML1	Homeobox-leucine zipper family protein / lipid-binding START domain-containing protein

-1.543	0.03034	T8O5.100	zinc finger MYND domain protein
1.4258	0.0006354	F1N20.1	hypothetical protein
-2.0854	1.45E-06	SRF8	STRUBBELIG-receptor family 8
-0.96413	0.04909	T10I14.20	serine/arginine repetitive matrix-like protein
-1.0254	0.0089609	T10I14.120	Ubiquitin-specific protease family C19-related protein
-1.3502	0.015625	T12H17.120	Leucine-rich repeat protein kinase family protein
-1.5005	0.031975	TRM11	hypothetical protein
-0.87323	0.043461	CRK5	cysteine-rich RLK (RECEPTOR-like protein kinase) 5
-1.4864	2.29E-07	CRK10	cysteine-rich RLK (RECEPTOR-like protein kinase) 10
-5.85	0.008986	CRK12	cysteine-rich RLK (RECEPTOR-like protein kinase) 12
-1.8068	0.00015721	CRK14	cysteine-rich RECEPTOR-like kinase
-1.4235	0.0085609	PIP1%3B5	plasma membrane intrinsic protein 1%3B5
-1.2758	0.044033	F16G20.2	fringe-like protein (DUF604)
-1.3634	3.00E-06	SGT1A	phosphatase-like protein
-1.2976	5.59E-06	F9D16.140	Polyketide cyclase/dehydrase and lipid transport superfamily protein
-1.4952	0.0025474	TBL24	TRICHOME BIREFRINGENCE-LIKE 24
-2.9111	0.00017617	3xHMG-box2	HMG (high mobility group) box protein
-2.6855	5.88E-13	T32A16.3	Pectin lyase-like superfamily protein
-2.9093	0.0073155	CSLG1	cellulose synthase like G1
-3.6678	0.016012	AT4G24275	hypothetical protein
-2.2575	0.00042946	CER2	HXXXD-type acyl-transferase family protein
-1.528	1.13E-05	AGL24	AGAMOUS-like 24
-0.90251	0.029303	M7J2.10	hydroxyproline-rich glycoprotein family protein
-1.1004	0.0003492	ABCB2	P-glycoprotein 2
-1.0067	0.0069968	MEK1	MAP kinase/ ERK kinase 1
-0.78152	0.033707	M3E9.170	RING/U-box superfamily protein
-1.0092	0.0035095	SHV3	PLC-like phosphodiesterase family protein
-0.97334	0.030492	MAP65-2	microtubule-associated protein 65-2
-6.0842	0.032596	F10M23.300	hypothetical protein
-0.8534	0.033347	CIP7	COP1-interacting protein 7
-1.044	0.019919	ENODL2	early nodulin-like protein 2
-1.4674	0.00015064	T29A15.210	Major facilitator superfamily protein
2.0766	0.047589	AT4G28005	NADH dehydrogenase ubiquinone 1 alpha subcomplex subunit
-1.3893	0.0015934	TET7	tetraspanin7
-1.6053	0.014239	F26K10.30	hypothetical protein (DUF789)
-3.0872	5.90E-05	ULT1	Developmental regulator%2C ULTRAPETALA
-0.75166	0.031232	F20O9.80	Protein phosphatase 2C family protein
-6.6187	0.0026641	F19B15.50	glycine-rich protein
-1.5195	0.019558	PAP2	phytochrome-associated protein 2
1.5553	0.027408	F19B15.140	cotton fiber protein
-2.0011	1.22E-07	F6G3.50	PA-domain containing subtilase family protein
-1.1958	0.003139	F6G3.90	Core-2/I-branching beta-1%2C6-N-acetylglucosaminyltransferase family protein
-1.6553	0.00030011	F17I23.250	sequence-specific DNA binding transcription factor
-1.2834	0.00054334	GAE1	UDP-D-glucuronate 4-epimerase 1
-1.4266	0.013963	CNGC9	cyclic nucleotide gated channel 9
-0.94966	0.028683	F17I23.10	Low temperature and salt responsive protein family
-5.4586	0.000415	SDG4	SET domain group 4
-0.89871	0.024266	NKS1	ubiquitin-associated protein (DUF1068)
-2.1659	4.60E-07	F6I18.90	Calmodulin-binding protein
1.0512	0.048161	AtNRX2	protein kinase C-like zinc finger protein
-3.1285	0.00022683	ENODL15	early nodulin-like protein 15
-4.4029	1.93E-05	F11C18.90	ARM repeat superfamily protein
-0.83279	0.010657	CESA1	cellulose synthase 1
-1.4103	0.0017763	VTE1	tocopherol cyclase%2C chloroplast / vitamin E deficient 1 (VTE1) / sucrose export defective 1 (SXD1)

-1.0038	0.030435	ATH1	homeobox protein ATH1
1.1577	0.0080263	F4I10.5	Thioredoxin superfamily protein
-0.78906	0.037148	F4I10.40	S-adenosyl-L-methionine-dependent methyltransferases superfamily protein
0.8778	0.047447	RPD1	Ubiquitin carboxyl-terminal hydrolase family protein
-2.0003	0.028963	T16L1.50	Wound-responsive family protein
-7.2245	4.24E-05	CER4	Jojoba acyl CoA reductase-related male sterility protein
-1.2268	0.047648	F28A23.90	Calcium-dependent lipid-binding (CaLB domain) family protein
-1.7139	0.020553	FUC95A	1%2C2-alpha-L-fucosidase
-0.77965	0.027659	T4L20.60	O-Glycosyl hydrolases family 17 protein
#NAME?	0.011592	COW1	Sec14p-like phosphatidylinositol transfer family protein
-0.79282	0.031975	T4L20.210	prostatic spermine-binding-like protein
-2.2666	0.0042471	SQS2	squalene synthase 2
-3.4429	0.0024644	F23E12.120	hypothetical protein
-2.1561	0.045595	XCP1	xylem cysteine peptidase 1
1.9045	0.0021797	F23E12.60	SEC7-like guanine nucleotide exchange family protein
1.2255	0.0017763	F4B14.4	SEC14 cytosolic factor family protein / phosphoglyceride transfer family protein
-1.0323	0.031289	MCA1	PLAC8 family protein
-1.1022	0.016011	FAH1	ferulic acid 5-hydroxylase 1
-1.932	0.0040651	GATA7	GATA transcription factor 7
-1.6078	0.0061848	F23E13.160	ATP binding protein
-2.6039	0.022169	BGAL3	beta-galactosidase 3
-1.3586	0.0094977	AP22.27	PQ-loop repeat family protein / transmembrane family protein
3.6227	1.10E-07	CP1	cysteine proteinase1
1.3145	0.047648	AP22.73	membrane protein
-0.91142	0.014352	AP22.58	ternary complex factor MIP1 leucine-zipper protein (Protein of unknown function%2C DUF547)
-1.238	0.036722	AP22.72	Zinc-finger domain of monoamine-oxidase A repressor R1
-3.914	1.84E-05	AP22.53	HTH-type transcriptional regulator
-1.1985	0.019131	MYB73	myb domain protein 73
-3.6391	0.035604	AGP18	arabinogalactan protein 18
-0.80485	0.022884	F19F18.40	Acetamidase/Formamidase family protein
-2.5352	0.0004649	ANT	Integrase-type DNA-binding superfamily protein
-0.89456	0.016727	SQE3	squalene epoxidase 3
-3.3045	0.0034011	ACS8	1-amino-cyclopropane-1-carboxylate synthase 8
-4.3679	5.37E-13	XTH7	xyloglucan endotransglucosylase/hydrolase 7
-2.186	0.001018	EDA40	Zinc finger (C3HC4-type RING finger) family protein
3.3412	0.0011674	F22I13.110	Plant regulator RWP-RK family protein
1.1866	0.0020114	F22I13.150	MATE efflux family protein
-3.7807	0.00065725	T9A14.6	Pathogenesis-related thaumatin superfamily protein
-3.0884	0.036079	PRP4	proline-rich protein 4
2.0111	0.0030886	T9A14.60	pre-mRNA-processing-splicing factor-like protein
-1.6552	0.00092149	BBX19	B-box type zinc finger family protein
-0.80241	0.027383	F19H22.150	Kinesin motor family protein
1.4978	0.00063935	CCR1	cold%2C circadian rhythm%2C and RNA binding 1
-1.9223	0.031975	CAD9	cinnamyl alcohol dehydrogenase 9
-0.76858	0.034034	CESA2	cellulose synthase A2
-3.4368	0.019173	CYP96A12	cytochrome P450%2C family 96%2C subfamily A%2C polypeptide 12
1.0366	0.007807	T19P19.170	Integrase-type DNA-binding superfamily protein
-1.5019	0.00022154	T5J17.70	adenine deaminase
1.1225	0.00088958	SYTC	Calcium-dependent lipid-binding (CaLB domain) family protein
-3.5376	2.90E-06	KCS19	3-ketoacyl-CoA synthase 19
1.065	0.046069	ABO6	DEA(D/H)-box RNA helicase family protein

-0.70126	0.045266	CEV1	Cellulose synthase family protein
1.8653	0.044387	K18I23.12	FAD/NAD(P)-binding oxidoreductase family protein
1.3272	0.027659	K18I23.24	RNA-binding protein
-1.8985	0.0022555	FLA17	FASCICLIN-like arabinogalactan protein 17 precursor
-0.79669	0.020102	MPH15.5	trichome birefringence-like protein (DUF828)
-4.1197	0.0053225	MOJ9.10	nucleolar-like protein
-1.1427	0.013076	LNK4	hypothetical protein
-2.1469	0.01707	ST2B	sulfotransferase 2B
-4.7788	0.0022408	MOJ9.20	Eukaryotic aspartyl protease family protein
1.5688	0.00041981	MAC5C	CCCH-type zinc fingerfamily protein with RNA-binding domain-containing protein
-1.3228	0.043176	PRA1.B6	prenylated RAB acceptor 1.B6
-1.1658	0.049275	ERL2	ERECTA-like 2
-0.75416	0.035868	BAG3	BCL-2-associated athanogene 3
-1.3084	3.42E-05	IQD24	IQ-domain 24
-2.4255	0.0075952	SMC6A	structural maintenance of chromosomes 6A
-1.216	0.00074007	MYB29	myb domain protein 29
-1.9167	0.0004694	F13G24.70	HXXXD-type acyl-transferase family protein
-3.3432	0.030924	E13L3	glucan endo-1%2C3-beta-glucosidase-like protein 3
-5.0019	0.0020567	RPA70B	RPA70-kDa subunit B
-2.7172	0.00060927	SOB5	suppressor of phytochrome b 5
-1.824	2.36E-05	scpl35	serine carboxypeptidase-like 35
-0.88337	0.0086917	TCP11	TCP family transcription factor
0.73072	0.041277	PDE340	P-loop containing nucleoside triphosphate hydrolases superfamily protein
-1.9877	0.026098	T5E8.90	Inositol monophosphatase family protein
1.1267	0.035879	T5E8.100	Thiamin diphosphate-binding fold (THDP-binding) superfamily protein
-1.5976	4.30E-05	F17I14.50	Plant invertase/pectin methylesterase inhibitor superfamily
1.2263	0.0031171	T10O8.30	Thiamine pyrophosphate dependent pyruvate decarboxylase family protein
-1.513	0.0004694	T31P16.140	UPSTREAM OF FLC protein (DUF966)
0.79552	0.014175	SULTR2%3B1	slufate transporter 2%3B1
2.6252	0.0082499	F18D22.2	nitric oxide synthase-interacting protein
-2.6195	0.0015621	F12B17.260	Histone superfamily protein
-2.5829	0.015999	F12B17.250	Histone superfamily protein
2.42	2.43E-05	RBK1	ROP binding protein kinases 1
-0.95259	0.0047283	GTE2	global transcription factor group E2
-3.284	0.007186	APT5	adenine phosphoribosyltransferase 5
-1.4465	0.042413	HY5	Basic-leucine zipper (bZIP) transcription factor family
-2.1784	1.62E-05	F15N18.10	transmembrane protein%2C putative (Protein of unknown function%2C DUF642)
-1.0471	0.00081623	AGP15	arabinogalactan protein 15
-2.1168	0.0039343	NDL2	N-MYC downregulated-like 2
-1.6779	0.025311	MXC9.7	elongation factor 1-beta 1
-1.4365	0.022349	T24H18.70	DNA double-strand break repair RAD50 ATPase
-3.4516	0.012089	T24H18.100	Transducin/WD40 repeat-like superfamily protein
-3.5433	0.00041514	T24H18.110	Leucine-rich repeat (LRR) family protein
-4.9168	0.00030075	ABAP1	ARMADILLO BTB protein 1
-1.3041	0.0041805	T19L5.60	Gap junction beta-4 protein
-3.967	0.00011041	T19L5.100	Pollen Ole e 1 allergen and extensin family protein
1.2123	3.79E-05	NAC083	NAC domain containing protein 83
-1.0446	0.038732	T22N19.10	Auxin-responsive GH3 family protein
-1.4885	5.70E-05	T22N19.50	Major facilitator superfamily protein
-2.7637	1.81E-06	IQD11	IQ-domain 11
-1.1872	0.019982	T6I14.50	peptidase M1 family protein
-0.70749	0.042678	SMT1	sterol methyltransferase 1
-2.6544	0.04127	APD7	Protein phosphatase 2C family protein

-1.0115	0.040019	AtLAZY1	regulator of nonsense transcript protein
3.4748	0.00025274	MPL1	Myzus persicae-induced lipase 1
1.1103	0.047192	T15N1.70	polyribonucleotide nucleotidyltransferase
-1.5604	0.044536	T15N1.100	DEAD box RNA helicase family protein
-1.1583	0.0049625	F2G14.170	Core-2/I-branching beta-1%2C6-N-acetylglucosaminyltransferase family protein
-2.0635	0.033441	GASA4	GAST1 protein homolog 4
-2.1838	1.16E-07	ENODL17	early nodulin-like protein 17
-2.8015	0.023765	LNG1	longifolia1
0.90938	0.031812	F14F8.80	DNA/RNA polymerases superfamily protein
-5.3329	1.31E-07	F14F8.160	Pollen Ole e 1 allergen and extensin family protein
-2.1599	1.51E-06	bZIP3	basic leucine-zipper 3
-1.0563	0.047648	COL1	CONSTANS-like 1
-1.7581	8.35E-08	F1N13.10	glycosyl hydrolase family 81 protein
-0.98295	0.027659	PAM1	plant adhesion molecule 1
-1.4032	0.0013349	KIN2	stress-responsive protein (KIN2) / stress-induced protein (KIN2) / cold-responsive protein (COR6.6) / cold-regulated protein (COR6.6)
-1.6599	0.00030117	NIK1	NSP-interacting kinase 1
-1.0969	0.022061	F1N13.170	mental retardation GTPase activating protein
-2.6855	0.00030243	T21H19.170	transmembrane protein
-5.2642	0.018129	MQK4.7	O-acyltransferase (WSD1-like) family protein
1.0034	0.011616	MQK4.11	autophagy-like protein%2C putative (Protein of unknown function%2C DUF538)
-0.81636	0.032175	CAC1	chloroplastic acetylcoenzyme A carboxylase 1
-2.3387	0.0023015	ORC3	origin recognition complex subunit 3
-0.96161	0.013502	F15A17.170	Concanavalin A-like lectin protein kinase family protein
-1.8949	0.02889	RVE1	Homeodomain-like superfamily protein
-1.642	0.0086043	PXC2	Leucine-rich receptor-like protein kinase family protein
-0.91636	0.0076619	iqd2	IQ-domain 2
-1.2192	0.0082597	TBL35	TRICHOME BIREFRINGENCE-LIKE 35
-5.6774	0.0010231	ATCSLA09	Nucleotide-diphospho-sugar transferases superfamily protein
1.0302	0.026165	HSFA3	heat shock transcription factor A3
-1.2249	0.0081102	NCS1	permease%2C cytosine/purines%2C uracil%2C thiamine%2C allantoin family protein
-3.1308	1.84E-05	F15A17.150	transmembrane protein
-1.0836	0.022422	RABC2A	RAB GTPase homolog C2A
-4.1296	0.00060927	F12E4.130	hypothetical protein (DUF295)
-3.0427	0.045266	F12E4.80	Legume lectin family protein
-2.9767	0.02517	AT5G01075	Glycosyl hydrolase family 35 protein
-2.8633	0.0070088	LOG6	lysine decarboxylase family protein
1.0473	0.0019725	F7J8.190	HXXXD-type acyl-transferase family protein
-2.9447	0.0031444	ACI1	ALC-interacting protein 1
-1.2337	0.049146	JKD	C2H2-like zinc finger protein
-5.2727	0.0026315	F15A17.160	hypothetical protein
-1.516	0.0050832	F20L16.180	carboxyl-terminal peptidase (DUF239)
-1.6762	0.0011468	BMY3	beta-amylase 3
-1.5521	0.010471	T16G12.130	Heavy metal transport/detoxification superfamily protein
0.74514	0.030365	AILP1	aluminum induced protein with YGL and LRDR motifs
-2.4075	0.0014381	T24G5.140	Glycoprotein membrane precursor GPI-anchored
1.2509	0.00026534	T29J13.160	Peptidase M28 family protein
-1.1236	0.0024644	NUDX19	nudix hydrolase homolog 19
-1.97	3.64E-09	HHP1	heptahelical transmembrane protein1
-1.0705	0.030744	BRXL4	BREVIS RADIX-like 4
-1.3444	6.78E-06	T10F18.130	Plant L-ascorbate oxidase
-0.78624	0.022303	J2	DNAJ homologue 2
1.1434	0.013316	NIT4	nitrilase 4

-3.2027	4.29E-05	MWD9.26	alpha/beta-Hydrolases superfamily protein
-3.9933	0.00015177	MQJ16.12	Stress responsive A/B Barrel Domain-containing protein
-1.6675	0.030924	HTB2	histone B2
-4.0226	0.005226	F8H	glucuronoxylan glucuronosyltransferase%2C putative
-1.194	0.02501	TUB8	tubulin beta 8
-0.87302	0.0060577	MOP9.2	alpha/beta-Hydrolases superfamily protein
1.7022	0.002557	PRR5	two-component response regulator-like protein
-1.6446	0.007202	DMR6	2-oxoglutarate (2OG) and Fe(II)-dependent oxygenase superfamily protein
-3.8575	0.00073896	K18P6.11	Heavy metal transport/detoxification superfamily protein
-1.0235	0.044765	LSU2	response to low sulfur 2
-1.0347	0.0012396	CYP71B12	cytochrome P450%2C family 71%2C subfamily B%2C polypeptide 12
-0.94571	0.012391	CYP71B13	cytochrome P450%2C family 71%2C subfamily B%2C polypeptide 13
-2.7664	3.55E-05	ESE3	Integrase-type DNA-binding superfamily protein
-3.6405	0.047889	F21J6.109	SPFH/Band 7/PHB domain-containing membrane-associated protein family
-1.0558	0.03023	F18G18.180	Protein kinase superfamily protein
-1.7811	4.64E-05	DGR2	transmembrane protein%2C putative (Protein of unknown function%2C DUF642)
-3.495	0.0023359	T14C9.30	Ran BP2/NZF zinc finger-like superfamily protein
-1.9097	0.00029603	T14C9.130	DNA ligase (DUF630 and DUF632)
-2.0951	3.28E-15	RD22	BURP domain-containing protein
-5.3217	0.021589	TGG2	glucoside glucohydrolase 2
-2.7905	0.043691	TGG1	thioglucoside glucohydrolase 1
0.91086	0.016396	MSS1	Major facilitator superfamily protein
-0.77099	0.036427	PYR6	P-loop containing nucleoside triphosphate hydrolases superfamily protein
-2.7129	0.0042174	AT5G26690	Heavy metal transport/detoxification superfamily protein
-3.1402	0.0084674	AT5G26670	Pectinacetylesterase family protein
-1.4838	0.004424	F2P16.24	Uncharacterized protein
1.4363	0.049713	T21B4.140	Frigida-like protein
1.152	0.034384	DES1	L-cysteine desulfhydrase 1
-0.81181	0.032282	F15F15.120	hypothetical protein
-1.671	0.029075	NEK3	NIMA-related kinase 3
1.0203	0.0043826	XGD1	xylogalacturonan deficient 1
-1.7993	0.00054331	GUS3	glucuronidase 3
1.06	0.0026483	T26D22.12	S-locus lectin protein kinase family protein
-2.3516	0.00030102	iqd33	IQ-domain 33
-0.88869	0.0070208	MAB16.11	Tyrosine transaminase family protein
-4.3011	0.0069118	K15O15.2	glycosyltransferase family exostosin protein
-0.80392	0.048681	ARF8	auxin response factor 8
-3.7585	0.020308	SUS5	sucrose synthase 5
0.93986	0.042219	GSR 1	hypothetical protein
-2.7481	0.000724	PDLP7	plasmodesmata-located protein 7
-1.8416	8.82E-06	K22F20.5	Protein kinase superfamily protein
-1.0112	0.0053273	MUL8.23	hypothetical protein (DUF1997)
-5.5256	0.00016001	VIM3	Zinc finger (C3HC4-type RING finger) family protein
-1.4576	2.14E-05	CDF2	cycling DOF factor 2
1.3183	0.00066385	UMAMIT40	nodulin MtN21 /EamA-like transporter family protein
-2.0468	0.037256	DRB5	dsRNA-binding protein 5
-2.2521	0.003786	MEE6.21	Myosin heavy chain-related protein
-1.8883	0.0026301	MEE6.25	leucine-rich repeat transmembrane protein kinase family protein
0.80112	0.022091	BEL1	POX (plant homeobox) family protein
-2.6213	1.33E-05	MYC6.16	transferring glycosyl group transferase (DUF604)
3.0358	0.012089	MBK23.11	LURP-one-like protein (DUF567)

-1.5522	4.72E-05	MUF8.2	Disease resistance protein (TIR-NBS-LRR class) family
-1.9458	0.00044739	MUF8.3	hypothetical protein
-2.969	0.0043826	POLA3	DNA primase POLA3
-3.0023	6.49E-06	K16L22.19	alpha/beta-Hydrolases superfamily protein
-2.1719	0.0059837	MDH9.23	hypothetical protein
-1.2931	0.0092063	MJB21.5	MIZU-KUSSEI-like protein (Protein of unknown function%2C DUF617)
-2.1491	0.0074956	MJB21.9	Glycosyl hydrolase family 17 protein
-2.5495	0.0027485	MBD2.2	HXXXD-type acyl-transferase family protein
0.85852	0.037951	MWF20.15	2-oxoglutarate (2OG) and Fe(II)-dependent oxygenase superfamily protein
-1.2498	0.021721	TZP	zinc knuckle (CCHC-type) family protein
-0.92707	0.013174	TRM21	methyl-coenzyme M reductase II subunit gamma%2C putative (DUF3741)
-2.2153	0.019433	SUVR2	SET-domain containing protein lysine methyltransferase family protein
-1.4209	0.0057248	TUB4	tubulin beta chain 4
-1.5899	0.0011468	K9L2.20	FAD-binding Berberine family protein
1.0071	0.048569	MFC16.16	FBD%2C F-box%2C Skp2-like and Leucine Rich Repeat domains containing protein
-3.3498	1.33E-08	MCM6	minichromosome maintenance (MCM2/3/5) family protein
-1.7396	0.047838	K23L20.1	DNA glycosylase superfamily protein
-0.94167	0.011728	BRG1	SBP (S-ribonuclease binding protein) family protein
-2.7557	1.79E-05	K9E15.6	Pectinacetylesterase family protein
-3.5401	0.019173	CYP707A3	cytochrome P450%2C family 707%2C subfamily A%2C polypeptide 3
-1.6922	0.0095641	MFC19.16	P-loop containing nucleoside triphosphate hydrolases superfamily protein
-1.9299	0.023107	MFC19.23	Pleckstrin homology (PH) domain-containing protein / lipid-binding START domain-containing protein
#NAME?	4.22E-05	MRA19.6	GDSL-like Lipase/Acylhydrolase superfamily protein
1.9341	0.00018749	K15I22.4	Leucine-rich repeat protein kinase family protein
-1.5718	0.031975	K15I22.15	GDSL-like Lipase/Acylhydrolase superfamily protein
1.431	0.0089418	MCL19.3	crooked neck protein%2C putative / cell cycle protein
-2	8.32E-07	bHLH071	beta HLH protein 71
-3.2456	0.0091203	MZA15.15	glycine-rich protein
#NAME?	0.033707	NIG1	calcium-binding transcription factor NIG1
-2.7056	5.69E-07	MQL5.24	electron transporter%2C putative (Protein of unknown function%2C DUF547)
-2.4624	0.0018111	MNJ7.2	hypothetical protein
-3.1567	0.00037693	PME5	Pectin lyase-like superfamily protein
-1.3895	0.0013215	MNJ7.14	Cystatin/monellin superfamily protein
-1.1928	0.0053811	MNJ7.20	RING/U-box superfamily protein
-5.9635	0.019678	K23F3.3	portal protein
-1.4921	0.0094977	sks3	SKU5 similar 3
-1.3065	0.047192	AT5G48460	Actin binding Calponin homology (CH) domain-containing protein
-1.5437	0.007202	CIB2	basic helix-loop-helix (bHLH) DNA-binding superfamily protein
-2.5689	0.0012167	ATFKBP65	FKBP-type peptidyl-prolyl cis-trans isomerase family
-1.2848	0.024266	K24G6.13	Phototropic-responsive NPH3 family protein
-1.7806	0.029603	K19E20.1	Pectin lyase-like superfamily protein
-0.91734	0.0068584	HCT	hydroxycinnamoyl-CoA shikimate/quinate hydroxycinnamoyl transferase
-4.1838	0.023745	K21P3.4	hypothetical protein
-2.0005	0.0012338	AT5G49215	Pectin lyase-like superfamily protein
-1.0603	0.0037161	ACLB-2	hypothetical protein
-1.5589	0.012343	AT5G49680	SABRE-like protein

-0.73725	0.032013	GH9A1	glycosyl hydrolase 9A1
-3.5641	0.048239	K21G20.1	Polyketide cyclase/dehydrase and lipid transport superfamily protein
1.0321	0.022281	FRO8	ferric reduction oxidase 8
-3.4526	0.0002454	AT5G50335	hypothetical protein
-2.4981	0.00027502	AT5G50915	basic helix-loop-helix (bHLH) DNA-binding superfamily protein
-2.1685	1.68E-05	ATTPPA	Haloacid dehalogenase-like hydrolase (HAD) superfamily protein
-3.2008	0.041129	K17N15.11	Leucine-rich repeat protein kinase family protein
-2.5088	0.028679	NEET	2 iron%2C 2 sulfur cluster binding protein
-2.1857	0.0030264	MJM18.4	Peroxidase superfamily protein
-1.1124	0.0046197	BAG1	BCL-2-associated athanogene 1
0.93769	0.013316	PP2-A14	phloem protein 2-A14
-1.8841	1.13E-07	F17P19.19	Myosin heavy chain-related protein
-1.3209	2.53E-05	F6N7.1	keratin-associated protein%2C putative (DUF819)
-2.0718	0.046149	MXC20.3	LIM domain protein
-1.4957	0.000415	PMEPCRF	pectin methylesterase PCR fragment F
1.2588	2.25E-05	MYN8.3	CCT motif family protein
-1.3795	0.0060375	ENODL1	early nodulin-like protein 1
-0.98943	0.0019904	OMT1	O-methyltransferase 1
-2.3516	2.77E-05	K18G13.8	Transducin/WD40 repeat-like superfamily protein
-1.1087	0.0029246	THE1	protein kinase family protein
-2.1429	2.05E-06	ANK	ankyrin
-1.9496	0.0095585	MBG8.11	hexon
-1.8744	0.041982	MBG8.24	hypothetical protein
-2.2942	2.04E-06	MTE17.11	Actin binding Calponin homology (CH) domain-containing protein
-2.3634	3.35E-10	MTE17.16	Bifunctional inhibitor/lipid-transfer protein/seed storage 2S albumin superfamily protein
-1.0764	0.037076	MTE17.17	Bifunctional inhibitor/lipid-transfer protein/seed storage 2S albumin superfamily protein
-1.9872	0.017508	SVL1	SHV3-like 1
-5.9673	0.047262	MTE17.24	kinesin-like protein
-4.2948	0.017005	FLA1	FASCICLIN-like arabinogalactan 1
-3.0208	0.016144	WYR	inner centromere protein%2C ARK-binding region protein
-0.88929	0.0074329	HSP81-3	heat shock protein 81-3
-1.3357	0.00022672	HSP81-2	heat shock protein 81-2
2.3319	0.022169	K24C1.1	C2H2 type zinc finger transcription factor family
-1.0609	0.027084	MKN22.4	tRNA-splicing ligase (DUF239)
1.7206	0.010547	MIK19.15	FBD / Leucine Rich Repeat domains containing protein
-5.1945	3.65E-14	XTH25	xyloglucan endotransglucosylase/hydrolase 25
-3.6399	4.90E-07	TCH4	Xyloglucan endotransglucosylase/hydrolase family protein
-1.4878	0.0013084	MRI1.6	BNR/Asp-box repeat family protein
-1.8198	0.00014182	P1R1	transcription factor
-4.5373	0.0028999	MTI20.23	DNA glycosylase superfamily protein
-1.3282	0.001	K21L19.1	Disease resistance protein (TIR-NBS-LRR class) family
-0.97071	0.047595	MCK7.17	Leucine-rich repeat protein kinase family protein
-3.1145	1.21E-09	MCK7.26	Peroxidase superfamily protein
-0.8908	0.014595	PLC1	phospholipase C1
-2.1992	0.00037989	K19M22.16	hepatocyte growth factor activator%2C putative (DUF3527)
-0.90205	0.012517	UXS3	UDP-glucuronic acid decarboxylase 3
0.79565	0.018021	MYB59	myb domain protein 59
-3.1502	0.03905	HTA6	histone H2A 6
-1.4133	0.0064448	MMN10.3	Histone superfamily protein
1.1235	0.024175	PRR3	pseudo-response regulator 3
-2.7936	0.014668	FLA12	FASCICLIN-like arabinogalactan-protein 12
0.86126	0.013476	PDX2	pyridoxine biosynthesis 2

-3.0197	3.65E-12	MAE1.5	Heavy metal transport/detoxification superfamily protein
-0.99779	0.026568	RABA1f	RAB GTPase homolog A1F
-1.1067	0.013956	RLK1	receptor-like protein kinase 1
-3.3728	0.0018111	MSL3.50	P-loop containing nucleoside triphosphate hydrolases superfamily protein
-1.7965	0.0024878	COBL5	COBRA-like protein 5 precursor
-3.1886	0.00023815	RPA70D	Replication factor-A protein 1-like protein
-1.5168	0.0030844	ACHT5	atypical CYS HIS rich thioredoxin 5
-0.97018	0.015314	IQD23	IQ-domain 23
-2.1957	8.09E-07	BAG2	BCL-2-associated athanogene 2
-1.4194	0.0032609	GT18	glycosyltransferase 18
-2.6178	0.0016346	EB1B	end binding protein 1B
-4.1272	7.52E-05	K19B1.16	microtubule-associated futsch-like protein
-1.6817	0.0050819	K19B1.19	ARM repeat superfamily protein
-1.806	4.20E-06	HA11	H[+]-ATPase 11
-1.3344	1.10E-05	TUB3	tubulin beta chain 3
-2.2756	0.011658	MDC12.15	Pectin lyase-like superfamily protein
0.84362	0.027659	ARI15	RING/U-box superfamily protein
-2.1114	0.0072305	TOP3A	topoisomerase 3alpha
-0.78437	0.025311	NPH3	Phototropic-responsive NPH3 family protein
-1.559	0.028576	FAS2	Transducin/WD40 repeat-like superfamily protein
-1.0904	0.00042591	CESA6	cellulose synthase 6
-1.06	0.027659	ASN2	asparagine synthetase 2
-4.9237	6.18E-05	AGP7	arabinogalactan protein 7
-1.2378	0.011592	MNA5.17	transmembrane protein
-2.3332	3.36E-06	bHLH093	beta HLH protein 93
-3.2863	3.39E-05	XTH6	xyloglucan endotransglucosylase/hydrolase 6
-1.1046	0.0062602	CGR3	transmembrane protein
-1.7801	1.85E-05	K14B20.9	ARM repeat superfamily protein
0.98707	0.0021797	AT5G66052	transmembrane protein
-2.1787	0.0050737	K1L20.9	ATP binding microtubule motor family protein
-1.5543	0.011592	DAR7	DA1-related protein 7
-1.6242	0.0038481	DAR6	DA1-related protein 6
-1.458	0.02893	DAR3	DA1-related protein 3
-4.0663	0.0009463	MUD21.4	membrane-associated kinase regulator-like protein
-4.6369	9.59E-18	sks17	SKU5 similar 17
-2.4351	0.0043278	RALFL34	ralf-like 34
-2.6721	1.81E-05	ICU2	DNA-directed DNA polymerase
-0.78765	0.027153	AT5G67385	Phototropic-responsive NPH3 family protein
-3.9374	0.044335	K9I9.2	O-Glycosyl hydrolases family 17 protein
1.6166	3.75E-07	ZIP5	zinc transporter 5 precursor
1.4933	2.24E-05	YUP8H12.7	Pectin lyase-like superfamily protein
-2.1699	0.012391	CVP2	DNAse I-like superfamily protein
-1.0328	0.002557	URH2	uridine-ribohydrolase 2
-1.7161	0.0020114	POM1	Chitinase family protein
#NAME?	0.0038987	ADS1	delta 9 desaturase 1
#NAME?	0.0051957	T2D23.6	Fatty acid desaturase family protein
-1.9375	0.024927	FRO1	ferric reduction oxidase 1
-2.0176	2.66E-05	bZIP52	basic leucine-zipper 52
-0.75043	0.044226	EXO70B2	exocyst subunit exo70 family protein B2
-6.7977	8.90E-07	PCNA1	proliferating cellular nuclear antigen 1
-2.4613	0.0011468	F22G5.25	HSP20-like chaperones superfamily protein
-0.99178	0.0027131	KCS3	3-ketoacyl-CoA synthase 3
-0.77168	0.020904	CRT3	calreticulin 3
-1.0587	0.01934	PPCK1	phosphoenolpyruvate carboxylase kinase 1
-3.0606	0.0018111	H3.1	Histone superfamily protein
-1.9972	5.14E-14	T31J12.3	plant/protein (Protein of unknown function%2C DUF538)
-0.87931	0.043779	F14J9.5	GDSL-like Lipase/Acylhydrolase superfamily protein

1.7947	0.00020343	G6PD4	glucose-6-phosphate dehydrogenase 4
-1.1691	0.011301	F14J9.10	Protein kinase superfamily protein
1.0135	0.015763	F14J9.14	NAD(P)-binding Rossmann-fold superfamily protein
-1.098	0.00098218	GLP5	germin-like protein 5
-1.9018	0.0094422	AT1G09575	calcium uniporter-like protein (DUF607)
-2.0788	9.44E-05	F21M12.13	Eukaryotic aspartyl protease family protein
-2.2827	1.50E-05	AT1G09932	Phosphoglycerate mutase family protein
-1.8971	0.0052275	T27I1.4	formin-like protein (DUF1005)
-2.1292	0.0069527	ERD9	Glutathione S-transferase family protein
-6.0901	0.034546	F20B24.8	Pectin lyase-like superfamily protein
-0.73136	0.036847	ACLA-1	ATP-citrate lyase A-1
-1.0319	0.022884	T16B5.12	alpha/beta-Hydrolases superfamily protein
2.7224	0.010384	T16B5.9	Plant invertase/pectin methylesterase inhibitor superfamily protein
-5.9808	0.011622	XTH8	xyloglucan endotransglucosylase/hydrolase 8
-0.93327	0.010279	F12F1.13	Phosphofructokinase family protein
-1.1323	0.042225	BAG5	BCL-2-associated athanogene 5
-4.3525	5.88E-13	F12F1.4	Vacuolar calcium-binding protein-like protein
-2.0459	0.036835	ELP	extensin-like protein
-0.9761	0.030744	F5O11.6	cyclin-dependent kinase-like protein
-1.5577	0.030796	PHR1	photolyase 1
-3.2053	0.0096121	F5O11.10	hypothetical protein
-2.4454	0.030435	NIMIN1	NIM1-interacting 1
-1.465	0.0013884	F5O11.25	Nucleotide-sugar transporter family protein
-1.2954	0.002082	RBL6	RHOMBOID-like protein 6
-2.5677	0.00042327	AT1G12845	transmembrane protein
-1.1915	0.022959	CYP71B2	cytochrome P450%2C family 71%2C subfamily B%2C polypeptide 2
-1.9803	0.0018947	ORP1D	OSBP(oxysterol binding protein)-related protein 1D
1.0385	0.00098711	RTFL17	ROTUNDIFOLIA like 17
-3.1124	2.42E-08	GATL3	galacturonosyltransferase-like 3
-6.2992	0.0043169	F21F23.11	hypothetical protein
-0.94289	0.040779	PGL1	6-phosphogluconolactonase 1
-1.4097	0.029954	BXL2	beta-xylosidase 2
-1.0137	0.00089912	F7A19.29	RING/U-box superfamily protein
-1.3221	0.021397	AT1G14205	Ribosomal L18p/L5e family protein
-2.045	1.89E-07	MIOX1	myo-inositol oxygenase 1
-1.1377	0.020553	PAP3	purple acid phosphatase 3
-1.0058	0.015149	MAP70-4	microtubule-associated proteins 70-4
-1.6061	0.007426	AT1G14970	O-fucosyltransferase family protein
-0.94402	0.020499	scpl50	serine carboxypeptidase-like 50
0.86283	0.040891	ATHSRP54A	signal recognition particle 54 kDa subunit
-4.3478	0.032709	CYCA2%3B3	CYCLIN A2%3B3
-1.5968	0.032127	F7H2.23	hypothetical protein
1.4701	9.34E-05	CYP79F2	cytochrome P450%2C family 79%2C subfamily F%2C polypeptide 2
-1.189	0.010998	F3O9.32	interactor of constitutive active ROPs protein
-0.91722	0.029227	RABA1b	RAB GTPase homolog A1B
-1.6783	0.011144	ICR1	interactor of constitutive active rops 1
-1.4193	0.00035464	F28G4.9	alpha/beta-Hydrolases superfamily protein
0.9503	0.02933	TRFL3	TRF-like 3
-1.4112	0.00049651	F11A6.25	Late embryogenesis abundant (LEA) hydroxyproline-rich glycoprotein family
-4.3324	1.55E-05	PRA1.F1	prenylated RAB acceptor 1.F1
2.0842	0.049368	PEPC1	Pyridoxal phosphate phosphatase-related protein
-1.6686	0.046304	F2H15.6	hypothetical protein (DUF789)
-0.72644	0.038545	ABCG11	white-brown complex-like protein
#NAME?	5.66E-06	ATLP-1	Pathogenesis-related thaumatin superfamily protein

-2.5675	0.022484	BEE1	BR enhanced expression 1
-1.4265	0.0011513	TRM3	LONGIFOLIA protein
-1.0387	0.01271	PDCB3	plasmodesmata callose-binding protein 3
-0.95944	0.031082	IBS1	Protein kinase superfamily protein
-1.6884	0.00011041	IQD30	IQ-domain 30
-1.031	0.034109	F18O14.5	histone deacetylase complex subunit
-0.84486	0.013502	BES1	Brassinosteroid signaling positive regulator (BZR1) family protein
2.9977	0.014771	F14P1.15	Transducin/WD40 repeat-like superfamily protein
-1.3064	0.021548	HVA22H	HVA22-like protein H (ATHVA22H)
-2.5116	2.52E-05	T20H2.24	ER lumen protein retaining receptor family protein
1.4561	4.90E-05	T20H2.19	Pathogenesis-related thaumatin superfamily protein
1.9031	2.21E-06	T20H2.15	hypothetical protein
-2.5947	8.28E-05	EXPA11	expansin 11
-2.4569	0.037882	XCP2	xylem cysteine peptidase 2
-0.98596	0.0071638	F9H16.6	Phosphofructokinase family protein
-2.0526	0.0032778	T22I11.12	Serine/Threonine-kinase%2C putative (Protein of unknown function%2C DUF547)
-2.5155	0.00021767	T22I11.10	Nucleotide-sugar transporter family protein
-0.76635	0.041423	WAK1	hypothetical protein
1.2929	0.0042174	F8K7.10	DPP6 N-terminal domain-like protein
-4.3497	3.19E-11	DREB26	Integrase-type DNA-binding superfamily protein
0.81133	0.023789	TGA3	transcription factor TGA3
1.179	0.00022455	F2E2.23	senescence-associated family protein (DUF581)
-3.3644	0.004781	F16L1.4	nucleolar GTP-binding protein
-1.6057	8.83E-08	F12K8.24	Class-II DAHP synthetase family protein
1.5091	0.0052078	GI	gigantea protein (GI)
-0.89186	0.0084674	F19G10.22	transmembrane protein
-0.93628	0.011963	F19G10.13	RNA-binding (RRM/RBD/RNP motifs) family protein
-1.058	0.010943	F19G10.3	ARM repeat superfamily protein
-0.68197	0.048405	PIN7	Auxin efflux carrier family protein
0.91407	0.013058	T26J12.10	Polyketide cyclase/dehydrase and lipid transport superfamily protein
-1.8474	0.021317	CSLA03	cellulose synthase-like A3
-2.0284	0.025311	F5O8.30	Nucleic acid-binding%2C OB-fold-like protein
-2.1516	0.015924	T23E23.5	NHL domain-containing protein
-0.96423	0.029327	FH4	formin homologue 4
-2.3777	0.00011295	LGT9	Nucleotide-diphospho-sugar transferases superfamily protein
0.76643	0.044057	ZFP7	zinc finger protein 7
-4.19	0.037516	HTR12	Histone superfamily protein
0.84334	0.031232	F2J7.18	aluminum activated malate transporter family protein
0.94696	0.0069851	F2J7.20	Uncharacterized protein family (UPF0016)
-1.9188	2.92E-07	SOFL1	SOB five-like 1
-1.9103	0.01191	BGLU40	beta glucosidase 40
-1.1397	0.033707	GALT1	galactosyltransferase1
1.3261	0.034058	ABCB11	P-glycoprotein 11
-2.7133	0.028633	AT1G01225	NC domain-containing protein-like protein
-1.6295	0.01116	IQD18	IQ-domain 18
-2.0468	0.0003772	KCS1	3-ketoacyl-CoA synthase 1
-1.7771	0.00045884	TUA4	tubulin alpha-4 chain
#NAME?	0.0003228	F13M7.21	glycine-rich protein
-1.2983	6.78E-06	F13M7.24	Tetratricopeptide repeat (TPR)-like superfamily protein
1.7879	0.0005906	T14P4.24	hypothetical protein
-3.2613	5.64E-07	CTF18	P-loop containing nucleoside triphosphate hydrolases superfamily protein
-0.98281	0.010201	F20D22.3	dyggve-melchior-clausen syndrome protein
-2.9723	0.0075797	T1G11.7	Pectin lyase-like superfamily protein

-4.1131	0.040019	WEE1	WEE1-like kinase
1.0123	0.0026483	SCAMP4	Secretory carrier membrane protein (SCAMP) family protein
-3.6198	0.028386	CSLD5	cellulose synthase-like D5
-2.5464	0.030706	XIA	myosin XI A
-1.7429	0.013605	T6A9.34	protoporphyrinogen oxidase-like protein
-1.3014	0.034087	AtBRN2	RNA-binding (RRM/RBD/RNP motifs) family protein
-1.4614	0.0030263	T1G11.21	Calcium-dependent lipid-binding (CaLB domain) family protein
-3.8675	0.045533	PDLP2	plasmodesmata-located protein 2
-1.0464	0.0031437	F19P19.11	S-adenosyl-L-methionine-dependent methyltransferases superfamily protein
-2.1885	0.029773	GPAT2	glycerol-3-phosphate acyltransferase 2
-1.8782	4.12E-06	SHY2	AUX/IAA transcriptional regulator family protein
2.0045	0.028425	T7N9.8	plant/protein
1.1501	0.0087953	T7N9.23	transmembrane receptors / ATP binding protein
-1.449	0.025311	T7N9.27	ARM repeat superfamily protein
-1.5195	0.022869	NPGR1	no pollen germination related 1
0.83335	0.022884	F13K9.4	glucuronoxylan 4-O-methyltransferase-like protein (DUF579)
-2.6861	6.99E-08	LTPG1	glycosylphosphatidylinositol-anchored lipid protein transfer 1
0.88641	0.034825	BBX13	B-box type zinc finger protein with CCT domain-containing protein
1.0477	0.0020114	PUP1	purine permease 1
-2.7936	0.0048489	AGP31	arabinogalactan protein 31
1.8358	0.019457	WRKY65	WRKY DNA-binding protein 65
-2.8631	0.00080416	F15D2.21	GDSL-like Lipase/Acylhydrolase superfamily protein
-1.7931	3.07E-09	F15D2.22	GDSL-like Lipase/Acylhydrolase superfamily protein
-1.3362	0.00068538	RWA4	O-acetyltransferase family protein
-2.2049	0.011803	CAB2	chlorophyll A/B-binding protein 2
-2.0497	2.57E-06	T1P2.9	choice-of-anchor C domain protein%2C putative (Protein of unknown function%2C DUF642)
1.3521	0.00014642	F12P21.4	proline-%2C glutamic acid/leucine-rich protein
1.5036	0.032328	F12P21.5	hypothetical protein
-0.93993	0.032528	HERK2	hercules receptor kinase 2
-1.6804	0.016997	VSR6	VACUOLAR SORTING RECEPTOR 6
1.4349	0.0017443	F5M6.24	proline-rich family protein
-2.2512	0.00053525	PRR1	pinoresinol reductase 1
1.0005	0.049359	NRT1.5	nitrate transporter 1.5
1.0577	0.019558	F6N18.12	SBP (S-ribonuclease binding protein) family protein
-2.0001	0.046883	T9L6.6	S-adenosyl-L-methionine-dependent methyltransferases superfamily protein
-1.0657	0.0020114	F10C21.14	RNA-binding (RRM/RBD/RNP motifs) family protein
-3.1939	5.54E-07	F10C21.23	RING/U-box superfamily protein
-1.2361	0.039949	T1E4.10	Leucine-rich repeat (LRR) family protein
1.3825	0.012391	F12G12.12	Pyridoxal phosphate (PLP)-dependent transferases superfamily protein
-1.67	0.044213	SAMC2	S-adenosylmethionine carrier 2
-1.1107	0.027659	F12K21.25	leucine-rich repeat transmembrane protein kinase family protein
-3.1659	6.55E-18	PHI-1	Phosphate-responsive 1 family protein
-2.9281	0.0017443	MLP165	MLP-like protein 165
-1.7129	1.05E-09	F14D7.1	kinase family with leucine-rich repeat domain-containing protein
-1.1653	7.90E-05	ANNAT1	annexin 1
-1.1569	6.83E-05	SHM7	serine hydroxymethyltransferase 7
-2.1844	0.023418	T32E20.35	myotubularin-like protein
1.573	0.045595	HCS2	hypothetical protein

-3.0406	0.0020114	SKS6	SKU5-similar 6
3.0896	0.026098	TLP5	tubby like protein 5
-1.3823	0.027185	F9C16.7	P-loop containing nucleoside triphosphate hydrolases superfamily protein
-2.9169	3.31E-06	MCM2	minichromosome maintenance (MCM2/3/5) family protein
-2.8163	0.0073226	F27F5.9	TRAM%2C LAG1 and CLN8 (TLC) lipid-sensing domain containing protein
-2.6605	7.76E-12	BGAL5	hypothetical protein
-1.3572	0.0005906	F2G19.25	transmembrane protein
-1.8454	0.049359	CYCA3%3B2	cyclin-dependent protein kinase 3%3B2
-1.3003	0.0063221	3BETAHSD/D[illegible]	3beta-hydroxysteroid-dehydrogenase/decarboxylase isoform 1
-1.3152	0.0085816	T3F24.2	Protein phosphatase 2C family protein
-1.8277	0.0046169	F11A17.16	hydroxyproline-rich glycoprotein family protein
-3.9212	0.024	RKL1	receptor-like kinase 1
-1.459	0.00012999	BBX17	B-box type zinc finger protein with CCT domain-containing protein
-1.9315	0.007426	LACS2	long-chain acyl-CoA synthetase 2
-1.8339	0.022884	F14J22.18	Calcium-dependent protein kinase (CDPK) family protein
-2.2097	0.00013617	F14J22.6	Protein kinase superfamily protein
-1.3164	0.027659	F14J22.4	Leucine-rich repeat (LRR) family protein
-0.90642	0.045524	F10F5.2	glutamyl-tRNA (Gln) amidotransferase subunit A (DUF620)
-2.1938	0.0029246	TUA2	tubulin alpha-2 chain
-2.1013	0.0022609	F4M15.2	aminotransferase-like%2C mobile domain protein
0.81516	0.047696	F23H24.15	Heavy metal transport/detoxification superfamily protein
0.80655	0.015999	F11M15.13	vesicle-associated protein 1-4
-0.7989	0.013605	ABCG12	ABC-2 type transporter family protein
-1.5952	2.73E-05	AtNPF1.2	Major facilitator superfamily protein
-1.0909	0.0095641	ABA2	NAD(P)-binding Rossmann-fold superfamily protein
-6.0302	0.013316	F14G24.2	alpha/beta-Hydrolases superfamily protein
-6.4937	1.77E-05	F14G24.4	Phototropic-responsive NPH3 family protein
-3.4613	2.44E-05	IAA6	indole-3-acetic acid 6
-3.3936	0.00041981	F8L10.7	Legume lectin family protein
-3.9933	0.013141	FAP3	Chalcone-flavanone isomerase family protein
0.86435	0.013999	T18A20.2	26S proteasome regulatory complex ATPase
-0.95783	0.0030886	PME1	pectin methylesterase 1
-3.248	1.26E-05	F20D21.27	Calcium-binding EF-hand family protein
-1.847	0.01271	T22H22.21	Protein kinase superfamily protein
-3.6173	0.047192	F7A10.16	Bifunctional inhibitor/lipid-transfer protein/seed storage 2S albumin superfamily protein
-0.87957	0.016953	F7A10.1	tRNA-splicing ligase (DUF239)
-0.81879	0.011393	T5A14.14	S-adenosyl-L-methionine-dependent methyltransferases superfamily protein
-1.0128	0.0018805	T5A14.7	RING/U-box superfamily protein
3.4214	0.012565	F20N2.9	FBD%2C F-box and Leucine Rich Repeat domains containing protein
-0.93392	0.0091203	F25P12.102	Disease resistance protein (TIR-NBS-LRR class) family
-1.4179	0.015976	F25P12.101	Disease resistance protein (TIR-NBS-LRR class) family
-3.4029	0.0017264	RGXT3	RhamnoGalacturonan specific Xylosyltransferase 1
-2.4717	7.28E-12	F25P12.84	Protein kinase superfamily protein
-2.7658	4.85E-05	VIM1	Zinc finger (C3HC4-type RING finger) family protein
-1.555	0.00083003	PUP18	purine permease 18
-2.4161	0.027659	JAX1	jacalin lectin-like protein
-2.3953	0.015625	AT1G58225	hypothetical protein
0.82839	0.025311	AT1G58235	hypothetical protein
-2.9285	0.031859	ZW9	TRAF-like family protein
-1.1179	0.028501	HEMA1	Glutamyl-tRNA reductase family protein
-5.876	0.011658	AT1G58390	Disease resistance protein (CC-NBS-LRR class) family

-0.93642	0.0093661	XF1	FAD/NAD(P)-binding oxidoreductase family protein
2.7524	0.041203	GSTU15	glutathione S-transferase TAU 15
-0.85597	0.013574	F23H11.22	Actin-binding FH2 (formin homology 2) family protein
-1.4628	0.019253	F23H11.28	Matrixin family protein
-2.5321	4.59E-05	T2K10.11	Serine/threonine-protein kinase WNK (With No Lysine)-like protein
-3.1649	0.008986	PG1	polygalacturonase 1
-1.1357	0.037802	TBL2	trichome birefringence-like protein (DUF828)
-2.2641	0.047648	F11P17.11	hypothetical protein
-2.5204	0.00057566	LRX2	leucine-rich repeat/extensin 2
-3.201	1.23E-06	T3P18.8	sulfated surface-like glycoprotein
-1.0921	0.00080168	F23N19.16	Bifunctional inhibitor/lipid-transfer protein/seed storage 2S albumin superfamily protein
1.1959	0.0026301	CuAO1	Copper amine oxidase family protein
-5.1367	0.029233	F16P17.2	hypothetical protein
-5.1667	0.024123	F16M19.21	GRAS family transcription factor
-2.3914	2.22E-05	TET10	tetraspanin10
1.9702	0.04057	F2K11.11	hypothetical protein
1.5113	5.95E-07	F15H21.8	hypothetical protein
-1.2168	0.00015535	LACS3	AMP-dependent synthetase and ligase family protein
-2.0999	5.37E-05	ENODL8	early nodulin-like protein 8
-3.0945	1.10E-07	BDG1	alpha/beta-Hydrolases superfamily protein
-1.5092	0.0033881	CDI	Nucleotide-diphospho-sugar transferases superfamily protein
-1.477	0.029371	F13O11.30	WEB family protein (DUF827)
#NAME?	0.006113	GLC	HXXXD-type acyl-transferase family protein
-0.84648	0.027659	RK1	receptor kinase 1
-1.1926	0.047507	F12P19.9	Regulator of chromosome condensation (RCC1) family with FYVE zinc finger domain-containing protein
-1.914	1.49E-06	F15E12.12	hikeshi-like protein
-3.4436	0.00037693	F15E12.17	Disease resistance protein (TIR-NBS class)
-1.6474	0.02059	F15E12.7	Eukaryotic aspartyl protease family protein
-1.2998	0.0020425	F28G11.11	pfkB-like carbohydrate kinase family protein
-2.3156	0.048092	T4O24.1	kinase-like protein
-1.7255	0.019429	LUP5	Terpenoid cyclases family protein
-1.2869	9.19E-05	F1O19.11	membrane-associated kinase regulator
-5.0686	0.0053983	F5A8.9	zinc finger (C3HC4-type RING finger) family protein / BRCT domain-containing protein
-1.2437	0.0078395	EMB2813	DNA primase%2C large subunit family
-2.7608	5.88E-06	POLA2	DNA polymerase alpha 2
-4.231	0.020088	F12A21.12	Pectate lyase family protein
-1.4291	0.002349	FXG1	alpha-fucosidase 1
0.91725	0.0041965	T23K23.29	transmembrane protein
-2.1966	1.94E-06	T23K23.20	RNA-binding (RRM/RBD/RNP motifs) family protein
-0.93243	0.008773	MAP70-1	microtubule-associated proteins 70-1
-3.8676	0.012517	T22E19.4	membrane-associated kinase regulator
-3.8724	0.00010688	T26J14.4	Exostosin family protein
0.8788	0.02318	F24J5.16	aluminum activated malate transporter family protein
-1.4589	0.035352	F14K14.11	RNI-like superfamily protein
-3.7764	3.15E-06	F4N2.5	Adenine nucleotide alpha hydrolases-like superfamily protein
-1.3811	0.022422	F10D13.9	DHHC-type zinc finger family protein
-1.0053	0.016011	TCP15	TCP family transcription factor
-1.9717	0.0007735	HVA22C	HVA22 homologue C
-1.7628	0.049447	CMT3	chromomethylase 3
-2.1575	0.00030011	LGT8	glucosyl transferase family 8
-3.5916	5.88E-06	CYCD1%3B1	CYCLIN D1%3B1
-2.0374	0.00088643	PG2	polygalacturonase 2

-4.3332	0.00022672	MLP28	MLP-like protein 28
4.91	0.012391	MLP34	MLP-like protein 34
-1.4213	0.021985	CLE17	CLAVATA3/ESR-RELATED 17
-1.237	3.89E-05	PIN3	Auxin efflux carrier family protein
2.5703	0.041913	F3I17.7	DEA(D/H)-box RNA helicase family protein
1.6153	0.0044897	RLP12	receptor like protein 12
3.6097	9.53E-05	F26A9.11	Integrase-type DNA-binding superfamily protein
-3.0679	0.014668	T9N14.5	hypothetical protein
-1.3434	0.0011404	F28P22.2	hydroxyproline-rich glycoprotein family protein
-1.6934	0.0031976	HTH	Glucose-methanol-choline (GMC) oxidoreductase family protein
-1.4789	0.0059128	F3N23.22	anoctamin-like protein
-2.0784	3.13E-06	SARD1	Calmodulin binding protein-like protein
-1.1645	0.033707	F2P9.24	Bifunctional inhibitor/lipid-transfer protein/seed storage 2S albumin superfamily protein
-2.7039	3.28E-15	HSP101	heat shock protein 101
-0.78435	0.041203	XXT5	xyloglucan xylosyltransferase 5
-1.7427	2.76E-05	F1M20.12	ER membrane protein%2C putative (DUF962)
-1.3381	0.033419	IQD31	IQ-domain 31
-2.7431	0.01427	PR5	pathogenesis-related protein 5
-4.9173	4.19E-06	F22H5.12	DNA ligase-like protein
-1.2701	0.0031361	F22H5.9	Erythronate-4-phosphate dehydrogenase family protein
-1.6927	0.036567	HB33	homeobox protein 33
-2.1743	0.0019904	GH9B7	glycosyl hydrolase 9B7
-1.2239	0.011995	F10A5.10	C2H2-like zinc finger protein
-5.3386	0.012473	TUB1	tubulin beta-1 chain
-1.9423	4.77E-07	T4O12.13	GDSL-like Lipase/Acylhydrolase superfamily protein
-2.2412	0.048092	sks5	SKU5 similar 5
-2.2184	0.006172	CDKB2%3B1	cyclin-dependent kinase B2%3B1
1.3216	0.037516	AT1G76705	calmodulin binding protein
-3.2691	0.019698	T14N5.12	hypothetical protein
-0.94502	0.019088	LACS9	long chain acyl-CoA synthetase 9
-4.3719	0.00076532	T5M16.23	Integrase-type DNA-binding superfamily protein
-1.3862	0.0032303	LAX3	like AUX1 3
0.90814	0.013882	TGA7	bZIP transcription factor family protein
-0.98322	0.001745	F28K19.26	Pollen Ole e 1 allergen and extensin family protein
-0.91431	0.038483	CYP708A3	cytochrome P450%2C family 708%2C subfamily A%2C polypeptide 3
-0.92806	0.0064028	RHM1	rhamnose biosynthesis 1
1.6088	1.82E-05	F9K20.18	pathogenesis-related family protein
-3.4805	1.26E-23	F9K20.13	D-mannose binding lectin protein with Apple-like carbohydrate-binding domain-containing protein
-1.0965	0.0032067	F9K20.12	Curculin-like (mannose-binding) lectin family protein
-2.6053	0.017608	F9K20.10	D-mannose binding lectin protein with Apple-like carbohydrate-binding domain-containing protein
-1.701	0.02978	YUP8H12R.32	TPRXL
-1.7169	0.00027712	SGB1	Major facilitator superfamily protein
-2.9428	0.020495	F23A5.4	PPPDE putative thiol peptidase family protein
0.82713	0.016456	NRAMP1	natural resistance-associated macrophage protein 1
3.9772	1.71E-06	--	Strong similarity to a mitochondrial carrier protein from Ribes nigrum gb|AJ007580. It contains a mitochondrial carrier protein domain PF|00153. ESTs gb|T46775, gb|R90539, gb|AW029646 and gb|AA605443 come from this gene [Arabidopsis thaliana]
-0.95249	0.034066	--	T2E6.3 [Arabidopsis thaliana]>gi|110737661|dbj|BAF00770.1| hypothetical
-1.7897	3.42E-05	--	putative retroelement pol polyprotein [Arabidopsis thaliana]

1.6594	0.0048433	--	putative serpin [Arabidopsis thaliana]>gi|20197523|gb|AAM15110.1| putative serpin [Arabidopsis thaliana]
-2.9276	1.51E-07	--	hypothetical protein CARUB_v10015041mg [Capsella rubella]>gi|482565349|gb|EOA29538.1| hypothetical protein CARUB_v10015041mg [Capsella rubella]
-1.5466	5.70E-05	--	--
-2.8472	8.28E-05	--	--
1.3414	0.0036857	--	hypothetical protein VOLCADRAFT_65252 [Volvox carteri f. nagariensis]>gi|300259859|gb|EFJ44083.1| hypothetical protein VOLCADRAFT_65252, partial [Volvox carteri f. nagariensis]
2.9403	0.011995	--	serine/threonine kinase-like protein (fragment) [Arabidopsis thaliana]>gi|7321047|emb|CAB82155.1| serine/threonine kinase-like protein (fragment) [Arabidopsis thaliana]
0.7577	0.038013	--	protein MEI2-like 3 [Arabidopsis thaliana]>gi|240255989|ref|NP_001119005.4| protein MEI2-like 3 [Arabidopsis thaliana]>gi|332658598|gb|AEE83998.1| protein MEI2-like 3 [Arabidopsis thaliana]>gi|332658599|gb|AEE83999.1| protein MEI2-like 3 [Arabidopsis thaliana]
1.0361	0.019587	--	--
0.83346	0.016396	--	hypothetical protein [Arabidopsis thaliana]
