# Supplementary material for: NSs, the Silencing Suppressor of Tomato Spotted Wilt Orthotospovirus, Interferes With JA-Regulated Host Terpenoids Expression to Attract Frankliniella occidentalis
Source: Front Microbiol. 2020 Dec 10;11:590451. doi: 10.3389/fmicb.2020.590451 (PMC7758462; doi:10.3389/fmicb.2020.590451)
Supplement: Supplementary file 5 [file Data_Sheet_5.PDF]

Table S4 DEGs KEGG pathway enrichment result. (Pathways related to terpenoid biosynthesis, plant hormone signal transducion, plant-pathogen interacion were written in bold)

Term	ID	Input number	Input	FDR(NSs vs WT)	KEGG_ID/KO	Entrez_ID	Hyperlink
DNA replication	ath03030	15	815415|837249|816142|841056|838985|836845|824429|834492|836221|828153|843086|817048|843052|834193|830696	2.31E-05	ath:AT2G07690|ath:AT1G07370|ath:AT2G16440|ath:AT1G44900|ath:AT1G23750|ath:AT5G67100|ath:AT3G52630|ath:AT5G44635|ath:AT5G61000|ath:AT4G02060|ath:AT1G67630|ath:AT2G25100|ath:AT1G67320|ath:AT5G41880|ath:AT5G08020|	815415|837249|816142|841056|838985|836845|824429|834492|836221|828153|843086|817048|843052|834193|830696|	http://www.genome.jp/kegg-bin/show_pathway?ath03030/ath:AT5G41880%09red/ath:AT3G52630%09red/ath:AT2G16440%09red/ath:AT2G07690%09red/ath:AT5G08020%09red/ath:AT5G61000%09red/ath:AT1G67630%09red/ath:AT5G67100%09red/ath:AT1G07370%09red/ath:AT5G44635%09red/ath:AT1G44900%09red/ath:AT1G67320%09red/ath:AT1G23750%09red/at
**Steroid biosynthesis**	**ath00100**	**7**	**829932|821519|831216|842213|829617|841132|823649**	**0.1272**	**ath:AT4G37760|ath:AT3G19820|ath:AT5G13710|ath:AT1G58440|ath:AT4G34650|ath:AT1G47290|ath:AT3G45130|**	**829932|821519|831216|842213|829617|841132|823649|**	**http://www.genome.jp/kegg-bin/show_pathway?ath00100/ath:AT3G19820%09red/ath:AT1G58440%09red/ath:AT3G45130%09red/ath:AT5G13710%09red/ath:AT4G34650%09red/ath:AT4G37760%0**
Pentose and glucuronate interconversions	ath00040	9	843100|824657|819886|839452|836439|824485|828064|820241|834948	0.34086	ath:AT1G67750|ath:AT3G54920|ath:AT3G07010|ath:AT1G04680|ath:AT5G63180|ath:AT3G53190|ath:AT4G02330|ath:AT3G10720|ath:AT5G48900|	843100|824657|819886|839452|836439|824485|828064|820241|834948|	http://www.genome.jp/kegg-bin/show_pathway?ath00040/ath:AT1G67750%09red/ath:AT3G07010%09red/ath:AT5G48900%09red/ath:AT4G02330%09red/ath:AT5G63180%09red/ath:AT3G10720%09red/ath:AT3G54920%09red/ath:AT3G53190%09red/

Photosynthesis - antenna proteins	ath00196	4	822391|815055|839870|815058	0.34086	ath:AT3G27690|ath:AT2G05070|ath:AT1G29920|ath:AT2G05100|	822391|815055|839870|815058|	http://www.genome.jp/kegg-bin/show_pathway?ath00196/ath:AT1G29920%09red/ath:AT2G05100%09red/ath:AT3G27690%09red/ath:AT2G05070%0
Phagosome	ath04145	9	825225|843911|817504|837365|839405|832451|834459|836391|841425	0.34086	ath:AT3G60540|ath:AT1G75780|ath:AT2G29550|ath:AT1G08450|ath:AT1G50010|ath:AT5G23860|ath:AT5G44340|ath:AT5G62700|ath:AT1G50010|	825225|843911|817504|837365|841425|832451|834459|836391|841425|	http://www.genome.jp/kegg-bin/show_pathway?ath04145/ath:AT3G60540%09red/ath:AT5G23860%09red/ath:AT1G08450%09red/ath:AT1G75780%09red/ath:AT1G50010%09red/ath:AT5G62700%09red/ath:AT5G44340%09red/ath
Cutin, suberine and wax biosynthesis	ath00073	4	842857|829521|828019|843628	0.34086	ath:AT5G41040|ath:AT4G33790|ath:AT4G00360|ath:AT1G72970|	834106|829521|828019|843628|	http://www.genome.jp/kegg-bin/show_pathway?ath00073/ath:AT4G33790%09red/ath:AT1G72970%09red/ath:AT4G00360%09red/ath:AT5G41040%0
Vitamin B6 metabolism	ath00750	3	836175|838347|820850	0.34086	ath:AT5G60540|ath:AT1G17710|ath:AT3G16050|	836175|838347|820850|	http://www.genome.jp/kegg-bin/show_pathway?ath00750/ath:AT3G16050%09red/ath:AT5G60540%09red/ath:AT1G17710%09re
ABC transporters	ath02010	4	822519|839353|828702|822463	0.34086	ath:AT3G28860|ath:AT1G02520|ath:AT4G25960|ath:AT3G28345|	822519|839353|828702|822463|	http://www.genome.jp/kegg-bin/show_pathway?ath02010/ath:AT3G28345%09red/ath:AT4G25960%09red/ath:AT1G02520%09red/ath:AT3G28860%0
Tropane, piperidine and pyridine alkaloid biosynthesis	ath00960	5	842580|817479|817483|833613|826838	0.34086	ath:AT1G62810|ath:AT2G29300|ath:AT2G29320|ath:AT5G36160|ath:AT4G12290|	842580|817479|817481|833613|826838|	http://www.genome.jp/kegg-bin/show_pathway?ath00960/ath:AT4G12290%09red/ath:AT1G62810%09red/ath:AT2G29300%09red/ath:AT2G29320%09red/ath:AT5G3616

Circadian rhythm - plant	ath04712	5	832518|830730|830996|838883|836132	0.34086	ath:AT5G24470|ath:AT5G08330|ath:AT5G11260|ath:AT1G22770|ath:AT5G60100|	832518|830730|830996|838883|836132|	http://www.genome.jp/kegg-bin/show_pathway?ath04712/ath:AT5G11260%09red/ath:AT5G08330%09red/ath:AT5G60100%09red/ath:AT5G24470%09red/ath:AT1G2277
**Plant-pathogen interaction**	**ath04626**	**14**	**823963|827089|818710|815949|827114|818709|835701|824355|817165|828457|835699|839395|829179|828713**	**0.34086**	**ath:AT3G48080|ath:AT4G14440|ath:AT3G56800|ath:AT2G14610|ath:AT4G14640|ath:AT2G41100|ath:AT5G56010|ath:AT3G51920|ath:AT2G26250|ath:AT4G23570|ath:AT5G56010|ath:AT1G01120|ath:AT4G30**	**823963|827089|824847|815949|827114|818709|835699|824355|817165|828457|835699|839395|829179|828713|**	**http://www.genome.jp/kegg-bin/show_pathway?ath04626/ath:AT2G26250%09red/ath:AT2G41100%09red/ath:AT3G48080%09red/ath:AT3G51920%09red/ath:AT4G23570%09red/ath:AT4G30560%09red/ath:AT4G14440%09red/ath:AT2G14610%09red/ath:AT5G56010%09red/ath:AT4G26070%09red/ath:AT3G56**
Phenylpropanoid biosynthesis	ath00940	13	830088|817967|835504|835264|835952|818280|817599|834951|818289|839196|823854|829779|825183	0.34086	ath:AT4G39330|ath:AT2G34060|ath:AT5G54160|ath:AT5G51890|ath:AT5G58390|ath:AT2G37040|ath:AT2G30490|ath:AT5G48930|ath:AT2G37130|ath:AT1G26560|ath:AT3G47010|ath:AT4G36220|ath:AT3G60130|	830088|817967|835504|835264|835952|818280|817599|834951|818289|839196|823854|829779|825183|	http://www.genome.jp/kegg-bin/show_pathway?ath00940/ath:AT2G30490%09red/ath:AT5G54160%09red/ath:AT5G48930%09red/ath:AT2G37130%09red/ath:AT5G58390%09red/ath:AT3G47010%09red/ath:AT5G51890%09red/ath:AT4G39330%09red/ath:AT2G37040%09red/ath:AT4G36220%09red/ath:AT3G60130%09red/ath:AT1G
Mismatch repair	ath03430	5	838985|836221|824429|830696|837249	0.34086	ath:AT1G23750|ath:AT5G61000|ath:AT3G52630|ath:AT5G08020|ath:AT1G07370|	838985|836221|824429|830696|837249|	http://www.genome.jp/kegg-bin/show_pathway?ath03430/ath:AT1G23750%09red/ath:AT3G52630%09red/ath:AT1G07370%09red/ath:AT5G08020%09red/ath:AT5G6100

Starch and sucrose metabolism	ath00500	15	818212|827959|842961|839467|829167|835220|821965|828064|820241|839196|821499|823854|833692|825183|28721286	0.34086	ath:AT2G36390|ath:AT4G00490|ath:AT1G66430|ath:AT1G02730|ath:AT4G30440|ath:AT5G51460|ath:AT3G23820|ath:AT4G02330|ath:AT3G10720|ath:AT1G26560|ath:AT3G19620|ath:AT3G47010|ath:AT5G37180|ath:AT3G60130|ath:AT4G02280|	818212|827959|842961|839467|829167|835220|821965|828064|820241|839196|821499|823854|833692|825183|828081|	http://www.genome.jp/kegg-bin/show_pathway?ath00500/ath:AT2G36390%09red/ath:AT1G66430%09red/ath:AT1G02730%09red/ath:AT4G02330%09red/ath:AT3G23820%09red/ath:AT5G51460%09red/ath:AT3G47010%09red/ath:AT3G10720%09red/ath:AT4G02280%09red/ath:AT5G37180%09red/ath:AT4G30440%09red/ath:AT3G60130%09red/ath:AT1G26560%09red/at
Fatty acid biosynthesis	ath00061	5	821961|831500|841367|844094|842748	0.34086	ath:AT3G23790|ath:AT5G16390|ath:AT1G49430|ath:AT1G77590|ath:AT1G64400|	821961|831500|841367|844094|842748|	http://www.genome.jp/kegg-bin/show_pathway?ath00061/ath:AT5G16390%09red/ath:AT1G64400%09red/ath:AT3G23790%09red/ath:AT1G77590%09red/ath:AT1G4943
**Plant hormone signal transduction**	**ath04075**	**20**	**818390|839570|835860|838812|819281|820145|3770664|820793|816659|815949|844127|816640|817247|821879|829029|844105|838518|841717|831177|81470**	**0.34086**	**ath:AT2G38120|ath:AT1G04240|ath:AT5G57560|ath:AT1G22070|ath:AT2G46690|ath:AT3G09870|ath:AT5G18010|ath:AT3G15540|ath:AT2G21220|ath:AT2G14610|ath:AT1G77920|ath:AT2G21050|ath:AT2G27050|ath:AT3G23050|ath:AT4G29080|ath:AT1G77690|ath:AT1G19350|ath:AT1G52830|**	**818390|839570|835860|838812|819281|820145|831668|820793|816659|815949|844127|816640|817247|821879|829029|844105|838518|841717|831177|814707|**	**http://www.genome.jp/kegg-bin/show_pathway?ath04075/ath:AT1G77920%09red/ath:AT5G18010%09red/ath:AT2G27050%09red/ath:AT1G77690%09red/ath:AT3G15540%09red/ath:AT2G46690%09red/ath:AT2G14610%09red/ath:AT1G04240%09red/ath:AT2G21050%09red/ath:AT2G38120%09red/ath:AT1G19350%09red/ath:AT5G13360%09red/ath:AT3G09870%09red/ath:AT2G21220%09red/ath:AT3G23050%09red/ath:AT1G52830%09red/**

Cysteine and methionine metabolism	ath00270	9	829933|819708|821003|826317|818260|822186|833613|832873|843313	0.3504	ath:AT4G37770|ath:AT3G05430|ath:AT3G17390|ath:AT4G08040|ath:AT2G36880|ath:AT3G25900|ath:AT5G36160|ath:AT5G28030|ath:AT1G69770|	829933|819708|821003|826317|818260|822186|833613|832873|843313|	http://www.genome.jp/kegg-bin/show_pathway?ath00270/ath:AT2G36880%09red/ath:AT5G36160%09red/ath:AT4G08040%09red/ath:AT5G28030%09red/ath:AT3G25900%09red/ath:AT3G17390%09red/ath:AT3G05430%09red/ath:AT1G69770%09red/
Homologous recombination	ath03440	6	836221|836513|838985|824429|830696|819743	0.3504	ath:AT5G61000|ath:AT5G63920|ath:AT1G23750|ath:AT3G52630|ath:AT5G08020|ath:AT3G05740|	836221|836513|838985|824429|830696|819743|	http://www.genome.jp/kegg-bin/show_pathway?ath03440/ath:AT3G52630%09red/ath:AT5G08020%09red/ath:AT5G61000%09red/ath:AT3G05740%09red/ath:AT1G23750%09red/ath:AT5G6
Other glycan degradation	ath00511	3	817355|843109|829575	0.3504	ath:AT2G28100|ath:AT1G67830|ath:AT4G34260|	817355|843109|829575|	http://www.genome.jp/kegg-bin/show_pathway?ath00511/ath:AT2G28100%09red/ath:AT4G34260%09red/ath:AT1G67830%09re
Fatty acid metabolism	ath01212	7	817548|844094|821961|842748|841367|837117|831500	0.3762	ath:AT2G29980|ath:AT1G77590|ath:AT3G23790|ath:AT1G64400|ath:AT1G49430|ath:AT1G06080|ath:AT5G16390|	817548|844094|821961|842748|841367|837117|831500|	http://www.genome.jp/kegg-bin/show_pathway?ath01212/ath:AT2G29980%09red/ath:AT1G49430%09red/ath:AT5G16390%09red/ath:AT1G64400%09red/ath:AT3G23790%09red/ath:AT1G06080%09red/ath:AT
Cyanoamino acid metabolism	ath00460	6	840543|832290|839196|829910|823854|825183	0.38965	ath:AT1G36370|ath:AT5G22300|ath:AT1G26560|ath:AT4G37550|ath:AT3G47010|ath:AT3G60130|	840543|832290|839196|829910|823854|825183|	http://www.genome.jp/kegg-bin/show_pathway?ath00460/ath:AT1G26560%09red/ath:AT5G22300%09red/ath:AT3G47010%09red/ath:AT3G60130%09red/ath:AT1G36370%09red/ath:AT4G3
**Sesquiterpenoid and triterpenoid biosynthesis**	**ath00909**	**3**	**829932|842213|829617**	**0.47709**	**ath:AT4G37760|ath:AT1G58440|ath:AT4G34650|**	**829932|842213|829617|**	**http://www.genome.jp/kegg-bin/show_pathway?ath00909/ath:AT4G34650%09red/ath:AT4G37760%09red/ath:AT1G58440**

Isoquinoline alkaloid biosynthesis	ath00950	3	842580|833613|826838	0.47709	ath:AT1G62810|ath:AT5G36160|ath:AT4G12290|	842580|833613|826838|	http://www.genome.jp/kegg-bin/show_pathway?ath00950/ath:AT4G12290%09red/ath:AT1G62810%09red/ath:AT5G36160%09re
Phenylalanine metabolism	ath00360	9	817967|835264|842580|835952|817599|818289|833613|826838|818280	0.48065	ath:AT2G34060|ath:AT5G51890|ath:AT1G62810|ath:AT5G58390|ath:AT2G30490|ath:AT2G37130|ath:AT5G36160|ath:AT4G12290|ath:AT2G37040|	817967|835264|842580|835952|817599|818289|833613|826838|818280|	http://www.genome.jp/kegg-bin/show_pathway?ath00360/ath:AT2G30490%09red/ath:AT1G62810%09red/ath:AT5G36160%09red/ath:AT5G58390%09red/ath:AT4G12290%09red/ath:AT5G51890%09red/ath:AT2G37040%09red/ath:AT2G34060%09red/
Nucleotide excision repair	ath03420	6	836221|837249|838564|838985|824429|830696	0.51427	ath:AT5G61000|ath:AT1G07370|ath:AT1G19750|ath:AT1G23750|ath:AT3G52630|ath:AT5G08020|	836221|837249|838564|838985|824429|830696|	http://www.genome.jp/kegg-bin/show_pathway?ath03420/ath:AT3G52630%09red/ath:AT5G08020%09red/ath:AT5G61000%09red/ath:AT1G07370%09red/ath:AT1G23750%09red/ath:AT1G1
Fatty acid degradation	ath00071	4	821961|844094|841367|842748	0.51621	ath:AT3G23790|ath:AT1G77590|ath:AT1G49430|ath:AT1G64400|	821961|844094|841367|842748|	http://www.genome.jp/kegg-bin/show_pathway?ath00071/ath:AT1G64400%09red/ath:AT3G23790%09red/ath:AT1G77590%09red/ath:AT1G49430%0
Ether lipid metabolism	ath00565	3	815261|819730|824021	0.51621	ath:AT2G06925|ath:AT3G05630|ath:AT3G48610|	815261|819730|824021|	http://www.genome.jp/kegg-bin/show_pathway?ath00565/ath:AT3G05630%09red/ath:AT3G48610%09red/ath:AT2G06925%09re
Nicotinate and nicotinamide metabolism	ath00760	2	832129|825377	0.55367	ath:AT5G20070|ath:AT3G62040|	832129|825377|	http://www.genome.jp/kegg-bin/show_pathway?ath00760/ath:AT5G20070%09red/ath:AT3G62040%09red

Base excision repair	ath03410	4	820453|834497|835908|837249	0.55912	ath:AT3G12710|ath:AT5G44680|ath:AT5G57970|ath:AT1G07370|	820453|834497|835908|837249|	http://www.genome.jp/kegg-bin/show_pathway?ath03410/ath:AT1G07370%09red/ath:AT5G44680%09red/ath:AT3G12710%09red/ath:AT5G57970%0
Carotenoid biosynthesis	ath00906	3	841665|834570|827663	0.57051	ath:AT1G52340|ath:AT5G45340|ath:AT4G19230|	841665|834570|827663|	http://www.genome.jp/kegg-bin/show_pathway?ath00906/ath:AT5G45340%09red/ath:AT4G19230%09red/ath:AT1G52340%09re
Fatty acid elongation	ath00062	3	817165|839395|827089	0.58702	ath:AT2G26250|ath:AT1G01120|ath:AT4G14440|	817165|839395|827089|	http://www.genome.jp/kegg-bin/show_pathway?ath00062/ath:AT2G26250%09red/ath:AT4G14440%09red/ath:AT1G01120%09re
Arachidonic acid metabolism	ath00590	2	815261|831196	0.58702	ath:AT2G06925|ath:AT5G13520|	815261|831196|	http://www.genome.jp/kegg-bin/show_pathway?ath00590/ath:AT5G13520%09red/ath:AT2G06925%09red
**Ubiquinone and other terpenoid-quinone biosynthesis**	**ath00130**	**3**	**817599|829413|833613**	**0.58702**	**ath:AT2G30490|ath:AT4G32770|ath:AT5G36160|**	**817599|829413|833613|**	**http://www.genome.jp/kegg-bin/show_pathway?ath00130/ath:AT2G30490%09red/ath:AT4G32770%09red/ath:AT5G36160**
Peroxisome	ath04146	6	832129|844094|842748|829521|841367|821961	0.58702	ath:AT5G20070|ath:AT1G77590|ath:AT1G64400|ath:AT4G33790|ath:AT1G49430|ath:AT3G23790|	832129|844094|842748|829521|841367|821961|	http://www.genome.jp/kegg-bin/show_pathway?ath04146/ath:AT4G33790%09red/ath:AT1G49430%09red/ath:AT1G64400%09red/ath:AT3G23790%09red/ath:AT5G20070%09red/ath:AT1G7
Pyrimidine metabolism	ath00240	8	831309|836845|843086|832710|819971|823847|834193|843052	0.58702	ath:AT5G14580|ath:AT5G67100|ath:AT1G67630|ath:AT5G26667|ath:AT3G07800|ath:AT3G46940|ath:AT5G41880|ath:AT1G67320|	831309|836845|843086|832710|819971|823847|834193|843052|	http://www.genome.jp/kegg-bin/show_pathway?ath00240/ath:AT5G41880%09red/ath:AT5G14580%09red/ath:AT3G07800%09red/ath:AT1G67630%09red/ath:AT5G67100%09red/ath:AT5G26667%09red/ath:AT1G67320%09red/ath

Inositol phosphate metabolism	ath00562	5	835981|830788|838014|819987|824021	0.58702	ath:AT5G58670|ath:AT5G09290|ath:AT1G14520|ath:AT3G07960|ath:AT3G48610|	835981|830788|838014|819987|824021|	http://www.genome.jp/kegg-bin/show_pathway?ath00562/ath:AT1G14520%09red/ath:AT3G48610%09red/ath:AT5G09290%09red/ath:AT5G58670%09red/ath:AT3G0796
Glycerophospholipid metabolism	ath00564	6	819730|839558|824021|828140|827165|815261	0.5929	ath:AT3G05630|ath:AT1G02390|ath:AT3G48610|ath:AT4G00400|ath:AT4G01950|ath:AT2G06925|	819730|839558|824021|828140|827165|815261|	http://www.genome.jp/kegg-bin/show_pathway?ath00564/ath:AT3G48610%09red/ath:AT4G00400%09red/ath:AT1G02390%09red/ath:AT3G05630%09red/ath:AT2G06925%09red/ath:AT4G0
Amino sugar and nucleotide sugar metabolism	ath00520	8	839467|844193|829167|823908|842961|818066|821499|821965	0.64008	ath:AT1G02730|ath:AT1G78570|ath:AT4G30440|ath:AT3G47540|ath:AT1G66430|ath:AT2G35020|ath:AT3G19620|ath:AT3G23820|	839467|844193|829167|823908|842961|818066|821499|821965|	http://www.genome.jp/kegg-bin/show_pathway?ath00520/ath:AT1G66430%09red/ath:AT1G02730%09red/ath:AT3G47540%09red/ath:AT1G78570%09red/ath:AT4G30440%09red/ath:AT2G35020%09red/ath:AT3G23820%09red/ath
One carbon pool by folate	ath00670	2	840543|819022	0.64008	ath:AT1G36370|ath:AT2G44160|	840543|819022|	http://www.genome.jp/kegg-bin/show_pathway?ath00670/ath:AT2G44160%09red/ath:AT1G36370%09red

Metabolic pathways	ath01100	98	837610|838847|837931|839558|836845|839870|818066|830867|829167|817483|828019|830788|830789|826838|823854|815261|835220|830985|832234|822186|841367|827165|835981|842961|820241	0.64761	ath:AT1G10670|ath:AT1G22410|ath:AT1G13700|ath:AT1G02390|ath:AT5G67100|ath:AT1G29920|ath:AT2G35020|ath:AT5G01320|ath:AT4G30440|ath:AT2G29320|ath:AT4G00360|ath:AT5G09290|ath:AT5G09300|ath:AT4G12290|ath:AT3G47010|ath:AT2G06925|ath:AT5G51460|ath:AT5G11160|ath:AT5G21100|ath:AT3G25900|ath:AT1G49430|ath:AT4G01950|ath:AT5G58670|ath	837610|838847|837931|839558|836845|839870|818066|830867|829167|817481|828019|830788|830789|826838|823854|815261|835220|830985|832234|822186|841367|827165|835981|842961|820241|819022|832873|823847|819730|826317|834951|821499|825183|828064|833692|829413|833613|830088|829933|829932|816244|821072|819971|842213|844094|842198|821003|815055|829779|815058|836625|841665|824592|8	http://www.genome.jp/kegg-bin/show_pathway?ath01100/ath:AT5G67100%09red/ath:AT5G09290%09red/ath:AT3G47010%09red/ath:AT4G39330%09red/ath:AT2G29300%09red/ath:AT1G10670%09red/ath:AT4G37770%09red/ath:AT1G66430%09red/ath:AT1G13700%09red/ath:AT5G58670%09red/ath:AT4G00400%09red/ath:AT1G67630%09red/ath:AT3G05430%09red/ath:AT3G25900%09red/ath:AT4G34650%09red/ath:AT1G67320%09red/ath:AT3G17390%09red/ath:AT2G37040%09red/ath:AT2G35020%09red/ath:AT2G30490%09red/ath:AT1G02390%09red/ath:AT1G094
Flavonoid biosynthesis	ath00941	2	817599|834951	0.64761	ath:AT2G30490|ath:AT5G48930|	817599|834951|	http://www.genome.jp/kegg-bin/show_pathway?ath00941/ath:AT2G30490%09red/ath:AT5G48930%09red

Biosynthesis of secondary metabolites	ath01110	53	837610|817967|835504|841665|838847|837931|824592|836625|826317|834951|818066|817165|830867|823649|825183|837465|821003|818212|831216|818383|835952|817483|842580|830789|833613	0.69157	ath:AT1G10670|ath:AT2G34060|ath:AT5G54160|ath:AT1G52340|ath:AT1G22410|ath:AT1G13700|ath:AT3G54250|ath:AT5G65010|ath:AT4G08040|ath:AT5G48930|ath:AT2G35020|ath:AT2G26250|ath:AT5G01320|ath:AT3G45130|ath:AT3G60130|ath:AT1G09420|ath:AT3G17390|ath:AT2G36390|ath:AT5G13710|ath:AT2G38050|ath:AT5G58390|ath:AT2G29320|ath:AT1G62810|ath	837610|817967|835504|841665|838847|837931|824592|836625|826317|834951|818066|817165|830867|823649|825183|837465|821003|818212|831216|818383|835952|817481|842580|830789|833613|826838|821519|823854|830088|829933|829932|816244|835264|840543|817599|818260|818289|822186|839196|817479|831500|835006|818280|842213|829617|844193|842198|823908|829413|832584|832585|839395|829779|	http://www.genome.jp/kegg-bin/show_pathway?ath01110/ath:AT5G54160%09red/ath:AT5G48930%09red/ath:AT5G36160%09red/ath:AT1G52340%09red/ath:AT5G16390%09red/ath:AT3G47540%09red/ath:AT3G17390%09red/ath:AT3G47010%09red/ath:AT1G78570%09red/ath:AT2G29300%09red/ath:AT1G10670%09red/ath:AT2G37130%09red/ath:AT4G37770%09red/ath:AT1G62810%09red/ath:AT3G19820%09red/ath:AT1G13700%09red/ath:AT3G45130%09red/ath:AT5G58390%09red/ath:AT4G12290%09red/ath:AT5G49460%09red/ath:AT1G01120%09red/ath:AT2G380
Tyrosine metabolism	ath00350	3	842580|833613|826838	0.69157	ath:AT1G62810|ath:AT5G36160|ath:AT4G12290|	842580|833613|826838|	http://www.genome.jp/kegg-bin/show_pathway?ath00350/ath:AT4G12290%09red/ath:AT1G62810%09red/ath:AT5G36160%09re
Brassinosteroid biosynthesis	ath00905	1	818383	0.71285	ath:AT2G38050|	818383|	http://www.genome.jp/kegg-bin/show_pathway?ath00905/ath:AT2G38050%09red
Nitrogen metabolism	ath00910	3	829910|821072|833738	0.71285	ath:AT4G37550|ath:AT3G03910|ath:AT5G37600|	829910|821072|833738|	http://www.genome.jp/kegg-bin/show_pathway?ath00910/ath:AT3G03910%09red/ath:AT5G37600%09red/ath:AT4G37550%09re
Degradation of aromatic compounds	ath01220	1	817599	0.71375	ath:AT2G30490|	817599|	http://www.genome.jp/kegg-bin/show_pathway?ath01220/ath:AT2G30490%09red
Linoleic acid metabolism	ath00591	1	815261	0.71375	ath:AT2G06925|	815261|	http://www.genome.jp/kegg-bin/show_pathway?ath00591/ath:AT2G06925%09red

Riboflavin metabolism	ath00740	1	838035	0.71375	ath:AT1G14700|	838035|	http://www.genome.jp/kegg-bin/show_pathway?ath00740/ath:AT1G14700%09red
Stilbenoid, diarylheptanoid and gingerol biosynthesis	ath00945	4	817599|834951|832584|832585	0.73433	ath:AT2G30490|ath:AT5G48930|ath:AT5G25130|ath:AT5G25140|	817599|834951|832584|832585|	http://www.genome.jp/kegg-bin/show_pathway?ath00945/ath:AT2G30490%09red/ath:AT5G25130%09red/ath:AT5G25140%09red/ath:AT5G48930%0
Phosphatidylinositol signaling system	ath04070	4	830788|835981|819987|818710	0.75641	ath:AT5G09290|ath:AT5G58670|ath:AT3G07960|ath:AT3G56800|	830788|835981|819987|824847|	http://www.genome.jp/kegg-bin/show_pathway?ath04070/ath:AT3G56800%09red/ath:AT5G09290%09red/ath:AT5G58670%09red/ath:AT3G07960%0
Alanine, aspartate and glutamate metabolism	ath00250	3	836625|821072|833738	0.75641	ath:AT5G65010|ath:AT3G03910|ath:AT5G37600|	836625|821072|833738|	http://www.genome.jp/kegg-bin/show_pathway?ath00250/ath:AT5G65010%09red/ath:AT3G03910%09red/ath:AT5G37600%09re
Thiamine metabolism	ath00730	1	817513	0.75641	ath:AT2G29630|	817513|	http://www.genome.jp/kegg-bin/show_pathway?ath00730/ath:AT2G29630%09red
Glycerolipid metabolism	ath00561	3	839558|828140|827165	0.8038	ath:AT1G02390|ath:AT4G00400|ath:AT4G01950|	839558|828140|827165|	http://www.genome.jp/kegg-bin/show_pathway?ath00561/ath:AT1G02390%09red/ath:AT4G01950%09red/ath:AT4G00400%09re
Fructose and mannose metabolism	ath00051	3	838689|837752|842961	0.8038	ath:AT1G20950|ath:AT1G12000|ath:AT1G66430|	838689|837752|842961|	http://www.genome.jp/kegg-bin/show_pathway?ath00051/ath:AT1G66430%09red/ath:AT1G12000%09red/ath:AT1G20950%09re
Biosynthesis of unsaturated fatty acids	ath01040	2	837117|817548	0.88024	ath:AT1G06080|ath:AT2G29980|	837117|817548|	http://www.genome.jp/kegg-bin/show_pathway?ath01040/ath:AT2G29980%09red/ath:AT1G06080%09red

beta-Alanine metabolism	ath00410	2	842580|826838	0.92246	ath:AT1G62810|ath:AT4G12290|	842580|826838|	http://www.genome.jp/kegg-bin/show_pathway?ath00410/ath:AT4G12290%09red/ath:AT1G62810%09red
Sulfur metabolism	ath00920	2	832873|830788	0.92246	ath:AT5G28030|ath:AT5G09290|	832873|830788|	http://www.genome.jp/kegg-bin/show_pathway?ath00920/ath:AT5G28030%09red/ath:AT5G09290%09red
Ascorbate and aldarate metabolism	ath00053	2	838014|832234	0.92246	ath:AT1G14520|ath:AT5G21100|	838014|832234|	http://www.genome.jp/kegg-bin/show_pathway?ath00053/ath:AT1G14520%09red/ath:AT5G21100%09red
Glyoxylate and dicarboxylate metabolism	ath00630	3	840543|829910|833738	0.92246	ath:AT1G36370|ath:AT4G37550|ath:AT5G37600|	840543|829910|833738|	http://www.genome.jp/kegg-bin/show_pathway?ath00630/ath:AT5G37600%09red/ath:AT1G36370%09red/ath:AT4G37550%09re
Glucosinolate biosynthesis	ath00966	1	838210	0.92246	ath:AT1G16400|	838210|	http://www.genome.jp/kegg-bin/show_pathway?ath00966/ath:AT1G16400%09red
Tryptophan metabolism	ath00380	2	832667|832669	0.94811	ath:AT5G25980|ath:AT5G26000|	832667|832669|	http://www.genome.jp/kegg-bin/show_pathway?ath00380/ath:AT5G26000%09red/ath:AT5G25980%09red
Glycine, serine and threonine metabolism	ath00260	3	840543|842580|826838	0.94811	ath:AT1G36370|ath:AT1G62810|ath:AT4G12290|	840543|842580|826838|	http://www.genome.jp/kegg-bin/show_pathway?ath00260/ath:AT4G12290%09red/ath:AT1G62810%09red/ath:AT1G36370%09re
Propanoate metabolism	ath00640	1	831500	0.94811	ath:AT5G16390|	831500|	http://www.genome.jp/kegg-bin/show_pathway?ath00640/ath:AT5G16390%09red
Glutathione metabolism	ath00480	4	819386|842257|837576|837465	0.94811	ath:AT2G47730|ath:AT1G59670|ath:AT1G10370|ath:AT1G09420|	819386|842257|837576|837465|	http://www.genome.jp/kegg-bin/show_pathway?ath00480/ath:AT1G10370%09red/ath:AT2G47730%09red/ath:AT1G59670%09red/ath:AT1G09420%0

Endocytosis	ath04144	5	838263|820102|819987|819730|829691	0.9487	ath:AT1G16920|ath:AT3G09440|ath:AT3G07960|ath:AT3G05630|ath:AT4G35380|	838263|820102|819987|819730|829691|	http://www.genome.jp/kegg-bin/show_pathway?ath04144/ath:AT3G05630%09red/ath:AT3G09440%09red/ath:AT1G16920%09red/ath:AT4G35380%09red/ath:AT3G0796
Protein export	ath03060	2	825225|838100	0.99817	ath:AT3G60540|ath:AT1G15310|	825225|838100|	http://www.genome.jp/kegg-bin/show_pathway?ath03060/ath:AT1G15310%09red/ath:AT3G60540%09red
Pentose phosphate pathway	ath00030	2	837931|837465	0.99817	ath:AT1G13700|ath:AT1G09420|	837931|837465|	http://www.genome.jp/kegg-bin/show_pathway?ath00030/ath:AT1G13700%09red/ath:AT1G09420%09red
Phenylalanine, tyrosine and tryptophan biosynthesis	ath00400	2	838847|833613	0.99966	ath:AT1G22410|ath:AT5G36160|	838847|833613|	http://www.genome.jp/kegg-bin/show_pathway?ath00400/ath:AT1G22410%09red/ath:AT5G36160%09red
**Terpenoid backbone biosynthesis**	**ath00900**	**2**	**816244|824592**	**0.99966**	**ath:AT2G17370|ath:AT3G54250|**	**816244|824592|**	**http://www.genome.jp/kegg-bin/show_pathway?ath00900/ath:AT3G54250%09red/ath:AT2G17370%09r**
Purine metabolism	ath00230	6	831309|836845|830985|843086|843052|834193	0.99966	ath:AT5G14580|ath:AT5G67100|ath:AT5G11160|ath:AT1G67630|ath:AT1G67320|ath:AT5G41880|	831309|836845|830985|843086|843052|834193|	http://www.genome.jp/kegg-bin/show_pathway?ath00230/ath:AT5G41880%09red/ath:AT5G14580%09red/ath:AT1G67630%09red/ath:AT5G67100%09red/ath:AT5G11160%09red/ath:AT1G6
alpha-Linolenic acid metabolism	ath00592	1	815261	0.99966	ath:AT2G06925|	815261|	http://www.genome.jp/kegg-bin/show_pathway?ath00592/ath:AT2G06925%09red
Citrate cycle (TCA cycle)	ath00020	2	837610|835006	0.99966	ath:AT1G10670|ath:AT5G49460|	837610|835006|	http://www.genome.jp/kegg-bin/show_pathway?ath00020/ath:AT5G49460%09red/ath:AT1G10670%09red

Limonene and pinene degradation	ath00903	2	832584|832585	0.99966	ath:AT5G25130|ath:AT5G25140|	832584|832585|	http://www.genome.jp/kegg-bin/show_pathway?ath00903/ath:AT5G25130%09red/ath:AT5G25140%09red
Protein processing in endoplasmic reticulum	ath04141	8	825225|832267|820102|837252|837365|820091|835701|835699	0.99966	ath:AT3G60540|ath:AT5G22060|ath:AT3G09440|ath:AT1G07400|ath:AT1G08450|ath:AT3G09350|ath:AT5G56010|ath:AT5G56010|	825225|832267|820102|837252|837365|820091|835699|835699|	http://www.genome.jp/kegg-bin/show_pathway?ath04141/ath:AT3G60540%09red/ath:AT1G08450%09red/ath:AT3G09440%09red/ath:AT5G56010%09red/ath:AT5G22060%09red/ath:AT3G09350%09red/ath:AT
Arginine and proline metabolism	ath00330	2	821072|833738	0.99966	ath:AT3G03910|ath:AT5G37600|	821072|833738|	http://www.genome.jp/kegg-bin/show_pathway?ath00330/ath:AT3G03910%09red/ath:AT5G37600%09red
Porphyrin and chlorophyll metabolism	ath00860	1	842198	0.99966	ath:AT1G58290|	842198|	http://www.genome.jp/kegg-bin/show_pathway?ath00860/ath:AT1G58290%09red
Valine, leucine and isoleucine degradation	ath00280	1	830789	0.99966	ath:AT5G09300|	830789|	http://www.genome.jp/kegg-bin/show_pathway?ath00280/ath:AT5G09300%09red
Proteasome	ath03050	1	841815	0.99966	ath:AT1G53780|	841815|	http://www.genome.jp/kegg-bin/show_pathway?ath03050/ath:AT1G53780%09red
Carbon metabolism	ath01200	7	837931|840543|819022|829910|832873|831500|837465	0.99966	ath:AT1G13700|ath:AT1G36370|ath:AT2G44160|ath:AT4G37550|ath:AT5G28030|ath:AT5G16390|ath:AT1G09420|	837931|840543|819022|829910|832873|831500|837465|	http://www.genome.jp/kegg-bin/show_pathway?ath01200/ath:AT1G13700%09red/ath:AT5G28030%09red/ath:AT1G09420%09red/ath:AT5G16390%09red/ath:AT2G44160%09red/ath:AT1G36370%09red/ath:AT
Spliceosome	ath03040	5	830597|820698|830033|831313|820102	0.99966	ath:AT5G07060|ath:AT3G14700|ath:AT4G38780|ath:AT5G14610|ath:AT3G09440|	830597|820698|830033|831313|820102|	http://www.genome.jp/kegg-bin/show_pathway?ath03040/ath:AT3G09440%09red/ath:AT3G14700%09red/ath:AT5G07060%09red/ath:AT4G38780%09red/ath:AT5G1461

Biosynthesis of amino acids	ath01230	7	838847|840543|821003|818260|833613|832873|833738	0.99966	ath:AT1G22410|ath:AT1G36370|ath:AT3G17390|ath:AT2G36880|ath:AT5G36160|ath:AT5G28030|ath:AT5G37600|	838847|840543|821003|818260|833613|832873|833738|	http://www.genome.jp/kegg-bin/show_pathway?ath01230/ath:AT2G36880%09red/ath:AT5G36160%09red/ath:AT5G28030%09red/ath:AT3G17390%09red/ath:AT1G22410%09red/ath:AT5G37600%09red/ath:AT
RNA degradation	ath03018	2	830371|831309	0.99966	ath:AT5G04895|ath:AT5G14580|	830371|831309|	http://www.genome.jp/kegg-bin/show_pathway?ath03018/ath:AT5G14580%09red/ath:AT5G04895%09red
2-Oxocarboxylic acid metabolism	ath01210	1	838210	0.99966	ath:AT1G16400|	838210|	http://www.genome.jp/kegg-bin/show_pathway?ath01210/ath:AT1G16400%09red
Pyruvate metabolism	ath00620	1	831500	0.99966	ath:AT5G16390|	831500|	http://www.genome.jp/kegg-bin/show_pathway?ath00620/ath:AT5G16390%09red
Ribosome biogenesis in eukaryotes	ath03008	1	824963	0.99966	ath:AT3G57940|	824963|	http://www.genome.jp/kegg-bin/show_pathway?ath03008/ath:AT3G57940%09red
Ubiquitin mediated proteolysis	ath04120	2	817943|838564	0.99966	ath:AT2G33770|ath:AT1G19750|	817943|838564|	http://www.genome.jp/kegg-bin/show_pathway?ath04120/ath:AT2G33770%09red/ath:AT1G19750%09red
Glycolysis / Gluconeogenesis	ath00010	1	830867	0.99966	ath:AT5G01320|	830867|	http://www.genome.jp/kegg-bin/show_pathway?ath00010/ath:AT5G01320%09red
mRNA surveillance pathway	ath03015	1	841887	0.99966	ath:AT1G54450|	841887|	http://www.genome.jp/kegg-bin/show_pathway?ath03015/ath:AT1G54450%09red
Oxidative phosphorylation	ath00190	1	836388	0.99966	ath:AT5G62670|	836388|	http://www.genome.jp/kegg-bin/show_pathway?ath00190/ath:AT5G62670%09red
